# Female chronic social defeat stress (*fem*CSDS) maps synaptic, bioenergetic and metabolic proteome remodeling in the nucleus accumbens (NAc)

**DOI:** 10.3389/fsysb.2026.1904427

**Published:** 2026-08-04

**Authors:** Shashikant Patel, Roli Kushwaha, Anushka Arvind, P. V. Anusha, Arvind Kumar, Mohammed M. Idris, Sumana Chakravarty

**Affiliations:** 1 Applied Biology, CSIR-Indian Institute of Chemical Technology, Tarnaka, Hyderabad, India; 2 Academy of Scientific and Innovative Research (AcSIR), Ghaziabad, India; 3 CSIR- Centre for Cellular and Molecular Biology, Habsiguda, Hyderabad, India

**Keywords:** bioenergetics, chronic social defeat stress (CSDS), depression, ingenuity pathway analysis (IPA), mitochondrial dysfunction, nucleus accumbens, string, synaptic plasiticity

## Abstract

**Introduction:**

Depression disproportionately affects women, yet the molecular adaptations underlying stress susceptibility in the female brain remain poorly understood. The nucleus accumbens (NAc) is a key brain region regulating reward, motivation and affective behavior and is strongly implicated in stress-related disorders. This study aimed to characterize proteome-wide molecular adaptations in the female NAc following chronic social stress.

**Methods:**

We used a recently developed female chronic social defeat stress (*fem*CSDS) paradigm and performed label-free quantitative proteomic profiling of the NAc. Differentially expressed proteins were analyzed using Ingenuity Pathway Analysis (IPA), STRING, Metascape, SynGO and transcription factor enrichment approaches to identify affected pathways, molecular networks and regulatory programs.

**Results:**

Proteomic profiling identified 851 significantly dysregulated proteins, comprising 481 downregulated and 370 upregulated proteins, indicating extensive molecular remodeling following chronic social stress. Downregulated proteins were strongly enriched for mitochondrial respiration, oxidative phosphorylation, ATP synthesis, mitochondrial quality-control pathways and metabolic processes. Network analyses identified ATP synthase subunits, respiratory chain components and Cullin-family proteins as central hubs within a highly interconnected mitochondrial-proteostatic circuit. In contrast, upregulated proteins preferentially clustered within synaptogenesis, glutamatergic signaling, endocytosis, vesicle trafficking and synaptic organization pathways. Key hub proteins included SYNJ1, DNM1, BIN1, AP2M1, EPN2 and EPN3. Disease enrichment analyses linked the proteomic signature to neurological, neurodevelopmental and mitochondrial disorders. Transcription factor enrichment and upstream regulator predictions highlighted regulatory programs involving ARNT2, ESRRA, NFE2L2, PIN1, CAMTA1 and MYT1L.

**Discussion:**

This study provides a comprehensive proteomic profile of the female NAc following female chronic social defeat stress (*fem*CSDS). Our analysis indicates that *fem*CSDS may induce widespread molecular remodeling in the female NAc characterized by mitochondrial dysfunction, reduced energetic capacity and altered metabolic regulation alongside enhanced synaptic remodeling. These results provide insight into female-specific stress adaptations and identify molecular pathways that may contribute to depression-related behavioral outcomes and represent potential targets for future investigation.

## Graphical Abstract

**Figure:**
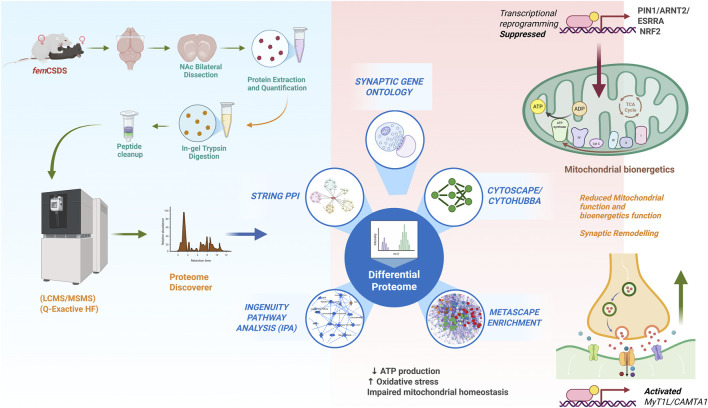

## 1 Introduction

Depression represents a global health crisis with a striking sex disparity; women are diagnosed at twice the rate of men (Piccinelli and Wilkinson, 2000; Williams et al., 2022; Patel et al., 2026). Despite this gap, the neurobiological mechanisms of female-specific vulnerability remain unclear due to historical reliance on male-centric research. Traditionally, the Chronic Social Defeat Stress (CSDS) paradigm has been the gold standard for studying stress-induced phenotypes in rodents, as it effectively replicates the psychosocial stress and reward circuitry dysfunction seen in human patients. However, applying CSDS to females has been difficult because female mice lack the innate aggression required for the resident-intruder setup (Trainor et al., 2011; Yohn et al., 2019; Patel et al., 2025b). Past attempts to solve this often relied on artificial triggers like applying male urine to females or using optogenetic stimulation which risk introducing confounding hormonal and sensory variables (Takahashi et al., 2017; Harris et al., 2018).

Overcoming these barriers, recently we established and validated a novel female chronic social defeat stress (*fem*CSDS) model utilizing persistently aggressive parous CD1 females (Patel et al., 2026). Defeated females exhibited robust social avoidance, anhedonia, behavioral despair, anxiety-like behavior, elevated corticosterone levels and glutamatergic dysregulation in the nucleus accumbens and caudate putamen, supporting the face validity of the model as a female-specific analogue of the traditional male CSDS paradigm. This paradigm allows for a direct, investigation into the proteomic signatures that define stress susceptibility in the female brain. The NAc is a central component of the mesolimbic reward circuit and plays a critical role in reward processing, motivation and social behavior. Clinical and preclinical studies have consistently implicated NAc dysfunction in depression, particularly in symptoms such as social withdrawal and reduced motivation. Chronic stress can alter neuronal activity, synaptic plasticity and neurotransmitter signaling within the NAc, leading to persistent behavioral abnormalities. Understanding the molecular adaptations occurring within this region is therefore essential for identifying mechanisms that drive stress susceptibility and depression-like phenotype.

In male models, the NAc is a primary site of stress-induced changes in synaptic transmission and mitochondrial energy production (Bagot et al., 2015; Xu et al., 2024). Although several studies have investigated molecular mechanisms of chronic stress in male rodents, relatively little is known about proteome-wide alterations occurring in the female NAc following chronic social defeat. Most previous studies have focused on transcriptomic changes or have been conducted exclusively in males, leaving a substantial gap in our understanding of female-specific molecular responses. Since women often report different symptom clusters and treatment outcomes than men, it is essential to determine if the molecular shifts in the female NAc follow common pathways or involve distinct sex-divergent mechanisms.

Utilizing a high-resolution proteomic approach allows for a comprehensive investigation into how converged pathways of oxidative stress, metabolic failure and synaptic instability drive the depression-like phenotypes of anhedonia and social withdrawal in females. In this work, we present a comprehensive proteomic map of the female NAc following chronic social defeat. We identified 851 significantly dysregulated proteins, suggestive of complex association of glutamatergic signaling, mitochondrial failure and synaptic remodeling.

## 2 Materials and methods

### 2.1 Brain area sampling and tissue procurement

Following the completion of the *fem*CSDS paradigm as previously described (3), involving 10 days of repeated social defeat exposure to selected aggressive parous CD1 females with overnight sensory contact through a perforated divider, defeated female C57BL/6NCrL mice underwent a behavioral test battery prior to sacrifice via cervical dislocation. NAc from defeated mice were dissected. In brief, to preserve proteomic integrity, brains were rapidly harvested and rinsed in ice-cold phosphate-buffered saline (PBS) before placement in a chilled brain matrix (Harvard Apparatus, United States). Coronal sections (1.0 mm thickness) were prepared, and the NAc was bilaterally microdissected using a 12G stainless steel puncher, with four punches collected per brain to ensure sufficient protein yield. Tissues were immediately snap-frozen in liquid nitrogen and stored at −80 °C until further processing. All animal experiments were performed at the CSIR-CCMB animal house in Hyderabad, India. Protocols used in this study were approved by the institutional Animal Ethics Committee under protocol numbers [#IAEC/62/2022–23] and [#IAEC 70/2022–23]. For the proteomic study, a total of 16 mice were utilized, comprising 8 control female and 8 defeated female mice. Samples included 2 control groups and two defeated groups run in duplicates with each group consisting of pooled samples from 4 individual mice. This sample size was selected based on our previously established *fem*CSDS model and is consistent with sample sizes commonly employed in discovery-based label-free quantitative proteomic studies to achieve proteome coverage while maintaining biological reproducibility.

### 2.2 Preparation of tissue lysates and protein solubilization

Total protein was extracted using a high-molarity urea-based solubilization buffer [8 M urea, 2 M thiourea, 18 mM Tris HCl, 4% CHAPS, 14 mM Trizma base, 0.2% Triton X-100, 50 mM dithiothreitol and Complete™ ethylenediaminetetraacetic acid (EDTA)-free protease inhibitor cocktail (Roche, Germany), pH 7.4] to ensure the recovery of both cytosolic and membrane-bound proteins (Saxena et al., 2012). Following homogenization and solubilization, protein concentrations were quantified using the amido black assay, with bovine serum albumin (BSA) serving as the standard for the calibration curve (Schaffner and Weissmann, 1973).

### 2.3 SDS-PAGE and in-gel digestion

Label-free Quantitative (LFQ) MS-MS analysis was performed in both experimental and biological duplicates. For each group, 100 µg of total protein underwent electrophoretic separation on 10% SDS-PAGE gels. Following electrophoresis, gels were stained with Coomassie Brilliant Blue R250 and subsequently destained to visualize protein bands. Each lane was then excised into four distinct fractions based on molecular weight to reduce sample complexity and enhance the depth of protein identification. These fractions underwent in-gel digestion using sequencing-grade trypsin to generate a complex peptide mixture.

### 2.4 Peptide purification and LC-MS/MS analysis

Trypsin-digested peptides were purified using C-18 spin columns (Thermo Scientific) to remove salts and detergents that might interfere with mass spectrometry. Purified peptides were then reconstituted in a mobile phase consisting of 5% acetonitrile (ACN) and 0.2% formic acid. The samples were analyzed via Liquid Chromatography-Tandem Mass Spectrometry (LC-MS/MS) using Orbitrap Q-Exactive HF. Peptides were separated on a nano-flow LC system and ionized via electrospray ionization before entering the mass spectrometer for high-resolution MS and MS/MS scans.

### 2.5 Ingenuity Pathway Analysis (IPA)

To elucidate the functional landscape and hierarchical regulatory networks of the NAc in response to stress, the differentially expressed protein dataset was analyzed using Ingenuity Pathway Analysis (IPA) (Krämer et al., 2014). Canonical pathway analysis was performed to identify enriched signaling and metabolic pathways. Furthermore, we utilized the “Disease and Biofunction” feature to categorize proteins according to their roles in neurological disorders, metabolic production and cellular maintenance. Causal network analysis and upstream regulator predictions were employed to identify master molecular drivers providing insights into the predicted activation or inhibition states of these networks. Top regulator effect networks were ranked by consistency scores to highlight the most statistically robust drivers of the NAc dysfunction.

### 2.6 STRING functional enrichment and network architecture

Protein-Protein Interaction (PPI) networks were constructed using the Search Tool for the Retrieval of Interacting Genes/Proteins (STRING) database (version 12.0) to visualize functional clusters and connectivity among dysregulated proteins (Szklarczyk et al., 2023). To preserve biological clarity and avoid masking opposing directional changes, significantly downregulated and upregulated protein sets were analyzed as distinct cohorts. The network mapping utilized high-confidence settings, including a minimum required interaction score of 0.700. Functional enrichment analysis was performed across Gene Ontology (GO) categories, Biological Process, Molecular Function, and Cellular Component as well as KEGG and Reactome pathways. To define specific functional modules within the NAc proteome, we applied MCL (Markov Cluster Algorithm) with an inflation parameter of 3.0 and K-means clustering.

### 2.7 Network topology and hub analysis

To identify critical structural and functional hubs within the stress-responsive proteome, network topology analysis was performed using Cytoscape (v3.9.1+) and the CytoHubba plugin. Multiple centrality parameters were evaluated, including Degree Centrality, Betweenness Centrality and Maximal Clique Centrality (MCC). The MCC algorithm was specifically utilized to identify “essential hubs” embedded within densely interconnected subnetworks. MCC prioritizes proteins participating in highly cohesive clique structures and is particularly effective for identifying essential components of multi-subunit molecular complexes. Proteins were ranked according to MCC score,and the top-ranked hub proteins were extracted separately for upregulated and downregulated networks.

To integrate network topology with expression magnitude, module-specific impact analysis was performed by mapping Log2 fold-change values onto Louvain-derived functional modules. The distribution of expression changes across major functional modules was visualized using ridge density plots generated with the ggridges package in R. Core subnetworks were visualized using force-directed stress layouts generated with the ggraph package in R. To investigate pathway-level functional convergence and biological crosstalk, Gene-Concept Networks (Cnetplots) were generated using the enrichplot package in R (Yu and Gao, 2025).

### 2.8 Upstream transcriptional regulatory and functional enrichment analysis

To identify transcriptional regulatory programs and potential upstream transcription factors associated with the observed proteomic alterations, ChEA3 (ChIP-X Enrichment Analysis 3) was utilized (Keenan et al., 2019). Candidate upstream transcriptional regulators were ranked using integrated mean-rank scoring, enabling identification of potential regulatory drivers.

Metascape analysis was performed using independently uploaded upregulated and downregulated protein datasets to identify enriched biological pathways, functional processes and protein–protein interaction networks (Zhou et al., 2019). Enrichment significance was determined using hypergeometric testing followed by Benjamini–Hochberg false discovery rate correction. Enriched terms were hierarchically clustered based on Kappa similarity scores to identify higher-order functional modules. Protein–protein interaction enrichment analysis was subsequently performed using integrated STRING-based interaction datasets within Metascape, followed by Molecular Complex Detection (MCODE) clustering to identify densely interconnected subnetworks. Because most enrichment platforms operate at the gene level, differentially expressed proteins were first mapped to their corresponding gene symbols, which were subsequently used for analyses. ShinyGO analysis was performed for Gene Ontology enrichment, pathway visualization, hierarchical clustering, tissue-specific enrichment analysis and functional annotation profiling (Ge et al., 2020).

### 2.9 Synaptic gene ontology (SynGO) enrichment analysis

Synaptic Gene Ontology (SynGO) enrichment analysis was performed to investigate synapse specific biological processes and cellular components associated with the differentially expressed proteins. Gene symbols corresponding to the combined, upregulated and downregulated proteomic datasets were analyzed separately using the SynGO web platform (version 1.3; https://www.syngoportal.org) (Koopmans et al., 2019). Enrichment analysis was performed using the brain-expressed gene background provided by SynGO and significance was assessed using a false discovery rate (FDR) threshold of 1%. Enriched SynGO Cellular Component and Biological Process terms were identified, and the resulting ontology distributions were visualized using sunburst plots. SynGO-annotated genes and significantly enriched terms were subsequently compared across the combined, upregulated and downregulated datasets to identify synaptic compartments and functional pathways preferentially affected by *fem*CSDS.

### 2.10 Proteomic data processing and bioinformatic pipeline

Protein identification and label-free quantification (LFQ) were conducted using the Thermo Scientific Proteome Discoverer (version 2.2.3) platform, employing the Sequest HT search engine to query the *Mus musculus* proteome. Data analysis employed the XCorr (Score Vs. Charge) algorithm and high identification confidence was ensured by applying a Target-Decoy False Discovery Rate (FDR) approach. The reliability of these identifications was further validated through the Sum PEP Score (Posterior Error Probability) and the total number of Peptide Spectrum Matches (PSMs). Differential expression was assessed by calculating the arithmetic mean of normalized abundances for the Control and Stress groups. These averages were then used to determine the Log2 Fold Change (Log2 FC), a transformation that allows for a symmetrical and linear comparison of upregulated and downregulated proteins.

Low-confidence and ambiguously annotated entries, including unnamed proteins, hypothetical proteins, predicted proteins, partial proteins, accession-only entries and cross-species contaminants were removed from further analysis. Multiple isoforms and duplicate protein entries mapping to the same biological gene were manually inspected and consolidated into unique gene-level representations to eliminate redundancy and prevent artificial inflation of pathway enrichment and interaction network analyses. Unless otherwise indicated, molecular identifiers reported in STRING, IPA, Metascape, SynGO and transcription-factor enrichment analyses are presented as standardized gene symbols representing the corresponding protein products. Final classification of the protein list was based on a strict biological significance threshold (|Log2FC| ≥ 0.5). Following stringent de-redundancy and annotation refinement, the final curated non-redundant dataset consisted of 851 unique high-confidence proteins.

## 3 Results

### 3.1 M/MS proteomic landscape of the NAc following *fem*CSDS

**FIGURE 1:**
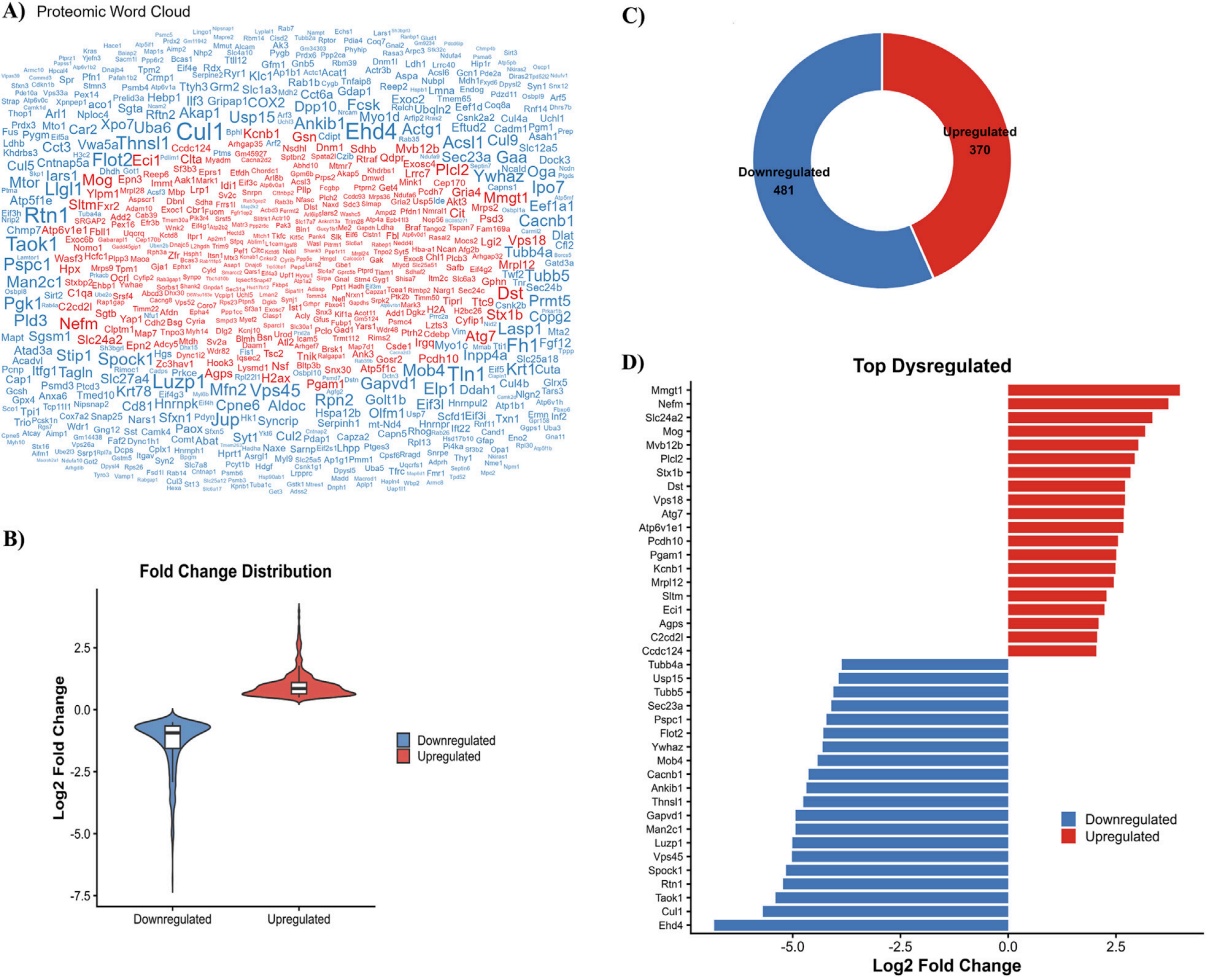
Global proteomic alterations in the nucleus accumbens following female chronic social defeat stress (*fem*CSDS). **(A)** Word cloud representation of differentially expressed proteins identified in the NAc following *fem*CSDS. Protein size reflects relative abundance of differential expression, while color indicates direction of regulation (red, upregulated; blue, downregulated). **(B)** Violin plot showing the distribution of Log2 fold-change values for downregulated and upregulated proteins. The downregulated proteome exhibited a broader distribution and larger magnitude of expression changes compared with upregulated proteins. **(C)** Proportion of significantly dysregulated proteins identified by label-free quantitative proteomics, showing 481 downregulated and 370 upregulated proteins **(D)** Top differentially expressed proteins ranked according to Log2 fold-change. Red bars represent upregulated proteins and blue bars represent downregulated proteins.

Initial proteomic profiling identified 1,194 differentially expressed protein entries. Following stringent data filtering, annotation refinement, removal of low-confidence and ambiguously annotated proteins and consolidation of redundant isoforms mapping to the same biological gene, a final curated non-redundant dataset comprising 851 differentially expressed proteins was obtained for downstream analyses. A global overview of the dataset is shown in Figure 1A. The overall distribution of fold changes showed a larger proportion of downregulated proteins compared with upregulated proteins (Figure 1B). Several proteins exhibited substantial reductions in expression, whereas increases in expression were generally of lower magnitude. This pattern suggests that *fem*CSDS is associated with broad suppression of multiple protein populations within the NAc. 481 proteins were downregulated and 370 proteins were upregulated in the defeated group (Figure 1C). Dysregulated proteins belonged to diverse functional classes and included proteins involved in cellular signaling, intracellular trafficking, metabolism, structural organization and synaptic function. Examination of the most strongly altered proteins revealed marked decreases in proteins corresponding to EHD4, CUL1, TAOK1 and RTN1, while proteins corresponding to MMGT1, NEFM, SLC24A2, and MOG were among the most highly increased (Figure 1D).

### 3.2 STRING network analysis identifies coordinated functional remodeling following *fem*CSDS

To determine whether the proteins altered by *fem*CSDS were organized into coordinated biological systems, separate protein-protein interaction (PPI) networks were constructed for downregulated and upregulated proteins using the STRING database (v12.0).

#### 3.2.1 STRING network analysis identifies distinct functional modules within the dysregulated proteome

**FIGURE 2:**
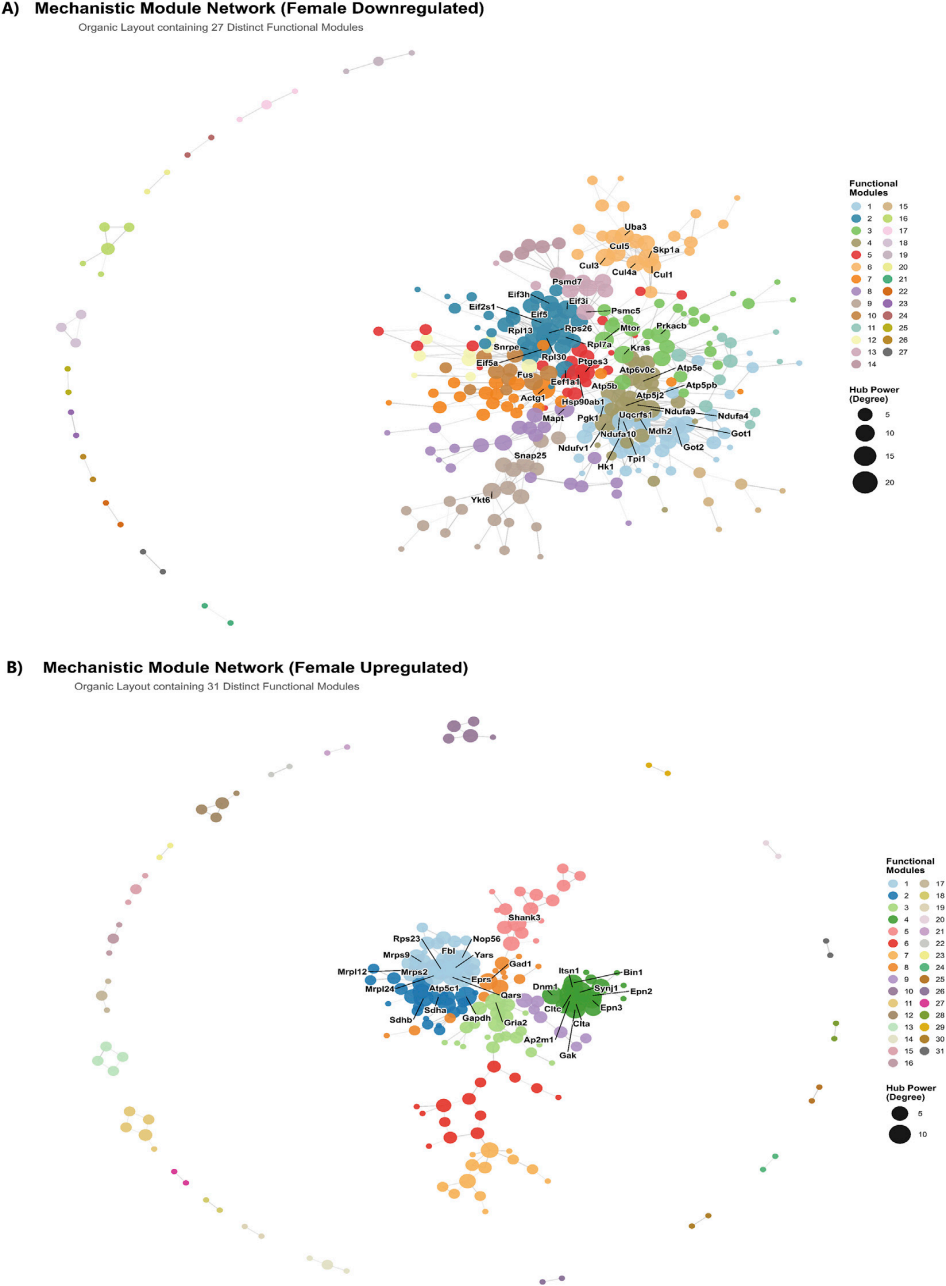
STRING protein–protein interaction networks reveal distinct functional module architecture in the *fem*CSDS responsive proteome. **(A)** STRING interaction network of downregulated proteins following *fem*CSDS: Proteins are organized into 27 Louvain-derived functional modules, represented by different colors. Node size corresponds to hub power (degree centrality), with larger nodes indicating more highly connected proteins. The downregulated interactome formed a densely interconnected core enriched in mitochondrial respiration, oxidative phosphorylation, protein degradation, translational regulation and intracellular signaling pathways. Major hub nodes included ATP synthase subunits, respiratory chain components and Cullin-associated proteostasis regulators. **(B)** STRING interaction network of upregulated proteins following *fem*CSDS: Proteins were partitioned into 31 distinct functional modules. The upregulated interactome exhibited a more compact central architecture with multiple smaller peripheral subnetworks. Major hubs were associated with clathrin-mediated endocytosis, membrane trafficking, synaptic organization and ribosomal function.

Network organization was subsequently examined using Louvain community detection, which partitions highly interconnected proteins into distinct functional modules based on interaction density. The downregulated interactome was resolved into 27 functional modules, whereas the upregulated interactome was partitioned into 31 modules (Figures 2A,B). Although both networks exhibited clear modular organization, notable differences were observed in their overall architecture. The downregulated network formed a large interconnected structure with multiple modules converging around a densely connected central core, while the upregulated network displayed a more compact central organization surrounded by several smaller peripheral communities. The downregulated interactome revealed extensive clustering of proteins involved in mitochondrial function, oxidative phosphorylation, protein degradation, translation, intracellular signaling and cytoskeletal regulation (Figure 2A). Several prominent modules contained mitochondrial electron transport chain proteins, including NDUFA9, NDUFA10, NDUFV1 (Complex I), UQCRFS1 (Complex III) and NDUFA4 (Complex IV), together with ATP synthase (Complex V) subunits ATP5B, ATP5E, and ATP5PB. Additional interconnected communities were enriched in Cullin family proteins (CUL1–CUL5), UBA3, SKP1A, translation initiation factors (EIF2S1, EIF3I, EIF5) and ribosomal proteins including RPL13, RPL30 and RPS26. While assigned to separate Louvain communities, these modules remained linked through multiple interaction bridges, indicating substantial cross-talk between pathways involved in energy metabolism, proteostasis and protein synthesis.

In contrast, the upregulated interactome was dominated by modules associated with synaptic organization, membrane trafficking, endocytosis, ribosomal biology and amino acid metabolism (Figure 2B). Central regions of the network were organized around proteins involved in clathrin-mediated trafficking and vesicle dynamics, including AP2M1, CLTC, CLTA, DNM1, EPN2, EPN3, GAK, BIN1 and SYNJ1. Additional communities contained synaptic proteins such as GRIA2 and SHANK3, together with mitochondrial and cytosolic ribosomal proteins including MRPL12, MRPL24, MRPS2, MRPS9 and RPS23. Compared with the downregulated network, the upregulated interactome exhibited a greater number of smaller functionally specialized subnetworks extending from the central community.

#### 3.2.2 Functional characterization of STRING-derived protein interaction networks

STRING enrichment analysis independently supported the distinct functional profiles of the upregulated and downregulated interactomes (Supplementary Figure 1). A more comprehensive Gene Ontology enrichment analysis confirmed the predominance of bioenergetic and mitochondrial deficits among downregulated proteins, with significant enrichment of ATP metabolism, purine nucleotide metabolism, aerobic respiration, proton transmembrane transport and energy-generating pathways (Supplementary Figure 2). Gene-concept network analysis further demonstrated extensive overlap among these enriched processes, with multiple GO terms converging on shared mitochondrial and ATP-generating proteins (Supplementary Figure 3). Among downregulated proteins, the most prominent clusters were associated with the mitochondrial respiratory chain, oxidative phosphorylation, ATP synthase complexes, RNA-binding and mRNA stability machinery, cytoplasmic translation, Cullin-dependent ubiquitin signaling and mitochondrial translation. In contrast, upregulated proteins formed clusters enriched for clathrin-mediated endocytosis, mitochondrial translation elongation, aminoacyl-tRNA biosynthesis and SNARE-mediated neurotransmitter release pathways (Supplementary Figure 4). Consistent with these findings, integrative module-impact and functional-switch analyses suggested of a coordinated downregulation of mitochondrial and proteostasis machinery together with increased expression of synaptic and ribosomal proteins, indicative of stress-associated bioenergetic-to-synaptic functional shift (Supplementary Figure 5).

### 3.3 Identification of essential hub proteins and network bottlenecks

**FIGURE 3:**
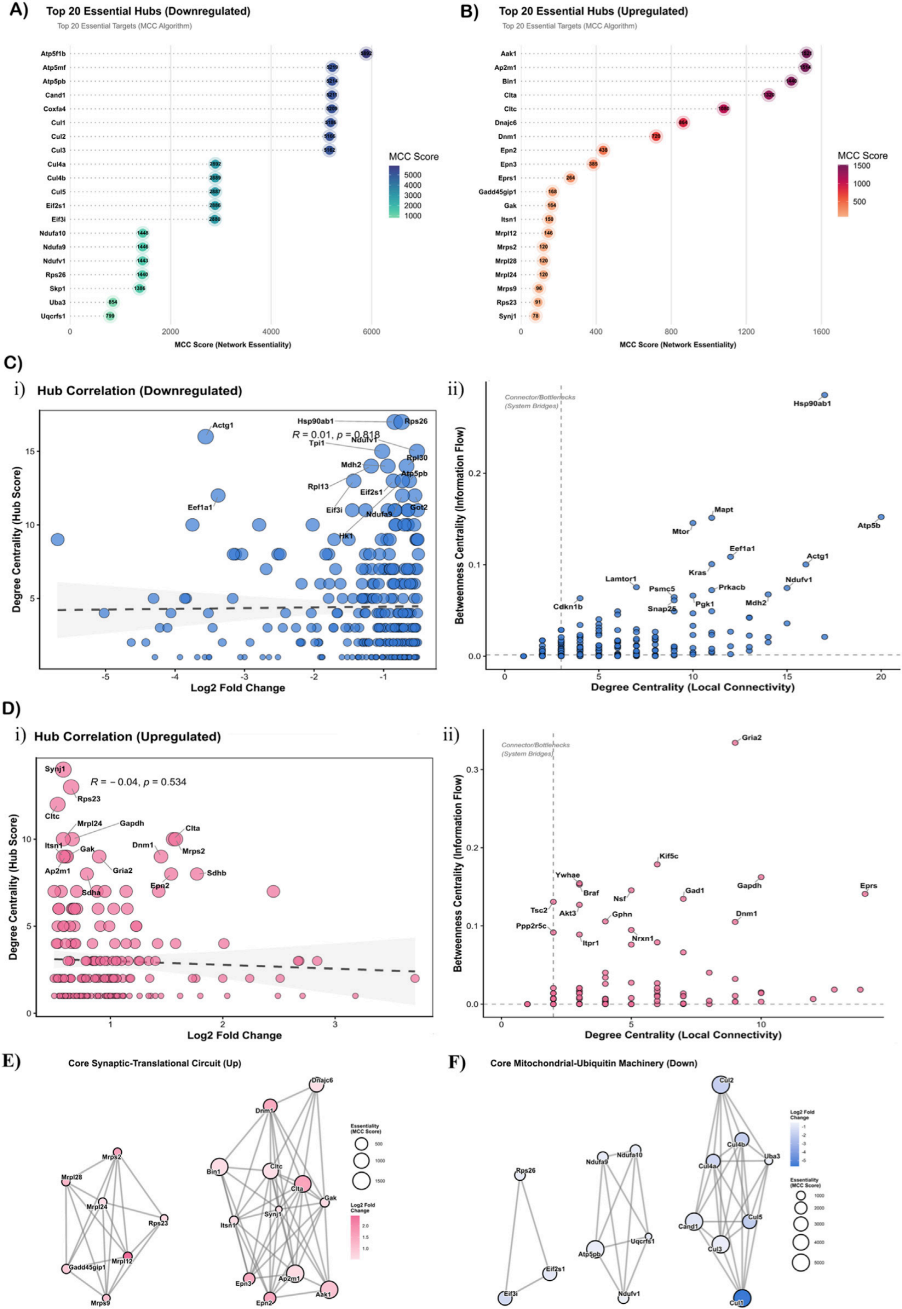
Network centrality analysis identifies key hub proteins and core molecular circuits associated with *fem*CSDS. **(A,B)** Top 20 hub proteins identified using the Maximal Clique Centrality (MCC) algorithm within the downregulated **(A)** and upregulated **(B)** STRING interaction networks. Downregulated hubs were dominated by mitochondrial respiratory chain components, ATP synthase subunits, Cullin family proteins, ubiquitin pathway regulators and translation-associated proteins, whereas upregulated hubs were enriched for proteins involved in synaptic vesicle trafficking, endocytosis, membrane remodeling and ribosomal function. **(C,D)** Relationships between protein expression changes and network topology. Correlation analyses (i) demonstrate no significant relationship between fold change and degree centrality in either downregulated or upregulated networks. Degree-betweenness centrality plots (ii) identify highly connected proteins and potential network bottlenecks that mediate information flow between functional modules. **(E)** Core synaptic-translational subnetwork extracted from the upregulated interactome, highlighting interactions among endocytic, vesicle-trafficking and ribosomal proteins. Node size represents MCC essentiality score and node color indicates Log2 fold change. **(F)** Core mitochondrial-ubiquitin subnetwork extracted from the downregulated interactome, illustrating coordinated suppression of oxidative phosphorylation, proteostasis and ubiquitin-dependent regulatory machinery.

To identify topologically essential proteins within the *fem*CSDS associated interactomes, hub analysis was performed using the CytoHubba plugin in Cytoscape employing MCC algorithm. Additional network analyses were performed to examine relationships between protein abundance changes and topological importance, as well as to identify connector proteins that potentially mediate information flow between distinct functional modules. MCC analysis revealed marked differences between the downregulated and upregulated interactomes (Figures 3A,B). Within the downregulated network, the highest-ranking essential hubs were dominated by proteins associated with mitochondrial respiration, ATP synthesis, ubiquitin-mediated protein degradation and translational control. The top-ranked hub nodes included ATP5F1B, ATP5MF, ATP5PB, COX4I1, CUL1-CUL5, CAND1, EIF2S1, EIF3I, NDUFA9, NDUFA10, NDUFV1, UBA3, SKP1, RPS26 and UQCRFS1. Notably, ATP synthase subunits and Cullin family proteins occupied the highest MCC positions, indicating that mitochondrial energy production and ubiquitin-dependent proteostasis represent major structural components of the downregulated interactome. In contrast, the upregulated interactome was enriched for hubs involved in endocytosis, vesicle trafficking, synaptic organization and translational machinery (Figure 3B). The highest MCC scores were observed for AAK1, AP2M1, BIN1, CLTA, CLTC, DNAJC6, DNM1, EPN2, EPN3, GAK, ITSN1, SYNJ1 and multiple ribosomal proteins including RPS23, MRPL12, MRPL24, MRPL28, MRPS2 and MRPS9. These findings suggest that proteins regulating clathrin-mediated membrane trafficking and synaptic vesicle dynamics constitute the principal organizational centers of the upregulated network.

To determine whether network importance was related to the magnitude of protein dysregulation, degree centrality was compared with Log2 fold-change values. No significant relationship was observed in either the downregulated (R = 0.01, p = 0.818) or upregulated (R = −0.04, p = 0.534) interactome (Figures 3C-i, D-i), indicating that proteins occupying central network positions were not necessarily those exhibiting the largest expression changes. Network topology analysis further identified a subset of proteins with elevated betweenness centrality that functioned as potential communication bridges between modules (Figures 3C-ii, D-ii). Within the downregulated interactome, ATP5B, NDUFV1, ACTG1, EEF1A1, MAPT, MTOR, MDH2 and HSP90AB1 displayed prominent bottleneck characteristics, occupying positions that linked multiple subnetworks. In the upregulated interactome, GRIA2 emerged as the strongest connector node, exhibiting the highest betweenness centrality, followed by EPRS, DNM1, GAD1, KIF5C, NSF, YWHAE and BRAF.

### 3.4 Core molecular circuits revealed by essential hub interactions

The downregulated core network formed a densely interconnected molecular circuit dominated by mitochondrial and ubiquitin-associated proteins (Figure 3F). ATP synthase components (ATP5F1B, ATP5MF, ATP5PB) clustered together with respiratory chain proteins including NDUFA9, NDUFA10, NDUFV1 and UQCRFS1, forming a prominent bioenergetic module. This mitochondrial cluster was directly linked to a second interconnected module composed of Cullin family proteins (CUL1–CUL5), CAND1 and UBA3, forming the core Cullin-RING E3 ubiquitin ligase machinery responsible for ubiquitin-dependent protein turnover. Translational regulators including EIF2S1, EIF3I and RPS26 were integrated within this network. The extensive connectivity observed among these proteins indicates that mitochondrial respiration, protein quality control and translational regulation constitute a coordinated molecular circuit affected by *fem*CSDS.

In contrast, the upregulated core network resolved into a synaptic-translational circuit centered on proteins involved in endocytosis, membrane trafficking and protein synthesis (Figure 3E). A highly connected cluster containing CLTC, CLTA, AP2M1, DNM1, DNAJC6, EPN2, EPN3, GAK, ITSN1, SYNJ1, BIN1 and AAK1 formed the dominant structural module. This complex was linked to a second module enriched in ribosomal proteins including RPS23, MRPL12, MRPL24, MRPL28, MRPS2 and MRPS9. The physical integration of these modules suggests close coordination between synaptic vesicle recycling pathways and translational machinery.

### 3.5 Coordinated alterations in mitochondrial and synaptic protein families

**FIGURE 4:**
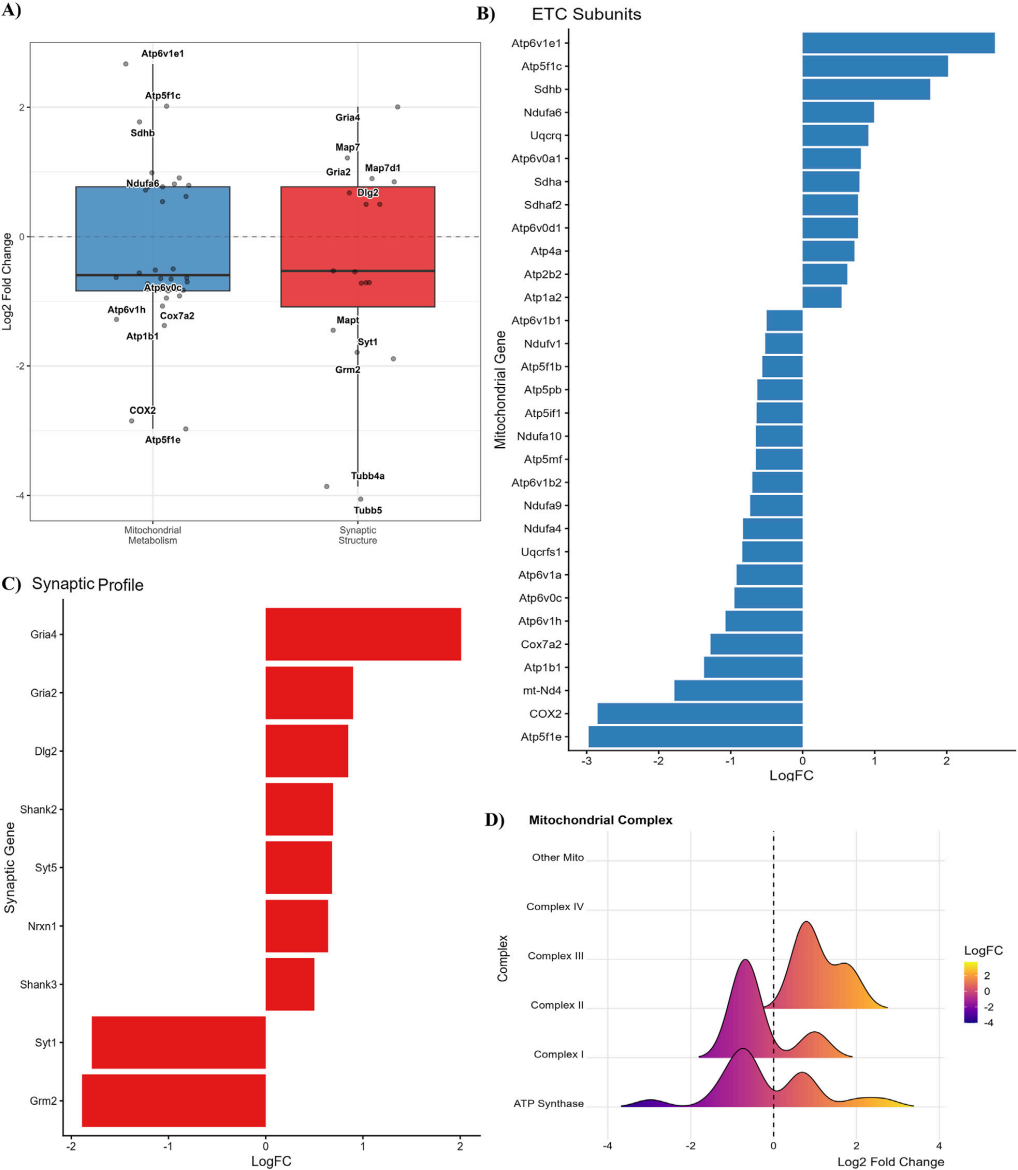
Coordinated alterations in mitochondrial metabolism and synaptic structural proteins following *fem*CSDS. **(A)** Comparison of Log2 fold-change distributions for selected mitochondrial bioenergetic proteins and synaptic structural proteins: Mitochondrial proteins were predominantly downregulated, whereas synaptic proteins displayed a mixed profile with several postsynaptic markers showing increased expression. Individual proteins of interest are annotated. **(B)** Differential expression of mitochondrial oxidative phosphorylation (OXPHOS) proteins: Multiple components of the electron transport chain and ATP synthase exhibited reduced abundance, including ATP synthase (Complex V) subunits, Complex I proteins (NDUFA/NDUFV family members), the Complex III subunit UQCRFS1, and Complex IV-associated proteins. **(C)** Expression profile of representative synaptic proteins: Increased expression of postsynaptic proteins including GRIA2, GRIA4, DLG2, SHANK2, SHANK3, NRXN1, and SYT5 contrasted with reduced expression of SYT1 and GRM2. **(D)** Distribution of fold changes across mitochondrial functional classes: ATP synthase (Complex V) subunits and electron transport chain complexes exhibited a predominance of negative fold changes, with the strongest suppression observed among ATP synthase and Complex IV-associated proteins.

To further investigate the biological systems emerging from the network analyses, representative proteins associated with mitochondrial energy metabolism and synaptic structure were examined separately (Figures 4A–D). Comparison of the two groups revealed distinct expression patterns. Mitochondrial proteins showed an overall negative shift in fold change distribution, whereas synaptic proteins exhibited a broader range of regulation with both increased and decreased components (Figure 4A). Although substantial variability was observed within both groups, mitochondrial proteins were predominantly represented by negative fold changes, suggesting coordinated remodeling of energy-related pathways.

#### 3.5.1 Mitochondrial respiratory chain proteins are broadly altered

Examination of individual mitochondrial proteins demonstrated that changes were distributed across multiple components of the mitochondrial oxidative phosphorylation system and ATPase machinery (Figure 4B). Several proteins involved in ATP synthesis and proton transport displayed marked reductions, including ATP synthase subunits (encoded by ATP5F1E, ATP5PB and ATP5F1B), vacuolar ATPase components (encoded by ATP6V1H, ATP6V0C, and ATP6V1A) and the *Na+/K +* ATPase subunit ATP1B1. Similarly, Complex I subunits NDUFA9, NDUFA10, NDUFA6 and NDUFV1, together with the Complex III subunit UQCRFS1 and the Complex IV subunits NDUFA4 and COX7A2 also showed negative fold changes. In contrast, a smaller subset of mitochondrial proteins, ATP6V1E1, ATP5F1C, SDHB, SDHA and SDHAF2, exhibited positive regulation. Grouping proteins according to respiratory chain complexes further indicated that ATP synthase and Complex I-associated proteins accounted for much of the observed negative shift, whereas other complexes displayed a more heterogeneous distribution (Figure 4D).

#### 3.5.2 Selective remodeling of synaptic protein networks

Synaptic proteins exhibited bidirectional regulation (Figure 4C). Positive changes were observed for GRIA4, GRIA2, DLG2, SHANK2, SHANK3, SYT5 and NRXN1, while GRM2 and SYT1 were reduced. Among these proteins, GRIA4 showed the strongest positive fold change, whereas GRM2 represented the most prominent negative change. These findings indicate that *fem*CSDS is associated with coordinated alterations in mitochondrial respiratory proteins alongside selective remodeling of proteins involved in synaptic organization and signaling.

#### 3.5.3 Functional crosstalk and pathway-level mapping suggests coordinated mitochondrial and synaptic remodeling

Functional crosstalk analysis of downregulated proteins formed a highly interconnected network centered on oxidative phosphorylation and neurodegeneration-associated pathways, including Parkinson’s disease, Alzheimer disease, amyotrophic lateral sclerosis and prion disease, which shared numerous mitochondrial and proteostasis-related proteins such as NDUFA4, NDUFA9, NDUFA10, NDUFV1, ATP5F1B, ATP5PB, UQCRFS1 and MAPT (Supplementary Figure 6A). In contrast, upregulated proteins were organized into smaller, functionally specialized modules dominated by endocytosis, synaptic vesicle cycling, dopaminergic synapse signaling and calcium-regulated transport processes (Supplementary Figure 6B), consistent with enhanced synaptic remodeling and vesicle trafficking.

Pathway-level visualization further supported these observations (Supplementary Figure 7). Mapping of dysregulated proteins onto the oxidative phosphorylation pathway demonstrated coordinated alterations across multiple electron transport chain complexes and ATP synthase subunits, indicating widespread impairment of mitochondrial bioenergetics (Supplementary Figure 7A). Similarly, mapping onto the synaptic vesicle cycle pathway revealed alterations in proteins involved in neurotransmitter release, SNARE-complex function, vesicle docking, endocytosis and vesicle recycling, including SNAP25, STX1B, SYT1, AP2M1, CLTA, DNM1 and NSF (Supplementary Figure 7B).

### 3.6 Metascape enrichment reveals divergent biological programs in downregulated and upregulated networks

**FIGURE 5:**
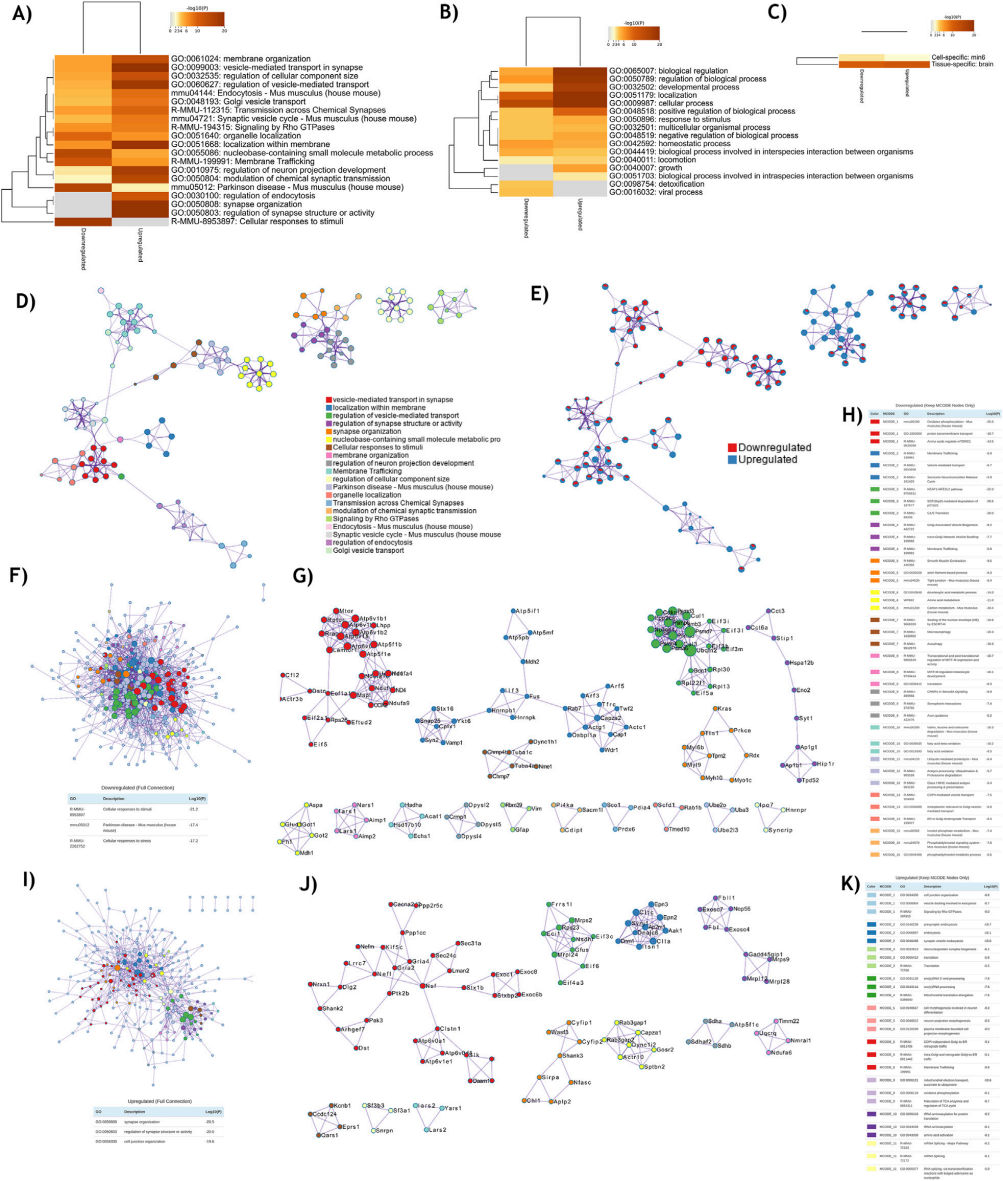
Functional enrichment and network topology analyses reveal distinct biological signatures of the downregulated and upregulated proteomes. **(A,B)** Heatmap representation of significantly enriched Gene Ontology (GO) biological processes for downregulated **(A)** and upregulated **(B)** proteins. Color intensity represents enrichment significance (-log10 adjusted *P* value). **(C)** Tissue and cell-type enrichment analysis demonstrating significant enrichment of brain-associated and neuronal signatures among dysregulated proteins. **(D,E)** Functional interaction networks generated from enriched GO terms. **(D)** Nodes are colored according to enriched biological processes, illustrating the clustering of related functional modules. **(E)** Nodes are colored according to direction of expression change (red, downregulated; blue, upregulated), highlighting the distribution of dysregulated proteins across interconnected biological pathways. **(F,G)** Downregulated protein interaction network and its corresponding modular decomposition. The network is dominated by clusters associated with mitochondrial respiration, oxidative phosphorylation, intracellular trafficking, translation, proteostasis and metabolic regulation. Representative functional modules are shown in **(G)**. **(H)** Labelling of enriched functional modules identified within the downregulated interactome. **(I,J)** Upregulated protein interaction network and corresponding functional modules. Enriched clusters were primarily associated with synaptic organization, vesicle trafficking, membrane dynamics, cytoskeletal remodeling, ribosomal processes and neuronal signaling pathways. Representative subnetworks are shown in **(J)**. **(K)** Labelling of enriched functional modules identified within the upregulated interactome.

To further characterize the biological processes represented within the *fem*CSDS responsive proteome, pathway enrichment and protein interaction module analyses were performed using Metascape. Hierarchical clustering of enriched terms revealed substantial functional overlap between downregulated and upregulated proteins, with shared enrichment for vesicle-mediated transport, membrane organization, membrane trafficking, synaptic signaling, regulation of neuron projection development, endocytosis and Golgi-associated transport processes (Figures 5A–C). However, distinct functional biases were also evident between the two expression groups. Downregulated proteins showed stronger enrichment for cellular responses to stimuli and stress, metabolic pathways, intracellular trafficking and membrane transport processes, whereas upregulated proteins preferentially clustered within synapse organization, regulation of synapse structure and activity, cell junction organization, endocytosis and synaptic vesicle cycling pathways. Tissue-specific enrichment further demonstrated a strong association with brain-enriched proteins, supporting the neurobiological relevance of the identified molecular alterations (Figure 5C). The enrichment network further demonstrated that these pathways formed interconnected clusters linked through shared gene memberships (Figures 5D,E).

#### 3.6.1 MCODE analysis reveals functionally specialized downregulated subnetworks

To identify densely connected protein communities within the downregulated interactome, MCODE analysis was performed (Figures 5F–H). The most significantly enriched module was associated with oxidative phosphorylation, proton transmembrane transport and amino acid-mediated regulation of mTORC1 signaling. Additional modules were enriched for membrane trafficking, vesicle-mediated transport, serotonin neurotransmitter release, Golgi vesicle biogenesis, autophagy, ubiquitin-mediated proteolysis, fatty acid β-oxidation, amino acid metabolism, phosphatidylinositol signaling, translation and axon guidance pathways. Several modules were also linked to cellular stress-response programs and neurodegeneration-associated pathways. These findings indicate coordinated suppression of mitochondrial bioenergetics, intracellular trafficking, proteostatic regulation, and structural signaling systems within the female NAc following chronic social defeat stress.

#### 3.6.2 Upregulated subnetworks converge on synaptic remodeling and vesicle dynamics

MCODE analysis of the upregulated interactome identified multiple densely connected subnetworks primarily associated with synaptic and membrane-trafficking functions (Figures 5I–K). The dominant modules were enriched for synapse organization, regulation of synapse structure and activity, presynaptic endocytosis, synaptic vesicle endocytosis, vesicle docking during exocytosis and cell junction organization. Additional subnetworks were associated with neuronal morphogenesis, ribonucleoprotein complex biogenesis, translation, RNA processing, Golgi-to-ER trafficking, mitochondrial electron transport, tRNA aminoacylation, amino acid activation and mRNA splicing pathways. Among these, synaptic vesicle cycling and endocytic pathways exhibited the strongest enrichment signals, indicating enhanced regulation of synaptic membrane turnover and neurotransmission-related processes.

### 3.7 Canonical pathway analysis: synaptic, metabolic and neurodegenerative dysregulation in *fem*CSDS

**FIGURE 6:**
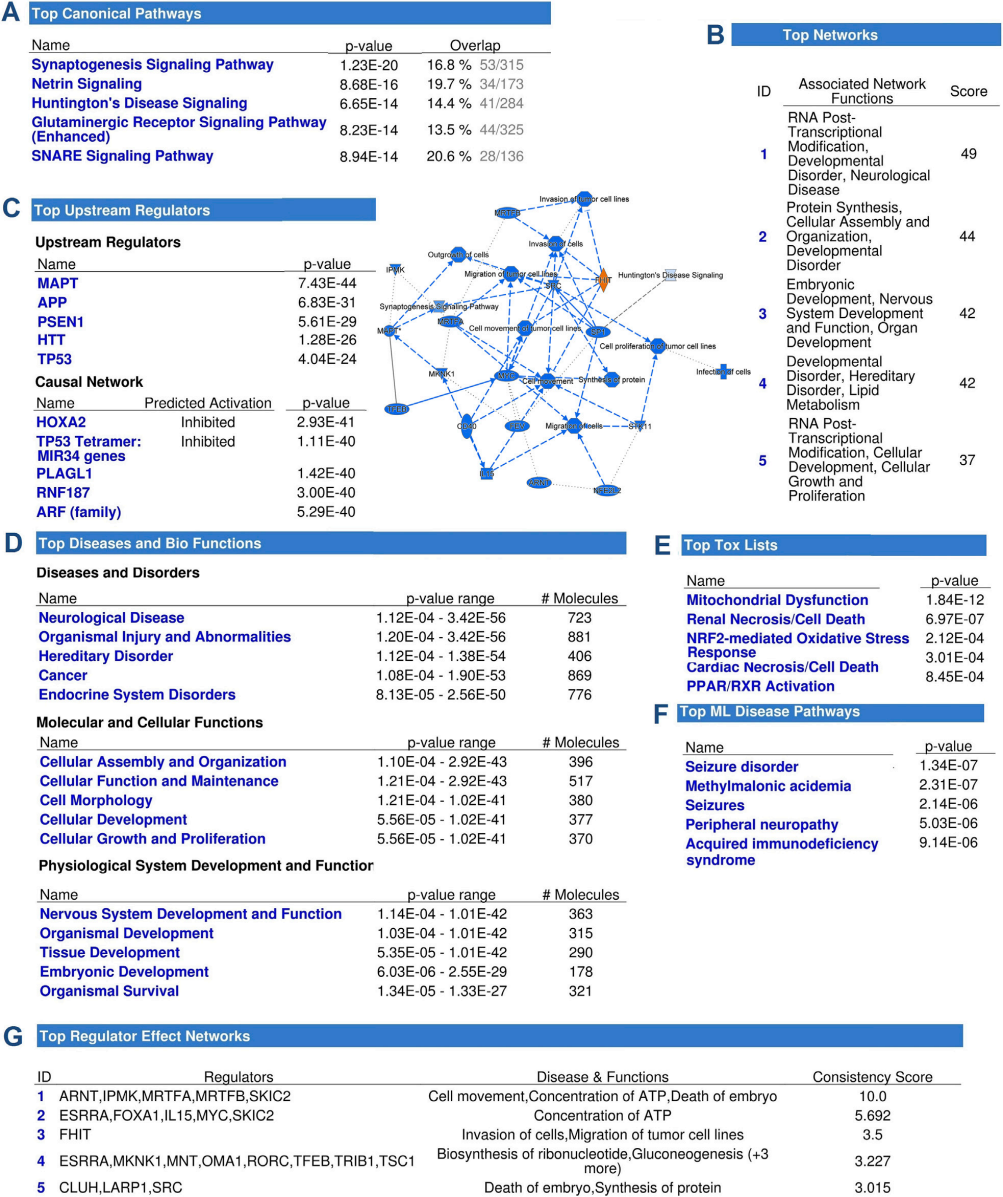
Summary of Ingenuity Pathway Analysis. **(A)** Top canonical pathways enriched among dysregulated proteins. **(B)** Top IPA-generated interaction networks associated with the dysregulated proteome. The highest-scoring networks were related to RNA post-transcriptional modification, protein synthesis, cellular assembly and organization, nervous system development developmental disorders and metabolic regulation. **(C)** Top upstream regulators and causal networks predicted by IPA: Mapt, App, Psen1, Htt and Tp53 were identified as the most significant upstream regulators. Causal network analysis further predicted inhibition of Hoxa2 and Tp53-associated regulatory programs. **(D)** Top disease and biofunction categories associated with *fem*CSDS-induced protein changes. **(E)** Top toxicity-associated pathways identified by IPA. Mitochondrial dysfunction represented the most significantly enriched toxicological pathway, followed by Nrf2-mediated oxidative stress response. **(F)** Top disease-related pathways predicted by IPA. **(G)** Top regulator-effect networks linking upstream regulators, downstream targets and biological functions. The highest-scoring network centered on Arnt, Ipmk, Mrtfa, Mrtfb and Skic2.

To further investigate the functional consequences of the proteomic alterations induced by *fem*CSDS, differentially expressed proteins were subjected to Ingenuity Pathway Analysis (IPA). Canonical pathway enrichment, disease and biofunction analysis, toxicity profiling, upstream regulator prediction and regulator-effect network analyses were performed to identify higher-order biological processes associated with the stress-responsive proteome. A summary of IPA analysis is presented in Figure 6. IPA revealed significant enrichment of pathways related to synaptic organization, neurotransmission, neuronal development, intracellular trafficking, mitochondrial function and cellular metabolism, indicating coordinated alterations across multiple molecular systems within the NAc. Consistent with the STRING and Metascape analyses, synaptic signaling and bioenergetic pathways (Figure 6) emerged as the key biological pathways, suggesting that chronic social defeat stress affects both neuronal communication and cellular energetic homeostasis. List of the top 100 enriched canonical pathways identified by IPA is provided in Supplementary Table 1.

#### 3.7.1 Canonical pathway analysis identified extensive remodeling of synaptic and metabolic signaling networks

Among the most significantly enriched pathways were Synaptogenesis Signaling, Netrin Signaling, Glutamatergic Receptor Signaling, SNARE Signaling, Huntington’s Disease Signaling, Mitochondrial Dysfunction, Oxidative Phosphorylation and TCA Cycle-related pathways. These enrichments indicate that *fem*CSDS associated proteomic alterations extend across multiple systems involved in neuronal communication, intracellular transport, and energy metabolism. The hierarchical disruptions and their relative impact on biological processes are visualized in Supplementary Figure 8.

The Synaptogenesis Signaling pathway represented the most significantly enriched canonical pathway and contained proteins involved in synaptic structure, neurotransmitter release, receptor signaling and intracellular signaling cascades (Supplementary Figure 9). Several proteins associated with presynaptic function and vesicle release, including SNAP25, SYN1 and SYN2, exhibited reduced expression, whereas proteins involved in membrane trafficking and synaptic organization such as STX1B, VPS18, NRXN1, AP2M1 and ITSN1 were increased. Additional alterations were observed in signaling molecules including MTOR, PRKCE, CAMK2D, RAP1B and MAPT, together with increased expression of AKT1 and AKT3 (Table 1). These observations suggest extensive remodeling of proteins involved in synaptic organization and intracellular signaling. In addition, the Glutaminergic Receptor Signaling Pathway (Enhanced) emerged as one of the most significantly enriched canonical pathways (Supplementary Figure 10). Netrin Signaling was also significantly enriched and included marked reductions in the guidance receptors DCC and UNC5C, together with alterations in downstream cytoskeletal and signaling components. Similarly, SNARE Signaling exhibited dysregulation of proteins involved in vesicle docking and neurotransmitter release, further supporting alterations in synaptic vesicle dynamics and membrane trafficking processes.

**TABLE 1:** Key molecules in the synaptogenesis signaling pathway with their expression log ratios identified through IPA analysis.

Symbol	Entrez gene name	Expr log ratio	Expected	Location	Type(s)
CACNB1	Calcium voltage-gated channel auxiliary subunit beta 1	−3.658	​	Plasma membrane	Ion channel
TLN1	Talin 1	−3.462	Up	Plasma membrane	Other
MTOR	Mechanistic target of rapamycin kinase	−2.785	Up	Nucleus	Kinase
SGTA	Small glutamine rich tetratricopeptide repeat co-chaperone alpha	−1.987	​	Cytoplasm	Other
GRM2	Glutamate metabotropic receptor 2	−1.888	Up	Plasma membrane	G-protein coupled receptor
SYT1	Synaptotagmin 1	−1.793	Up	Cytoplasm	Transporter
CADM1	Cell adhesion molecule 1	−1.793	Up	Plasma membrane	Other
SNAP25	Synaptosome associated protein 25	−1.709	Up	Plasma membrane	Transporter
MAPT	Microtubule associated protein tau	−1.447	Up	Plasma membrane	Other
NLGN2	Neuroligin 2	−1.446	Up	Plasma membrane	Enzyme
PRKCE	Protein kinase C epsilon	−1.308	Up	Cytoplasm	Kinase
CPLX1	Complexin 1	−0.995	Up	Plasma membrane	Transporter
SYN2	Synapsin II	−0.897	Up	Plasma membrane	Other
RAP1B	RAP1B, member of RAS oncogene family	−0.882	Up	Cytoplasm	Enzyme
SYN1	Synapsin I	−0.849	Up	Plasma membrane	Other
CNTNAP1	Contactin associated protein 1	−0.807	Up	Plasma membrane	Other
ARPC3	Actin related protein 23 complex subunit 3	−0.794	Up	Cytoplasm	Other
STX16	Syntaxin 16	−0.659	Up	Cytoplasm	Transporter
PRKACB	Protein kinase cAMP-activated catalytic subunit beta	−0.648	Up	Cytoplasm	Kinase
CNTNAP2	Contactin associated protein 2	−0.646	​	Plasma membrane	Other
PAK1	p21 (RAC1) activated kinase 1	−0.629	Up	Cytoplasm	Kinase
RRAS2	RAS related 2	−0.594	Up	Plasma membrane	Enzyme
PRKAR1B	Protein kinase cAMP-dependent type I regulatory subunit beta	−0.589	Up	Cytoplasm	Kinase
RAP2A	RAP2A, member of RAS oncogene family	−0.584	Up	Plasma membrane	Enzyme
RAB3A	RAB3A, member RAS oncogene family	−0.547	Up	Cytoplasm	Enzyme
CAMK2D	Calciumcalmodulin dependent protein kinase II delta	−0.534	Up	Cytoplasm	Kinase
KRAS	KRAS proto-oncogene, GTPase	−0.511	Up	Cytoplasm	Enzyme
YKT6	YKT6 v-SNARE homolog	−0.508	Up	Cytoplasm	Enzyme
GUCY1B1	Guanylate cyclase 1 soluble subunit beta 1	0.567	​	Cytoplasm	Enzyme
ITSN1	Intersectin 1	0.576	Up	Cytoplasm	Other
AP2M1	Adaptor related protein complex 2 subunit mu 1	0.599	Up	Cytoplasm	Other
NRXN1	Neurexin 1	0.643	Up	Plasma membrane	Transmembrane receptor
ITPR1	Inositol 1,4,5-trisphosphate receptor type 1	0.644	Up	Cytoplasm	Ion channel
TIAM1	TIAM Rac1 associated GEF 1	0.644	Up	Cytoplasm	Other
SYT5	Synaptotagmin 5	0.684	Up	Cytoplasm	Transporter
DNAJC5	DnaJ heat shock protein family (Hsp40) member C5	0.699	​	Plasma membrane	Other
PIK3R4	Phosphoinositide-3-kinase regulatory subunit 4	0.737	Up	Cytoplasm	Kinase
EPHA4	EPH receptor A4	0.785	Up	Plasma membrane	Kinase
BRAF	B-raf proto-oncogene, serinethreonine kinase	0.871	Up	Cytoplasm	Kinase
LRP1	LDL receptor related protein 1	0.894	Up	Plasma membrane	Transmembrane receptor
GRIA2	Glutamate ionotropic receptor AMPA type subunit 2	0.905	Up	Plasma membrane	Ion channel
AFDN	Afadin, adherens junction formation factor	0.907	Up	Nucleus	Other
ARHGEF7	Rho guanine nucleotide exchange factor 7	0.965	Up	Cytoplasm	Other
CDH2	Cadherin 2	1.039	Up	Plasma membrane	Other
AKT1	AKT serinethreonine kinase 1	1.058	Up	Cytoplasm	Kinase
CACNA2D2	Calcium voltage-gated channel auxiliary subunit alpha2delta 2	1.109	​	Plasma membrane	Ion channel
ADCY5	Adenylate cyclase 5	1.22	Up	Plasma membrane	Enzyme
NSF	N-ethylmaleimide sensitive factor, vesicle fusing ATPase	1.229	Down	Cytoplasm	Transporter
AKT3	AKT serinethreonine kinase 3	1.254	Up	Cytoplasm	Kinase
GOSR2	Golgi SNAP receptor complex member 2	1.402	Up	Cytoplasm	Transporter
STXBP2	Syntaxin binding protein 2	1.515	Down	Plasma membrane	Transporter
GRIA4	Glutamate ionotropic receptor AMPA type subunit 4	2.008	Up	Plasma membrane	Ion channel
STX1B	Syntaxin 1B	2.838	Up	Plasma membrane	Other

**FIGURE 7:**
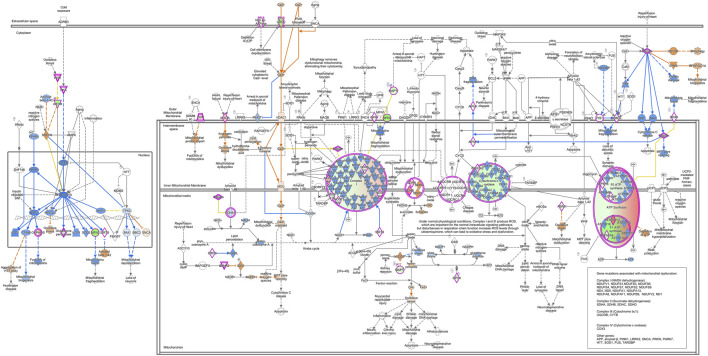
IPA-generated Mitochondrial Dysfunction pathway. Differentially expressed proteins were overlaid onto the Ingenuity Pathway Analysis (IPA) Mitochondrial Dysfunction canonical pathway. Dysregulated proteins are mapped onto multiple components of the mitochondrial respiratory chain, ATP synthase complex, oxidative stress response pathways, mitochondrial dynamics, mitophagy and neurodegeneration-associated signaling cascades. A prominent cluster of downregulated proteins was observed across electron transport chain Complex I subunits (NDUF family members), Complex II components (SDHA, SDHB), Complex III proteins (UQCRFS1), and ATP synthase subunits (ATP5 family), indicating widespread impairment of mitochondrial bioenergetic function.

Several pathways associated with cellular energetics were also significantly enriched. Mitochondrial Dysfunction, Oxidative Phosphorylation and TCA Cycle-related pathways were represented by multiple dysregulated mitochondrial proteins, consistent with the mitochondrial signatures identified by STRING network analysis, hub prioritization, mitochondrial protein profiling and Metascape analyses. Detailed mapping of the Mitochondrial Dysfunction canonical pathway revealed extensive representation of differentially expressed proteins across multiple components of the mitochondrial respiratory machinery (Table 2), including Complex I (NDUFA6, NDUFA9, NDUFA10, NDUFV1), Complex II (SDHA, SDHB), Complex III (UQCRFS1, UQCRQ), Complex IV (NDUFA4), ATP synthase subunits (ATP5F1B, ATP5F1C, ATP5F1E, ATP5MF, ATP5PB) and regulators of mitochondrial dynamics and quality control such as MFN2, OPA1, FIS1, DNM1L and AIFM1 (Figure 7). These alterations were distributed across multiple mitochondrial processes, including oxidative phosphorylation, ATP synthesis, mitochondrial fusion-fission dynamics, oxidative stress regulation and apoptosis-associated signaling. The Glutamatergic Receptor Signaling pathway was characterized by alterations in proteins regulating glutamate transport, receptor signaling and neurotransmission (Supplementary Figure 11). Reduced expression was observed for the glutamate transporter SLC1A3, the metabotropic glutamate receptor GRM2, glutaminase (GLS) and multiple G-protein signaling components including GNG7, GNG12 and GNB5. In contrast, the AMPA receptor subunits GRIA2 and GRIA4, together with the vesicular glutamate transporter SLC17A7, displayed increased expression (Supplementary Table 2). The simultaneous presence of both positively and negatively regulated pathway members indicates substantial reorganization of glutamatergic signaling networks rather than uniform pathway activation or suppression.

**TABLE 2:** Molecules mapped to the IPA mitochondrial dysfunction canonical pathway and their expression changes following *fem*CSDS.

Symbol	Entrez gene name	Expr log ratio	Expected	Location	Type(s)
MFN2	Mitofusin 2	−3.838	Down	Cytoplasm	Enzyme
CACNB1	Calcium voltage-gated channel auxiliary subunit beta 1	−3.658	Down	Plasma membrane	Ion channel
ATP5F1E	ATP synthase F1 subunit epsilon	−2.967	​	Cytoplasm	​
MTOR	Mechanistic target of rapamycin kinase	−2.785	Up	Nucleus	Kinase
DLAT	Dihydrolipoamide S-acetyltransferase	−1.686	Down	Cytoplasm	Enzyme
CAPN5	Calpain 5	−1.596	Up	Cytoplasm	Peptidase
MAPT	Microtubule associated protein tau	−1.447	Up	Plasma membrane	Other
ATP1B1	ATPase Na+K+ transporting subunit beta 1	−1.37	Up	Plasma membrane	​
CAPNS1	Calpain small subunit 1	−1.309	Up	Cytoplasm	Peptidase
COX7A2	Cytochrome c oxidase subunit 7A2	−1.277	Down	Cytoplasm	Enzyme
GPX4	Glutathione peroxidase 4	−1.247	Down	Cytoplasm	Enzyme
HSD17B10	Hydroxysteroid 17-beta dehydrogenase 10	−1.069	Down	Cytoplasm	Enzyme
DNM1L	Dynamin 1 like	−1.063	Up	Cytoplasm	Enzyme
PRDX6	Peroxiredoxin 6	−0.972	Down	Cytoplasm	Enzyme
UQCRFS1	Ubiquinol-cytochrome c reductase, rieske iron-sulfur polypeptide 1	−0.843	Down	Cytoplasm	Enzyme
NDUFA4	NDUFA4 mitochondrial complex associated	−0.835	Down	Cytoplasm	Enzyme
OPA1	OPA1 mitochondrial dynamin like GTPase	−0.738	Down	Cytoplasm	Enzyme
NDUFA9	NADH:ubiquinone oxidoreductase subunit A9	−0.732	Down	Cytoplasm	Enzyme
GSTK1	Glutathione S-transferase kappa 1	−0.654	Down	Cytoplasm	Enzyme
PRKACB	Protein kinase cAMP-activated catalytic subunit beta	−0.648	Down	Cytoplasm	Kinase
ATP5MF	ATP synthase membrane subunit f	−0.647	Down	Cytoplasm	​
NDUFA10	NADH:ubiquinone oxidoreductase subunit A10	−0.646	Down	Cytoplasm	​
ATP5PB	ATP synthase peripheral stalk-membrane subunit b	−0.632	Down	Cytoplasm	​
AIFM1	Apoptosis inducing factor mitochondria associated 1	−0.592	Up	Cytoplasm	Enzyme
PRKAR1B	Protein kinase cAMP-dependent type I regulatory subunit beta	−0.589	Down	Cytoplasm	Kinase
ATP5F1B	ATP synthase F1 subunit beta	−0.559	Down	Cytoplasm	​
CAMK2D	Calciumcalmodulin dependent protein kinase II delta	−0.534	Up	Cytoplasm	Kinase
FIS1	Fission, mitochondrial 1	−0.522	Up	Cytoplasm	Other
NDUFV1	NADH:ubiquinone oxidoreductase core subunit V1	−0.521	Down	Cytoplasm	Enzyme
CACNA2D3	Calcium voltage-gated channel auxiliary subunit alpha2delta 3	−0.517	Down	Plasma membrane	Ion channel
SIRT3	Sirtuin 3	−0.507	Down	Cytoplasm	Enzyme
ATP1A2	ATPase Na+K+ transporting subunit alpha 2	0.538	Up	Plasma membrane	Transporter
TOMM34	Translocase of outer mitochondrial membrane 34	0.582	Down	Cytoplasm	Other
ITPR1	Inositol 1,4,5-trisphosphate receptor type 1	0.644	Down	Cytoplasm	​
PIK3R4	Phosphoinositide-3-kinase regulatory subunit 4	0.737	Up	Cytoplasm	Kinase
SDHA	Succinate dehydrogenase complex flavoprotein subunit A	0.787	Down	Cytoplasm	Enzyme
CACNG8	Calcium voltage-gated channel auxiliary subunit gamma 8	0.839	Down	Plasma membrane	​
MAOA	Monoamine oxidase A	0.878	Up	Cytoplasm	Enzyme
UQCRQ	Ubiquinol-cytochrome c reductase complex III subunit VII	0.914	Down	Cytoplasm	Enzyme
NDUFA6	NADH:ubiquinone oxidoreductase subunit A6	0.987	Down	Cytoplasm	Enzyme
CACNA2D2	Calcium voltage-gated channel auxiliary subunit alpha2delta 2	1.109	Down	Plasma membrane	Ion channel
SDHB	Succinate dehydrogenase complex iron sulfur subunit B	1.772	Down	Cytoplasm	Enzyme
ATP5F1C	ATP synthase F1 subunit gamma	2.018	Down	Cytoplasm	​

**FIGURE 8:**
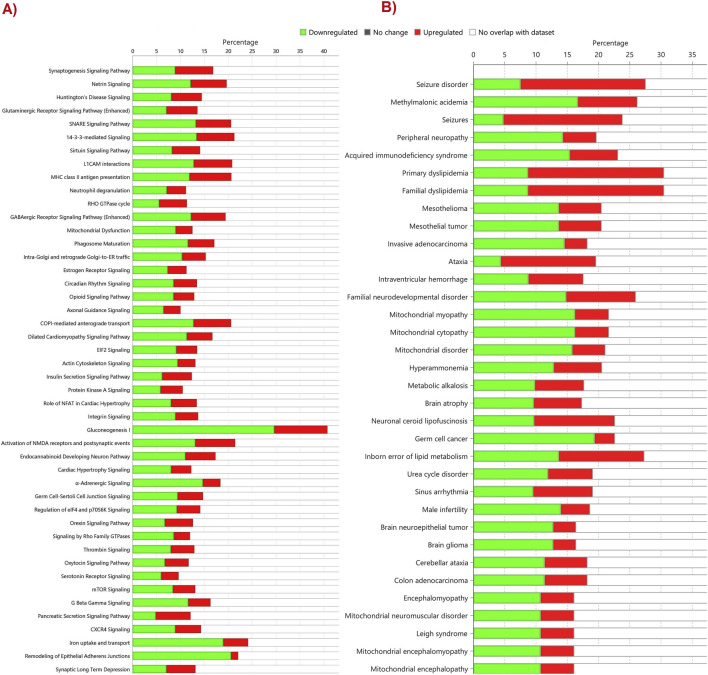
Distribution of dysregulated proteins across enriched canonical pathways and machine learning-derived disease pathways. **(A)** Top enriched canonical pathways: Bars represent the proportion of upregulated (red) and downregulated (green) proteins contributing to each pathway. Major enriched pathways included synaptogenesis signaling, netrin signaling, glutamatergic receptor signaling, SNARE signaling and mitochondrial dysfunction. **(B)** Top machine learning-derived disease pathways: Proteins mapped prominently to neurological, neurodevelopmental and mitochondrial disease signatures, including seizure disorders, peripheral neuropathy, mitochondrial myopathy and mitochondrial cytopathy.

In addition, Estrogen Receptor Signaling emerged as a significantly enriched pathway (Supplementary Figure 12). Multiple components linked to membrane-initiated estrogen signaling and downstream intracellular signaling cascades were altered, including MTOR, PRKCE, GNB5, GNG7, GNG12, AKT1, AKT3 and PLCL2. Given the known influence of estrogen signaling on synaptic plasticity, neuronal metabolism and stress responsiveness, the enrichment of this pathway may be particularly relevant to molecular adaptations occurring within the female NAc following chronic social defeat stress. Detailed list of the major canonical pathways is presented in Figure 8A.

#### 3.7.2 Machine learning disease pathway analysis reveals convergence on neurological, neurodevelopmental and mitochondrial disorders

Machine learning (ML) based disease pathway analysis further linked the *fem*CSDS-associated proteomic alterations to a broad spectrum of neurological, neurodevelopmental and mitochondrial disorders (Figure 8B). Among the highest-ranked disease annotations were seizure disorder, seizures, ataxia, peripheral neuropathy, familial neurodevelopmental disorder, motor dysfunction, progressive neurological disorder, memory deficits, cognitive impairment, and Parkinson’s disease, indicating substantial overlap between the *fem*CSDS proteomic signature and molecular networks implicated in nervous system dysfunction.

Notably, a prominent cluster of enriched disease pathways was related to mitochondrial pathology, including mitochondrial myopathy, mitochondrial cytopathy (Supplementary Figure 13), mitochondrial disorder, mitochondrial encephalopathy, mitochondrial encephalomyopathy, mitochondrial neuromuscular disorder, mitochondrial leukoencephalopathy and mitochondrial DNA-related disorders (Figure 8B). These findings are consistent with the extensive alterations observed in mitochondrial respiratory chain proteins and bioenergetic pathways identified in the proteomic, network and canonical pathway analyses. The ML disease pathway analysis extends the canonical pathway findings by demonstrating that *fem*CSDS associated proteomic alterations map preferentially to neurological and mitochondrial disease networks, suggesting the combined involvement of synaptic remodeling, neuronal signaling and bioenergetic dysfunction.

#### 3.7.3 Disease and functional enrichment analysis

**FIGURE 9:**
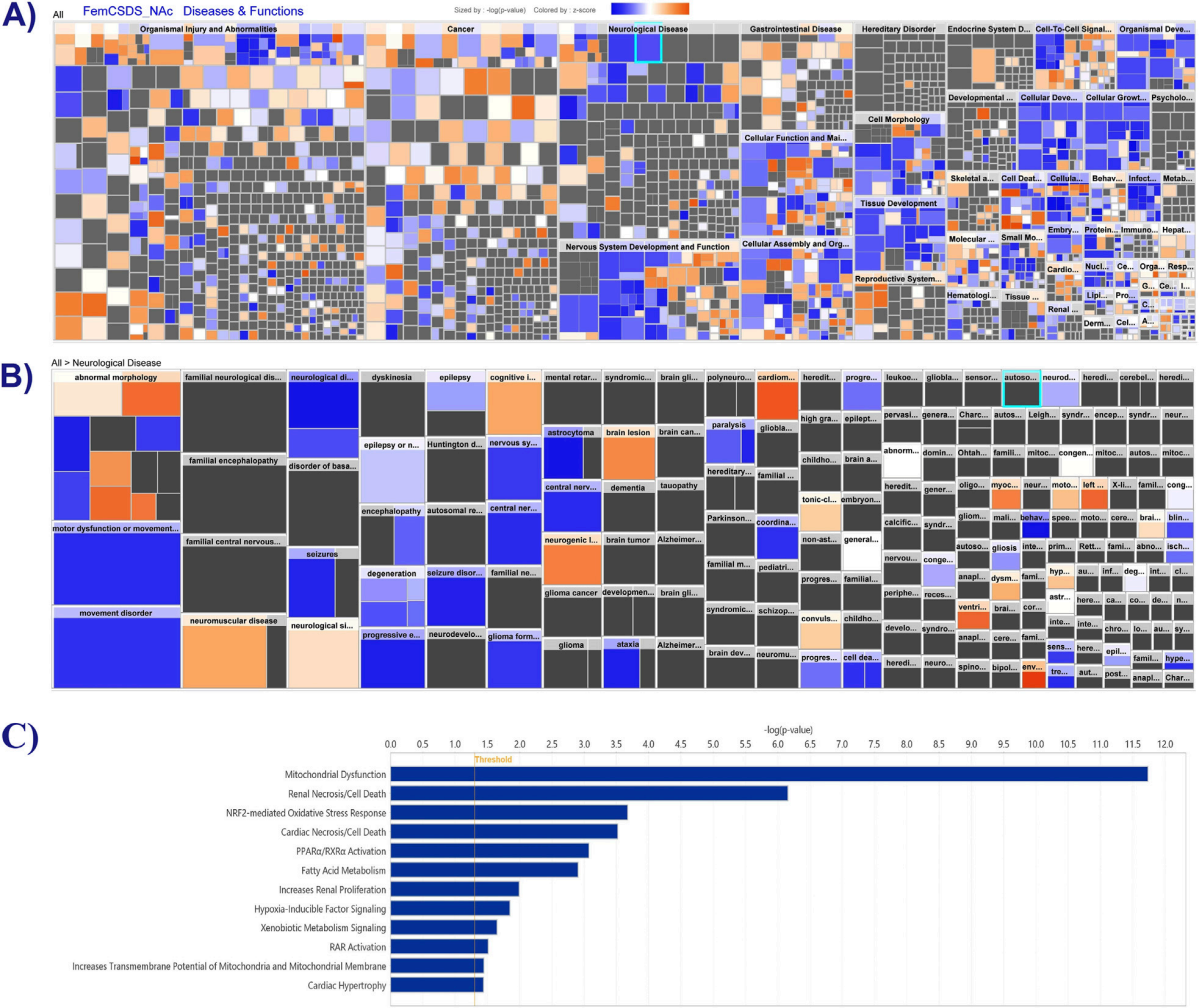
Disease, functional and toxicological landscape of the femCSDS-associated proteome. **(A)** Visualization of significantly enriched disease and functional categories identified by IPA. Major enriched categories included neurological disease, nervous system development and function, cellular assembly and organization, suggestive of widespread alterations in neuronal and cellular regulatory networks. **(B)** Expanded treemap of neurological disease-associated categories. **(C)** Top toxicological pathways enriched among dysregulated proteins: Mitochondrial dysfunction emerged as the most significant toxicity-associated pathway, followed by renal necrosis/cell death, Nrf2-mediated oxidative stress response, cardiac necrosis/cell death, PPARα/RXRα activation, fatty acid metabolism and hypoxia-inducible factor signaling. The predominance of mitochondrial and oxidative stress-related pathways supports impaired bioenergetic homeostasis as a major systems-level consequence of femCSDS within the female NAc.

Disease and biofunction enrichment analysis (Figure 9A) revealed involvement of proteins associated with neurological disease (p = 3.42E-56) (Figure 9B), organismal injury and abnormalities, hereditary disorders, endocrine system disorders and developmental processes (Supplementary Table 3). Among physiological functions, nervous system development and function emerged as one of the most significantly enriched categories, together with cellular assembly and organization, cellular function and maintenance, cell morphology, cellular development, and cellular growth and proliferation. Consistent with the disease and functional enrichment results, IPA toxicity-function analysis further highlighted Mitochondrial Dysfunction as the most significantly enriched toxicity pathway, followed by Nrf2-mediated Oxidative Stress Response, PPARα/RXRα Activation, Fatty Acid Metabolism and Hypoxia-Inducible Factor Signaling (Figure 9C).

#### 3.7.4 Upstream regulatory and functional network analysis identifies synaptic, developmental and bioenergetic regulatory programs

To further identify higher-order regulatory mechanisms underlying the proteomic alterations induced by *fem*CSDS, upstream regulator, causal network and overlapping network analyses were performed using IPA. Integration of the highest-scoring molecular networks revealed extensive connectivity among proteins involved in synaptic transmission, neuronal development, mitochondrial function, protein turnover and cellular stress responses. The merged overlapping network was centered around several highly connected hub molecules, including TP53, SNCA, CUL1/CUL2/CUL3, ATP1A2, ATP1B1, UBE2M, and FUS (Supplementary Figure 14). Upstream regulator analysis identified several molecules linked to neuronal plasticity, metabolism and neurodevelopment. Among these, MTOR (log ratio = −2.785), FMR1 (−0.654), FUS (−0.824), MAPT (−1.447), YAP1 (+1.168) and AKT1 (+1.058) emerged as prominent regulatory nodes with extensive downstream target representation (Supplementary Table 4). These regulators collectively converged on pathways governing synaptic organization, protein synthesis, energy metabolism, autophagy, axonal integrity and neuronal survival.

#### 3.7.5 Regulator effect networks reveal coordinated bioenergetic, translational and neurodevelopmental regulatory programs

To investigate the functional consequences of upstream regulatory changes, IPA regulator effect analysis was performed to integrate upstream regulators, observed targets and downstream biological functions. Several high-confidence regulator networks were identified, linking altered proteins to energy metabolism, protein synthesis, neuronal development, cellular stress responses and cytoskeletal organization (Supplementary Figure 15; Supplementary Table 5). The highest-scoring network was centered on ARNT, IPMK, MRTFA, MRTFB, and SKIC2 (consistency score = 10.0) and was associated with ATP concentration, cell movement and cytoskeletal organization. A second prominent network comprising ESRRA, FOXA1, IL15, MYC, and SKIC2 (consistency score = 5.692) converged on mitochondrial and metabolic proteins including ATP5F1B, ATP5F1E, ATP5PB, ATP5MF, SIRT3, ACLY, AKT1 and LDHA, supporting the broader enrichment of mitochondrial dysfunction observed across pathway analyses (Supplementary Figure 15A).

Additional regulator-effect networks implicated mitochondrial metabolism, nutrient sensing, protein synthesis, and neurodevelopmental processes, including an ESRRA–MKNK1–MNT–OMA1–RORC–TFEB–TRIB1–TSC1 network (Supplementary Figure 15B) and a CLUH–LARP1–SRC network enriched for proteins involved in translational regulation and mitochondrial organization (Supplementary Figure 15C). The FHIT network was associated with migration-related functions and included IPO7, RAB7A, RANBP1 and VIM (Supplementary Figure 15D). In parallel, a strongly inhibited NFE2L2 (NRF2) network linked antioxidant defense pathway with proteins involved in glutathione metabolism, proteostasis and autophagy, including GPX4, GLS, GLUD1, GOT1, UCHL1, ATG7, CUL1, and MAPT (Supplementary Figure 15E). ARNT centered network emerged as a potential integrative node linking mitochondrial dysfunction to neuronal remodeling (Supplementary Figure 15F).

#### 3.7.6 Network analysis of molecular pathways in the CSDS model

The IPA of differentially expressed proteins revealed multiple molecular networks with significant enrichment in neurological and developmental functions. Among the analyzed networks, three primary networks designated as Network 1, Network 2 and Network 3 exhibited high relevance to neurobiological processes, with scores of 49, 44, and 42, respectively. Network 1 molecular composition included proteins involved in mitochondrial function, cytoskeletal organization and metabolic regulation. The prominence of MAPT suggests a potential vulnerability of microtubule structures to chronic stress. Additionally, the involvement of ribosomal proteins and aminoacyl-tRNA synthetases (ARSs) indicates that stress may disrupt protein synthesis fidelity, affecting synaptic plasticity and neuronal function. Network 2 demonstrated a functional enrichment in cellular assembly and developmental disorders. Key molecules in this network include VCPIP1, SGTA and BAG6, which regulate protein quality control and degradation via the ER-associated degradation (ERAD) pathway. A key observation in this network is the presence of GABA-B receptor and Kir3 channel, implicating changes in GABAergic signaling.

### 3.8 SynGO analysis reveals distinct synaptic remodeling and neurotransmission signatures following *fem*CSDS

**FIGURE 10:**
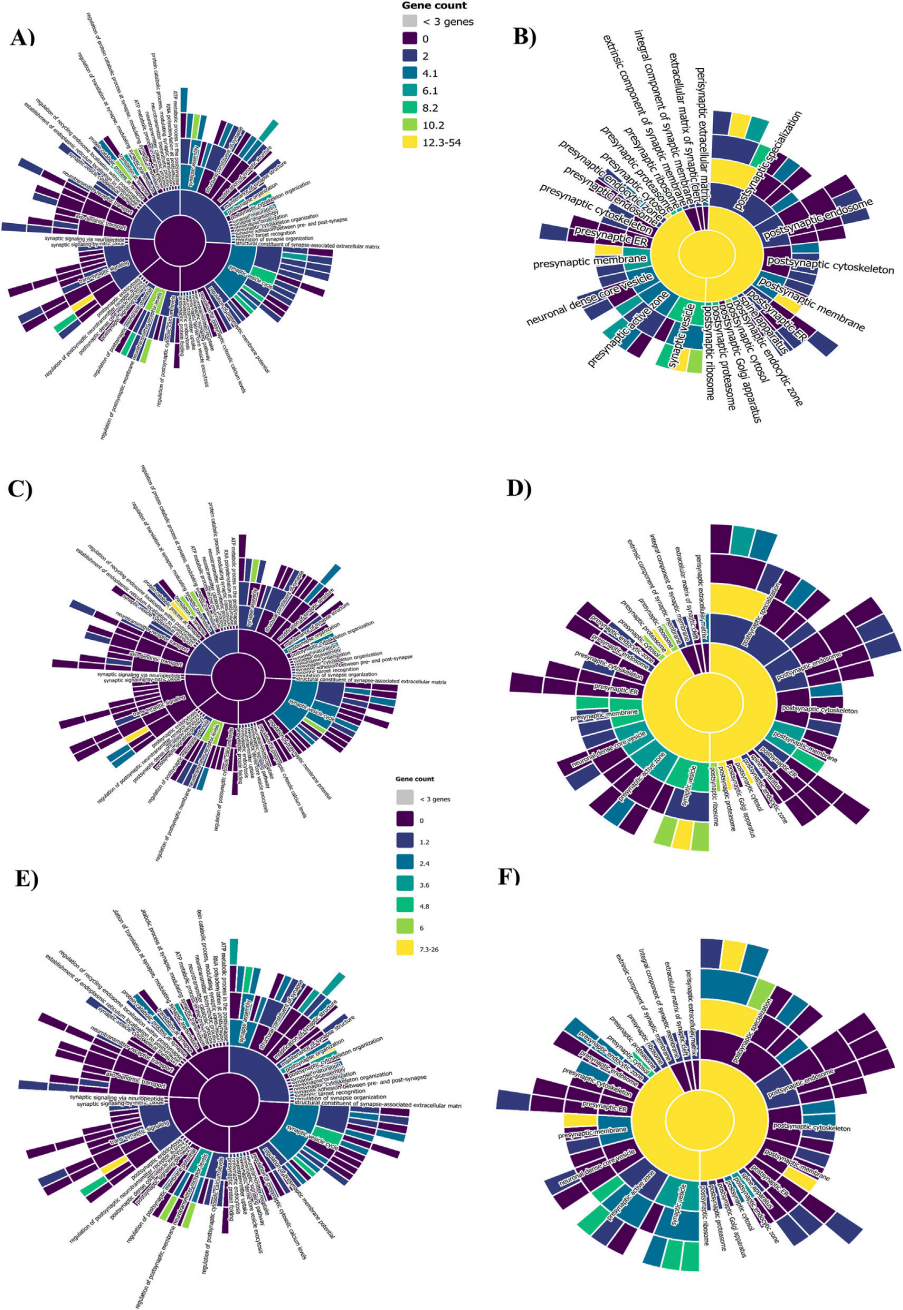
SynGO enrichment analysis. **(A,B)** SynGO enrichment analysis of the complete dysregulated proteome. **(A)** Biological process ontology showing significant enrichment of synaptic signaling, synapse organization, synaptic vesicle cycling and synaptic structural remodeling pathways. **(B)** Cellular component ontology demonstrating enrichment of presynaptic and postsynaptic compartments. **(C,D)** SynGO enrichment analysis of upregulated proteins. **(C)** Upregulated proteins were predominantly associated with synapse organization, postsynaptic specialization, vesicle trafficking and structural plasticity-related processes. **(D)** Corresponding cellular component enrichment showing postsynaptic densities, postsynaptic membranes, postsynaptic cytoskeleton and synaptic vesicle-associated compartments. **(E,F)** SynGO enrichment analysis of downregulated proteins. **(E)** Downregulated proteins were enriched in processes related to synaptic signaling, neurotransmitter receptor transport, synaptic vesicle function, protein catabolic pathways and regulation of postsynaptic receptor levels. **(F)** Cellular component enrichment was concentrated within presynaptic membranes, synaptic vesicles, presynaptic endocytic zones, postsynaptic endosomes and proteasome-associated compartments.

To further characterize synapse-specific alterations induced by *fem*CSDS, SynGO enrichment analysis was performed using the combined, upregulated, and downregulated proteomic datasets. Analysis of the complete dataset identified 276 SynGO-annotated proteins and revealed significant enrichment of 45 biological process and 27 cellular component terms. The strongest enrichments were observed for synapse (258 proteins), process in the synapse (203 proteins), postsynapse (158 proteins), presynapse (141 proteins), synapse organization (74 proteins), postsynaptic specialization (58 proteins), process in the presynapse (57 proteins), postsynaptic density (50 proteins), synaptic vesicle cycle (47 proteins) and process in the postsynapse (43 proteins) (Figures 10A,B).

Separate analysis of upregulated proteins identified 141 SynGO-annotated proteins with significant enrichment of 37 biological process and 26 cellular component terms. The most prominent enrichments included synapse (132 proteins), process in the synapse (113 proteins), postsynapse (86 proteins), presynapse (70 proteins), synapse organization (47 proteins), postsynaptic specialization (39 proteins), process in the presynapse (36 proteins), postsynaptic density (35 proteins), synaptic vesicle cycle (28 proteins) and regulation of postsynaptic membrane neurotransmitter receptor levels (23 proteins) (Figures 10E,F). In contrast, the downregulated dataset contained 135 SynGO-annotated proteins and displayed a more restricted synaptic profile, with 14 biological process and 11 cellular component terms reaching significance. The strongest enrichments included synapse (126 proteins), process in the synapse (90 proteins), postsynapse (72 proteins), presynapse (71 proteins), synapse organization (27 proteins), synaptic vesicle cycle (19 proteins), synaptic vesicle membrane (18 proteins), postsynaptic specialization (19 proteins), postsynaptic density (15 proteins), regulation of postsynaptic membrane neurotransmitter receptor levels (12 proteins), protein catabolic processes at synaptic compartments, synaptic vesicle proton loading, and neurotransmitter receptor transport (Figures 10C,D).

Comparison of the enriched ontologies revealed that several major synaptic categories were represented in both datasets but showed substantially greater enrichment among upregulated proteins. Notably, postsynaptic specialization (39 vs. 19 proteins), postsynaptic density (35 vs. 15 proteins), synapse assembly (15 vs. 8 proteins) and synapse organization (47 vs. 27 proteins) were more prominently represented within the upregulated proteome. In contrast, neurotransmitter receptor transport (4 of 5 mapped proteins) and postsynaptic proteasome-associated proteins (7 of 8 mapped proteins) were largely represented within the downregulated dataset (Table 3).

**TABLE 3:** Comparison of major SynGO-enriched synaptic ontologies identified in combined, upregulated and downregulated proteomic datasets following *fem*CSDS.

SynGO term	Combined (n proteins)	Upregulated (n proteins)	Downregulated (n proteins)
Synapse	258	132	126
Presynapse	141	70	71
Postsynapse	158	86	72
Process in the synapse	203	113	90
Synapse organization	74	47	27
Process in the presynapse	57	36	21
Process in the postsynapse	43	29	14
Postsynaptic specialization	58	39	19
Postsynaptic density	50	35	15
Synaptic vesicle cycle	47	28	19
Regulation of postsynaptic membrane neurotransmitter receptor levels	35	23	12
Chemical synaptic transmission	24	14	10
Synapse assembly	23	15	8
Neurotransmitter receptor transport	5	1	4
Postsynaptic proteasome	8	1	7

### 3.9 ChEA3 transcription factor enrichment identifies ARNT2 and MYT1L as putative master regulators of stress-induced synaptic remodeling

**FIGURE 11:**
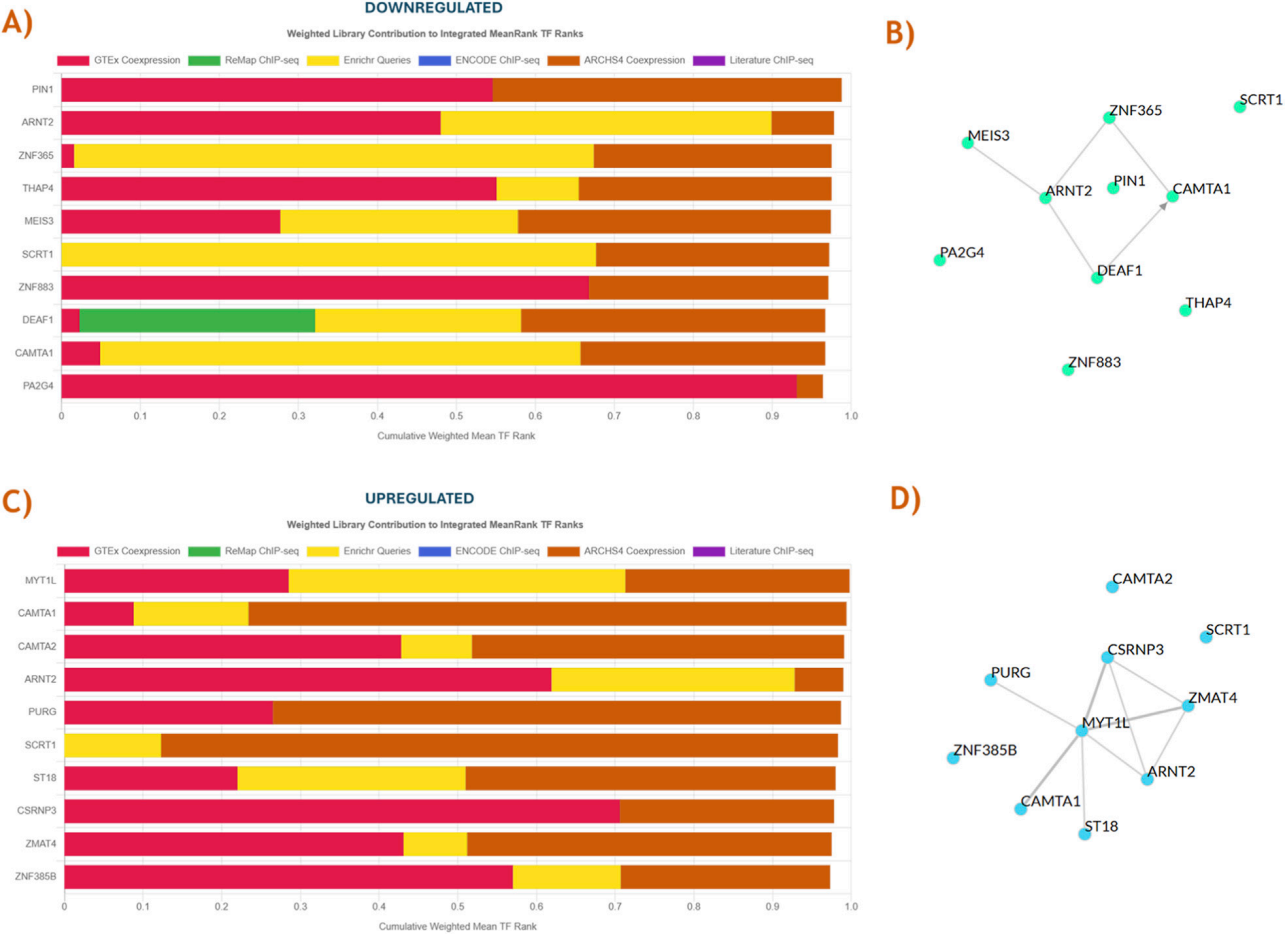
CheA3 transcription factor enrichment identifies distinct regulatory programs associated with downregulated and upregulated proteomic signatures. **(A,B)** Transcription factor analysis of downregulated proteins using the ChEA3 platform. **(A)** Top-ranked transcription factors identified from integrated ChEA3 enrichment scores. PIN1, ARNT2, ZNF365, THAP4, MEIS3 and SCRT1 emerged among the highest-ranked regulatory candidates. **(B)** Regulatory interaction network illustrating relationships among the top enriched transcription factors associated with the downregulated proteome, with ARNT2 occupying a central position within the inferred regulatory architecture. **(C,D)** Transcription factor analysis of upregulated proteins. **(C)** Integrated ChEA3 ranking identified MYT1L as the highest-priority transcriptional regulator, together with CAMTA1, CAMTA2, ARNT2, PURG, SCRT1, CSRNP3, ZMAT4 and ZNF385B. **(D)** Network representation of enriched transcription factors associated with the upregulated proteome, highlighting MYT1L as a central regulatory node interconnected with multiple transcriptional regulators involved in neuronal differentiation, neuronal identity and synaptic function.

To identify transcriptional regulators potentially driving the observed proteomic alterations, ChEA3 transcription factor enrichment analysis was performed using the differentially expressed protein dataset (Figure 11). Among the negatively associated regulatory signatures, PIN1 emerged as the highest-ranked factor, followed by ARNT2, ZNF365, THAP4, MEIS3, SCRT1, DEAF1 and CAMTA1 (Figure 11A). Notably, ARNT2 ranked among the strongest predicted regulators across multiple evidence libraries and exhibited extensive overlap with proteins involved in neuronal differentiation, axonal organization, synaptic signaling, and membrane trafficking, including SNAP25, CNTNAP1, CNTNAP2, DOCK3, SERPINI1, RTN1, STMN3 and CRMP1. Analysis of positively associated regulatory signatures identified MYT1L as the top-ranked transcription factor, followed by CAMTA1, CAMTA2, ARNT2, PURG, SCRT1, ST18 and CSRNP3 (Figure 11B). MYT1L-associated target proteins included several synaptic and neuronal identity proteins, such as GRIA2, NRXN1, RIMS2, PCLO, TRIM9, CYFIP2, SLC6A1 and CLSTN1, indicating enrichment of pathways involved in synapse organization, neurotransmission and neuronal maturation.

## 4 Discussion

The present study provides a comprehensive systems level proteomic characterization of the female NAc following *fem*CSDS and predicts coordinated remodeling of mitochondrial, synaptic, metabolic, translational and proteostatic pathways. Our analysis suggests a model in which chronic social stress drives a metabolically compromised but actively remodeling synaptic state within the female NAc. The NAc is critically involved in reward processing, motivation, salience attribution and stress susceptibility. Previous work has shown that chronic stress-induced depressive phenotypes are strongly associated with altered molecular signaling in the NAc (Fan et al., 2013; Bagot et al., 2015; Karisetty et al., 2024). Importantly, the current findings extend this concept into the female stress-vulnerability context using an ethologically relevant *fem*CSDS model. This is particularly relevant because women exhibit nearly twice the prevalence of depression compared with men, yet most mechanistic stress studies have historically relied on male-centric models (Guo et al., 2004; Moieni et al., 2015; Patel et al., 2025a).

The upregulated network was organized around AP2M1, BIN1, CLTC, DNM1, EPN2, EPN3, GAK and SYNJ1, proteins that collectively govern clathrin-mediated endocytosis, synaptic vesicle recycling and membrane trafficking (Duan et al., 2022). These hubs formed a densely interconnected “synaptic-trafficking circuit”, suggesting that chronic stress preferentially recruits mechanisms involved in vesicle turnover and structural plasticity rather than energy generation. Notably, GRIA2 emerged as the strongest network bottleneck, implying that glutamatergic signaling may function as a critical bridge linking multiple adaptive subnetworks. An additional possibility is that the enrichment of endocytosis and vesicle-trafficking pathways reflects activation of mitochondrial quality-control mechanisms involving extracellular vesicles. Emerging evidence indicates that cells can selectively package damaged mitochondrial proteins, lipids and mitochondrial DNA into mitochondria-derived vesicles (MDVs) or mitochondrial extracellular vesicles (MitoEVs), thereby facilitating mitochondrial quality control, intercellular signaling and disposal of dysfunctional mitochondrial components (Amari and Germain, 2021; Kim et al., 2024).

One of the important proteomic indications of the present analysis was the extensive suppression of mitochondrial and bioenergetic pathways. Downregulated proteins were prominently enriched for oxidative phosphorylation and electron transport chain organization. STRING clustering further demonstrated that many of these proteins formed densely interconnected modules centered around respiratory chain proteins and ATP synthase complexes. Particularly notable was the coordinated downregulation of ATP synthase subunits, complex I-associated proteins and respiratory-chain.

These observations align remarkably well with growing evidence implicating mitochondrial dysfunction as a central mechanism underlying depression (Morris et al., 2020; Casaril et al., 2021; Jiang et al., 2024). Mitochondria are increasingly recognized not merely as energy-producing organelles but as regulators of neuronal plasticity, oxidative stress signaling, apoptosis and inflammatory responses. Importantly, the present dataset also suggests that *fem*CSDS affects mitochondrial quality-control systems in addition to ATP-generating pathways. Several altered proteins identified in our dataset regulate mitochondrial dynamics, including MFN2, OPA1, FIS1 and DNM1L. These proteins are central regulators of mitochondrial fusion, fission, transport and turnover (Cuperfain et al., 2018; Dong et al., 2023).

Interestingly, IPA also predicted inhibition of the NFE2L2 (NRF2) regulatory network. Given the established role of NRF2 in maintaining mitochondrial homeostasis, the concurrent alteration of mitochondrial dynamics proteins is consistent with previous studies linking reduced NRF2 activity to impaired mitochondrial quality control and increased oxidative stress. However, this relationship remains a prediction based on pathway analysis and will require functional validation. Mitochondrial quality-control mechanisms are especially critical in neurons because synaptic transmission imposes exceptionally high energetic demands that require dynamic redistribution of healthy mitochondria throughout neuronal processes. Dysfunction of these adaptive mechanisms may impair local ATP supply, compromise calcium buffering and reduce synaptic resilience under stress conditions (Jiang et al., 2024).

The enrichment of ATP metabolic processes in our analyses may hold particular mechanistic importance. ATP is not simply a metabolic endpoint but a fundamental regulator of synaptic function. Maintenance of membrane potentials, vesicle recycling and cytoskeletal remodeling are all energetically demanding processes that depend heavily on continuous ATP availability. Interestingly, reduced extracellular ATP signaling has previously been linked to depressive-like behavior (Arzola et al., 2022). Similarly, altered ATP bioenergetics have recently been implicated in major depression-associated fatigue (Cullen et al., 2026).

Another major feature of the present study was the simultaneous enrichment of synaptic remodeling pathways despite pronounced mitochondrial suppression. STRING analysis further demonstrated extensive enrichment of proteins involved in membrane trafficking and vesicle dynamics. At first glance, the coexistence of mitochondrial suppression and synaptic enrichment appears paradoxical. However, this apparent contradiction may instead reflect compensatory remodeling (Bansal et al., 2018). Synaptic plasticity is one of the primary adaptive mechanisms by which neural circuits respond to environmental stressors. Rather than indicating enhanced synaptic efficiency, the observed upregulation of structural synaptic pathways may represent an attempt to preserve neuronal communication under conditions of bioenergetic insufficiency.

This interpretation has stronger biological plausibility than a simplistic “synapse increased versus synapse decreased” idea. Additional enrichment was observed for synaptic vesicles, synaptic vesicle membranes, presynaptic active zones, synapse assembly, regulation of postsynaptic neurotransmitter receptor levels and modification of the postsynaptic actin cytoskeleton, indicating widespread involvement of both structural and functional synaptic compartments. These findings suggest that *fem*CSDS is associated with selective reduction of proteins involved in neurotransmission and synaptic maintenance while simultaneously increasing the representation of proteins associated with synaptic organization and structural remodeling. ShinyGO pathway analysis produced concordant results, confirming enrichment of mitochondrial, metabolic and neurodegeneration-related pathways among downregulated proteins and synaptic vesicle cycling, glutamatergic signaling and endocytic pathways among upregulated proteins (Supplementary Figure 16).

The prominent enrichment of glutamatergic signaling-related proteins in the current study is also noteworthy. Alterations in GRIA2, GRIA4, SHANK proteins, DLG2 and receptor-trafficking pathways strongly suggest remodeling of excitatory synapses. Increasing evidence indicates that glutamatergic dysfunction plays a widespread role across neuropsychiatric diseases (McGrath et al., 2022). In depression specifically, dysregulated glutamatergic signaling has emerged as a major mechanistic framework capable of linking stress exposure, inflammation, synaptic dysfunction and impaired neuroplasticity. Interestingly, the observed increase in ribosomal and translational machinery among upregulated proteins may further support the idea of compensatory synaptic adaptation. Proteins involved in translation initiation, ribosomal organization and mitochondrial ribosomal assembly were enriched among upregulated modules. Previous proteomic studies have demonstrated that synaptic remodeling and neurotrophic signaling are accompanied by increased local translational capacity (Liao et al., 2007; Patel et al., 2026). Thus, increased ribosomal enrichment in the present study may reflect increased protein synthesis requirements during stress-associated synaptic restructuring.

**FIGURE 12:**
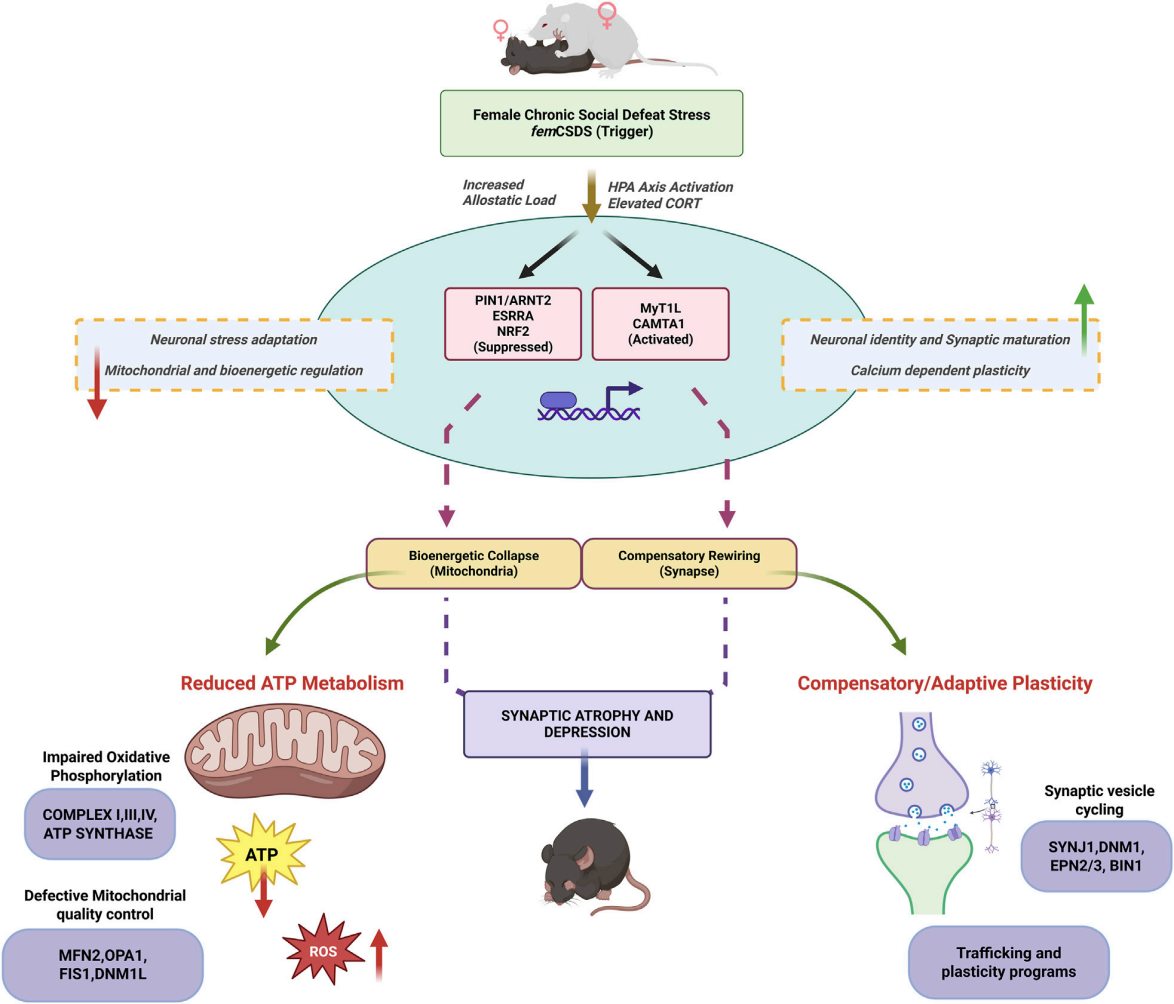
Proposed systems-level mechanism underlying femCSDS-induced molecular remodeling in the female nucleus accumbens. Chronic social defeat stress in females is proposed to induce sustained allostatic load and hypothalamic-pituitary-adrenal (HPA) axis activation, resulting in coordinated transcriptional and proteomic remodeling within the NAc. Systems-level analyses identified suppression of PIN1, ARNT2, ESRRA and NRF2-associated regulatory programs together with activation of MYT1L and CAMTA1 associated transcriptional networks. Downregulated proteins were predominantly enriched in mitochondrial pathways, mitochondrial quality-control machinery and energy-generating metabolic processes, resulting in impaired ATP production and increased oxidative stress. Upregulated proteins were enriched in synaptogenesis, glutamatergic signaling, vesicle trafficking and synaptic remodeling pathways, including SYNJ1, DNM1, EPN2/EPN3 and BIN1, suggesting activation of compensatory synaptic plasticity mechanisms. SynGO, STRING, IPA and Gene Ontology analyses support a model in which mitochondrial dysfunction and disrupted neuronal homeostasis trigger adaptive synaptic rewiring programs. However, these compensatory responses appear insufficient to restore normal network function, ultimately contributing to synaptic dysfunction, NAc circuit remodeling and depression-like behavioral phenotypes following femCSDS.

The current transcription factor enrichment analysis also yielded particularly important findings. Among the top predicted regulators were ARNT2, CAMTA1, THAP4, ZNF365, MEIS3, SCRT1, and ZNF883. ARNT2 was especially notable because it emerged prominently in both combined and directional analyses. Arnt2 has previously been linked to neuronal stress adaptation and neuroprotection (Rahim et al., 2018). Importantly, ARNT2 participates in transcriptional programs associated with neuronal survival and BDNF regulation through interaction with NPAS4. Its enrichment in the current dataset may therefore reflect activation of stress-responsive neuroprotective mechanisms within the female NAc. Similarly, CAMTA family transcription factors identified in our analyses are known regulators of calcium-responsive neuronal transcription and synaptic plasticity. Their enrichment may indicate chronic activation of calcium-dependent adaptive programs in response to stress-induced synaptic instability. We propose a working mechanistic model in which suppression of mitochondrial homeostatic programs and activation of compensatory synaptic plasticity pathways represent parallel but insufficient adaptive responses to chronic social defeat stress in females (Figure 12).

Another important observation from the present study was the convergence between pathways identified in *fem*CSDS and those implicated in neurodegenerative and aging-related disorders. Multiple downregulated proteins identified here are involved in mitochondrial integrity, oxidative stress regulation and protein quality-control systems that are also disrupted in neurodegenerative diseases (Morris et al., 2020; Chen et al., 2025). This overlap supports evidence that chronic stress and depression may accelerate cellular aging-like processes within vulnerable neural circuits. Importantly, the present findings may also help explain why stress-related disorders disproportionately affect females. Estrogen signaling has extensive interactions with mitochondrial regulation, glutamatergic neurotransmission, neuroinflammation and synaptic plasticity (Hwang et al., 2021). Since the female brain exhibits substantial hormonal modulation of stress-responsive pathways, disruptions in these systems may produce distinct molecular vulnerabilities compared with males.

## 5 Limitations

Several limitations of the study must also be acknowledged. The study focused on bulk tissue proteomics and therefore cannot resolve cell-type-specific alterations. Consequently, the observed proteomic changes represent tissue-level molecular alterations and cannot distinguish whether the identified mitochondrial, metabolic, or synaptic signatures originate from neurons, astrocytes, oligodendrocytes, microglia or other components of the neurovascular unit. In addition, although pathway analyses strongly suggest altered synaptic and mitochondrial function, direct physiological validation will be necessary to determine the functional consequences of these molecular changes. Although the estrous cycle was monitored throughout the experimental procedures (Patel et al., 2026), the present proteomic analysis was not stratified according to estrous stage. Therefore, we cannot exclude the possibility that stage-specific hormonal fluctuations contributed to some of the observed molecular differences in the female nucleus accumbens. Furthermore, although the sample size (n = 8 per group) is consistent with previous discovery-based label-free quantitative proteomic studies, validation in larger independent cohorts would further strengthen the generalizability of the findings. Future studies combining single-cell or spatial transcriptomics, cell-type-resolved proteomics, electrophysiology and mitochondrial functional assays will help identify the cellular origin of these proteomic alterations and determine how they influence neural circuit function within the female NAc.

## 6 Conclusions

The present study demonstrates that *fem*CSDS induces large-scale proteomic remodeling within the female NAc characterized by suppression of mitochondrial bioenergetic systems together with activation of synaptic remodeling and structural plasticity pathways. These findings support a model in which chronic social stress produces a bioenergetically compromised yet actively reorganizing neural state. Rather than representing isolated abnormalities the observed changes appear to reflect coordinated network-level adaptations involving mitochondrial dysfunction, impaired proteostasis, altered glutamatergic signaling, receptor trafficking, translational regulation, and compensatory synaptic restructuring. Our findings provide important mechanistic insight into female stress susceptibility and establish a systems-level molecular approach for understanding how chronic social stress reshapes reward circuitry in depression.

## Data Availability

The original contributions presented in the study are publicly available. This data can be found here: https://www.ebi.ac.uk/pride/archive/projects/PXD074507.

## References

[B1] Amari L. Germain M. (2021). Mitochondrial extracellular vesicles – origins and roles. Front. Mol. Neurosci. 14, 767219. 10.3389/fnmol.2021.767219 34751216 PMC8572053

[B2] Arzola E. Xiong W. C. Mei L. (2022). Stress reduces extracellular ATP in the prefrontal cortex and activates the prefrontal cortex–lateral habenula pathway for depressive-like behavior. Biol. Psychiatry 92, 172–174. 10.1016/j.biopsych.2022.05.016 35835503

[B3] Bagot R. C. Parise E. M. Peña C. J. Zhang H. X. Maze I. Chaudhury D. (2015). Ventral hippocampal afferents to the nucleus accumbens regulate susceptibility to depression. Nat. Commun. 6, 7062. 10.1038/ncomms8062 25952660 PMC4430111

[B4] Bansal R. Hellerstein D. J. Peterson B. S. (2018). Evidence for neuroplastic compensation in the cerebral cortex of persons with depressive illness. Mol. Psychiatry 23, 375–383. 10.1038/mp.2017.34 28265119 PMC5589468

[B5] Casaril A. M. Dantzer R. Bas-Orth C. (2021). Neuronal mitochondrial dysfunction and bioenergetic failure in inflammation-associated depression. Front. Neurosci. 15, 725547. 10.3389/fnins.2021.725547 34790089 PMC8592286

[B39] Chen Z. Tang X. Gu C. Zou S. (2025). Investigating biomarkers of mitochondrial and aging-related genes in major depressive disorder through bioinformatics analysis. Front. Psychiatry 16. 10.3389/fpsyt.2025.1653998 41069939 PMC12504309

[B6] Cullen K. R. Tye S. J. Klimes-Dougan B. Wiesner H. M. Varela R. B. Morath B. (2026). ATP bioenergetics and fatigue in young adults with and without major depression. Transl. Psychiatry 16, 158. 10.1038/s41398-026-03904-y 41776167 PMC13004833

[B7] Cuperfain A. B. Zhang Z. L. Kennedy J. L. Gonçalves V. F. (2018). The complex interaction of mitochondrial genetics and mitochondrial pathways in psychiatric disease. Complex Psychiatry 4, 52–69. 10.1159/000488031 29998118 PMC6032034

[B8] Dong W. T. Long L. H. Deng Q. Liu D. Wang J. L. Wang F. (2023). Mitochondrial fission drives neuronal metabolic burden to promote stress susceptibility in Male mice. Nat. Metab. 5, 2220–2236. 10.1038/s42255-023-00924-6 37985735

[B9] Duan D. Hanson M. Holland D. O. Johnson M. E. (2022). Integrating protein copy numbers with interaction networks to quantify stoichiometry in clathrin-mediated endocytosis. Sci. Rep. 12, 5413. 10.1038/s41598-022-09259-w 35354856 PMC8967901

[B10] Fan X. Li D. Zhang Y. Green T. A. (2013). Differential phosphoproteome regulation of nucleus accumbens in environmentally enriched and isolated rats in response to acute stress. PLoS One 8, e79893. 10.1371/journal.pone.0079893 24278208 PMC3838351

[B11] Ge S. X. Jung D. Jung D. Yao R. (2020). ShinyGO: a graphical gene-set enrichment tool for animals and plants. Bioinformatics 36, 2628–2629. 10.1093/bioinformatics/btz931 31882993 PMC7178415

[B12] Guo M. Wu C. F. Liu W. Yang J. Y. Chen D. (2004). Sex difference in psychological behavior changes induced by long-term social isolation in mice. Prog. Neuropsychopharmacol. Biol. Psychiatry 28, 115–121. 10.1016/j.pnpbp.2003.09.027 14687865

[B13] Harris A. Z. Atsak P. Bretton Z. H. Holt E. S. Alam R. Morton M. P. (2018). A novel method for chronic social defeat stress in female mice. Neuropsychopharmacology 43, 1276–1283. 10.1038/npp.2017.259 29090682 PMC5916350

[B40] Hwang W. J. Lee T. Y. Kim N. S. Kwon J. S. (2021). The role of estrogen receptors and their signaling across psychiatric disorders. Int. J. Mol. Sci. 22. 10.3390/ijms22010373 33396472 PMC7794990

[B14] Jiang M. Wang L. Sheng H. (2024). Mitochondria in depression: the dysfunction of mitochondrial energy metabolism and quality control systems. CNS Neurosci. Ther. 30, e14576. 10.1111/cns.14576 38334212 PMC10853899

[B15] Karisetty B. C. Duscharla D. Vijay V. Patel S. Soren K. Kumar A. (2024). Proteome profile of nucleus accumbens (NAc) uncovers the differential and sex-specific role of CRMP2 in CVMS-Induced mouse model of depression. J. Proteins Proteom. 15, 561–576. 10.1007/s42485-024-00145-9

[B16] Keenan A. B. Torre D. Lachmann A. Leong A. K. Wojciechowicz M. L. Utti V. (2019). ChEA3: transcription factor enrichment analysis by orthogonal omics integration. Nucleic Acids Res. 47, W212–W224. 10.1093/nar/gkz446 31114921 PMC6602523

[B17] Kim Y. D’Acunzo P. Levy E. (2024). Biogenesis and secretion of mitovesicles, small extracellular vesicles of mitochondrial origin at the crossroads between brain health and disease. Curr. Opin. Physiol. 40, 100765. 10.1016/j.cophys.2024.100765 39219665 PMC11364255

[B18] Koopmans F. van Nierop P. Andres-Alonso M. Byrnes A. Cijsouw T. Coba M. P. (2019). SynGO: an evidence-based, expert-curated knowledge base for the synapse. Neuron 103, 217–234.e4. 10.1016/j.neuron.2019.05.002 31171447 PMC6764089

[B19] Krämer A. Green J. Pollard J. Tugendreich S. (2014). Causal analysis approaches in ingenuity pathway analysis. Bioinformatics 30, 523–530. 10.1093/bioinformatics/btt703 24336805 PMC3928520

[B20] Liao L. Pilotte J. Xu T. Wong C. C. L. Edelman G. M. Vanderklish P. (2007). BDNF induces widespread changes in synaptic protein content and up-regulates components of the translation machinery: an analysis using high-throughput proteomics. J. Proteome Res. 6, 1059–1071. 10.1021/pr060358f 17330943

[B21] McGrath T. Baskerville R. Rogero M. Castell L. (2022). Emerging evidence for the widespread role of glutamatergic dysfunction in neuropsychiatric diseases. Nutrients 14, 917. 10.3390/nu14050917 35267893 PMC8912368

[B22] Moieni M. Irwin M. R. Jevtic I. Olmstead R. Breen E. C. Eisenberger N. I. (2015). Sex differences in depressive and socioemotional responses to an inflammatory challenge: implications for sex differences in depression. Neuropsychopharmacology 40, 1709–1716. 10.1038/npp.2015.17 25598426 PMC4915253

[B23] Morris G. Walder K. R. Berk M. Marx W. Walker A. J. Maes M. (2020). The interplay between oxidative stress and bioenergetic failure in neuropsychiatric illnesses: can we explain it and can we treat it? Mol. Biol. Rep. 47, 5587–5620. 10.1007/s11033-020-05590-5 32564227

[B24] Patel S. Kushwaha R. Chakravarty S. (2025a). “Neurobiology of depression-associated disability,” in Gender-Specific Perspectives BT - The Palgrave Encyclopedia of Disability. Editors Bennett G. Goodall E. (Cham: Springer Nature Switzerland), 1–18. 10.1007/978-3-031-40858-8_240-1

[B25] Patel S. Kushwaha R. Holkar A. Dhaygude S. S. Kumar A. Chakravarty S. (2025b). Modeling vicarious social defeat stress in rodents for investigating emotional stress-induced depression. J. Psychiatr. Res. 190, 52–68. 10.1016/j.jpsychires.2025.07.040 40763639

[B26] Patel S. Kushwaha R. Anusha P. V. Arvind A. Dhaygude S. S. Pattnaik S. R. (2026). Characterization of a novel female chronic social defeat stress (femCSDS) model utilizing persistently aggressive CD1 parous females. Neuropsychopharmacology 51, 1432–1441. 10.1038/s41386-026-02359-5 41667637 PMC13291270

[B27] Piccinelli M. Wilkinson G. (2000). Gender differences in depression. Critical review. Br. J. Psychiatry 177, 486–492. 10.1192/bjp.177.6.486 11102321

[B28] Rahim T. Becquart P. Baeva M. E. Quandt J. (2018). Expression of the neuroprotective protein aryl hydrocarbon receptor nuclear translocator 2 correlates with neuronal stress and disability in models of multiple sclerosis. J. Neuroinflammation 15, 270. 10.1186/s12974-018-1290-6 30231889 PMC6145183

[B29] Saxena S. Singh S. K. Lakshmi M. G. M. Meghah V. Bhatti B. Swamy C. V. B. (2012). Proteomic analysis of zebrafish caudal fin regeneration. Mol. and Cell. Proteomics 11, M111.014118. 10.1074/mcp.M111.014118 22278371 PMC3433902

[B30] Schaffner W. Weissmann C. (1973). A rapid, sensitive, and specific method for the determination of protein in dilute solution. Anal. Biochem. 56, 502–514. 10.1016/0003-2697(73)90217-0 4128882

[B31] Szklarczyk D. Kirsch R. Koutrouli M. Nastou K. Mehryary F. Hachilif R. (2023). The STRING database in 2023: protein-protein association networks and functional enrichment analyses for any sequenced genome of interest. Nucleic Acids Res. 51, D638–D646. 10.1093/nar/gkac1000 36370105 PMC9825434

[B32] Takahashi A. Chung J.-R. Zhang S. Zhang H. Grossman Y. Aleyasin H. (2017). Establishment of a repeated social defeat stress model in female mice. Sci. Rep. 7, 12838. 10.1038/s41598-017-12811-8 28993631 PMC5634448

[B33] Trainor B. C. Pride M. C. Landeros R. V. Knoblauch N. W. Takahashi E. Y. Silva A. L. (2011). Sex differences in social interaction behavior following social defeat stress in the monogamous California mouse (Peromyscus californicus). PLoS One 6, e17405. 10.1371/journal.pone.0017405 21364768 PMC3045459

[B34] Williams E. S. Mazei-Robison M. Robison A. J. (2022). Sex differences in major depressive disorder (MDD) and preclinical animal models for the study of depression. Cold Spring Harb. Perspect. Biol. 14, a039198. 10.1101/cshperspect.a039198 34404738 PMC8886985

[B35] Xu Y. Lin Y. Yu M. Zhou K. (2024). The nucleus accumbens in reward and aversion processing: insights and implications. Front. Behav. Neurosci. 18, 1420028. 10.3389/fnbeh.2024.1420028 39184934 PMC11341389

[B36] Yohn C. N. Dieterich A. Bazer A. S. Maita I. Giedraitis M. Samuels B. A. (2019). Chronic non-discriminatory social defeat is an effective chronic stress paradigm for both Male and female mice. Neuropsychopharmacology 44, 2220–2229. 10.1038/s41386-019-0520-7 31493767 PMC6898575

[B37] Yu G. Gao C. H. (2025). Enrichplot: Visualization of Functional Enrichment Result. The Comprehensive R Archive Network. Available online at: https://bioconductor.org/packages/enrichplot .

[B38] Zhou Y. Zhou B. Pache L. Chang M. Khodabakhshi A. H. Tanaseichuk O. (2019). Metascape provides a biologist-oriented resource for the analysis of systems-level datasets. Nat. Commun. 10, 1523. 10.1038/s41467-019-09234-6 30944313 PMC6447622

