# Supplementary material for: Female chronic social defeat stress (femCSDS) maps synaptic, bioenergetic and metabolic proteome remodeling in the nucleus accumbens (NAc)
Source: Front Syst Biol. 2026 Aug 4;6:1904427. doi: 10.3389/fsysb.2026.1904427 (PMC13481197; doi:10.3389/fsysb.2026.1904427)
Supplement: Supplementary file 1 [file DataSheet1.pdf]

SUPPLEMENTARY FILE 1

Female chronic social defeat stress (*fem*CSDS) maps synaptic, bioenergetic and metabolic proteome remodeling in the nucleus accumbens (NAc)

Shashikant Patel[1,3#], Roli Kushwaha[1,3#], Anushka Arvind[2], Anusha PV[2], Arvind Kumar[2,3], Mohammed M Idris[2*], Sumana Chakravarty[1,3*]

[1]Applied Biology, CSIR-Indian Institute of Chemical Technology, Tarnaka, Uppal Road, Hyderabad 500007, India.
[2]CSIR- Centre for Cellular and Molecular Biology, Habsiguda, Uppal Road, Hyderabad 500007, India.
[3]Academy of Scientific and Innovative Research (AcSIR), Ghaziabad- 201002, India.

#These authors contributed equally

* Corresponding Authors:

Dr. Sumana Chakravarty
Sr. Principal Scientist, Department of Applied Biology,
CSIR- Indian Institute of Chemical Technology (IICT),
Tarnaka, Hyderabad- 500007 (Telangana)
Email: sumanachak@csiriict.in ; sumana98@gmail.com
Phone: +91 040 27193155
FAX: 040 27193189

Dr. Mohammed M Idris
Scientist-G, Regeneration and Degeneration Laboratory,
CSIR - Centre for Cellular & Molecular Biology,
Habsiguda, Uppal Road, Hyderabad - 500007, Telangana, India
Telephone: 040-27192770
idris@ccmb.res.in

Sup_Table 1: Top 100 canonical pathways identified by IPA

Ingenuity Canonical Pathways	-log(p-value)	Ratio
Synaptogenesis Signaling Pathway	19.9	0.168
Netrin Signaling	15.1	0.197
Huntington's Disease Signaling	13.2	0.144
Glutaminergic Receptor Signaling Pathway (Enhanced)	13.1	0.135
SNARE Signaling Pathway	13	0.206
14-3-3-mediated Signaling	13	0.213
Sirtuin Signaling Pathway	12.8	0.141
L1CAM interactions	12.3	0.208
MHC class II antigen presentation	12.2	0.206
Neutrophil degranulation	12.1	0.111
RHO GTPase cycle	12	0.113
GABAergic Receptor Signaling Pathway (Enhanced)	12	0.194
Mitochondrial Dysfunction	11.6	0.125
Phagosome Maturation	11	0.171
Intra-Golgi and retrograde Golgi-to-ER traffic	10.8	0.153
Estrogen Receptor Signaling	10.8	0.112
Circadian Rhythm Signaling	10.7	0.134
Opioid Signaling Pathway	10.2	0.129
Axonal Guidance Signaling	9.97	0.1
COPI-mediated anterograde transport	9.95	0.206
Dilated Cardiomyopathy Signaling Pathway	9.63	0.167
EIF2 Signaling	9.36	0.135
Actin Cytoskeleton Signaling	9.33	0.131
Insulin Secretion Signaling Pathway	9.18	0.124
Protein Kinase A Signaling	9.1	0.105
Role of NFAT in Cardiac Hypertrophy	9.01	0.134
Integrin Signaling	8.95	0.137
Gluconeogenesis I	8.93	0.407
Activation of NMDA receptors and postsynaptic events	8.92	0.214
Endocannabinoid Developing Neuron Pathway	8.88	0.173
Cardiac Hypertrophy Signaling	8.59	0.123
α-Adrenergic Signaling	8.58	0.183
Germ Cell-Sertoli Cell Junction Signaling	8.46	0.147
Regulation of eIF4 and p70S6K Signaling	8.39	0.141
Orexin Signaling Pathway	8.38	0.126
Signaling by Rho Family GTPases	8.34	0.12
Thrombin Signaling	8.34	0.129
Oxytocin Signaling Pathway	8.32	0.117
Serotonin Receptor Signaling	8.31	0.0959
mTOR Signaling	8.22	0.131
G Beta Gamma Signaling	8	0.163
Pancreatic Secretion Signaling Pathway	7.96	0.121
CXCR4 Signaling	7.89	0.143
Iron uptake and transport	7.78	0.241
Remodeling of Epithelial Adherens Junctions	7.72	0.221
Synaptic Long Term Depression	7.72	0.131
Sensory processing of sound by inner hair cells of the cochlea	7.63	0.217

Translocation of SLC2A4 (GLUT4) to the plasma membrane	7.45	0.211
Integration of energy metabolism	7.44	0.175
GNRH Signaling	7.42	0.131
The citric acid (TCA) cycle and respiratory electron transport	7.15	0.236
HSP90 chaperone cycle for steroid hormone receptors in the presence of ligand	7.15	0.236
CLEAR Signaling Pathway	7.12	0.109
Synaptic Long Term Potentiation	7.12	0.152
GABA Receptor Signaling	7.12	0.152
Signaling by VEGF	7.11	0.167
P2Y Purinergic Receptor Signaling Pathway	7.06	0.15
Adrenergic Receptor Signaling Pathway (Enhanced)	7.02	0.125
Protein folding	7.02	0.173
Protein Ubiquitination Pathway	7.01	0.11
Antiproliferative Role of Somatostatin Receptor 2	6.95	0.195
Neurotransmitter release cycle	6.87	0.275
Gap Junction Signaling	6.82	0.102
GABA synthesis, release, reuptake and degradation	6.77	0.421
Protein Sorting Signaling Pathway	6.77	0.129
KEAP1-NFE2L2 pathway	6.73	0.176
Insulin Receptor Signaling	6.68	0.143
NIK-->noncanonical NF-kB signaling	6.68	0.217
Gap junction trafficking and regulation	6.6	0.294
Clathrin-mediated endocytosis	6.54	0.146
EPH-Ephrin signaling	6.52	0.17
Glycolysis I	6.52	0.333
Oxytocin in Brain Signaling Pathway	6.47	0.121
Parkinson's Signaling Pathway	6.4	0.101
Neddylation	6.36	0.11
Semaphorin interactions	6.33	0.203
Class I MHC mediated antigen processing and presentation	6.31	0.0933
Eukaryotic Translation Initiation	6.29	0.148
Endocannabinoid Neuronal Synapse Pathway	6.24	0.134
Nuclear Cytoskeleton Signaling Pathway	6.22	0.114
RHOGDI Signaling	6.22	0.114
Calcium Signaling	6.22	0.114
Semaphorin Neuronal Repulsive Signaling Pathway	6.19	0.133
RAC Signaling	6.18	0.139
TP53 Regulates Metabolic Genes	6.16	0.17
Hedgehog 'off' state	6.1	0.15
Tight Junction Signaling	6.09	0.122
IL-8 Signaling	6.04	0.114
Opioid Signalling	6.03	0.167
TCA Cycle II (Eukaryotic)	6.02	0.348
Apelin Endothelial Signaling Pathway	5.99	0.135
Signaling by ROBO receptors	5.94	0.147
Autism Signaling Pathway	5.88	0.0974
Epithelial Adherens Junction Signaling	5.83	0.127
Virus Entry via Endocytic Pathways	5.83	0.144
GPCR-Mediated Nutrient Sensing in Enteroendocrine Cells	5.83	0.144
RAF/MAP kinase cascade	5.82	0.103

Ephrin Receptor Signaling	5.79	0.114

Sup_Table 2: Key molecules in the Glutamatergic Receptor Signaling Pathway with their expression log ratios identified through IPA analysis

Symbol	Entrez Gene Name	GenPept/RefSeq	Expr Log Ratio	Expected	Location	Type(s)
SLC1A3	solute carrier family 1 member 3	AAK01708.1	-2.175		Plasma Membrane	transporter
GRM2	glutamate metabotropic receptor 2	EDL21160.1	-1.888	Up	Plasma Membrane	G-protein coupled receptor
GNG7	G protein subunit gamma 7	XP_036011512.1	-1.424		Plasma Membrane	other
GNG12	G protein subunit gamma 12	CAG9553592.1	-1.272		Plasma Membrane	enzyme
GNB5	G protein subunit beta 5	NP_034443.1	-1.199		Plasma Membrane	other
GLS	glutaminase	D3Z7P3.1	-1.115		Cytoplasm	enzyme
SLC17A7	solute carrier family 17 member 7	BAE34790.1	0.579		Plasma Membrane	transporter
GRIA2	glutamate ionotropic receptor AMPA type subunit 2	AAC37654.1	0.905	Up	Plasma Membrane	ion channel
GRIA4	glutamate ionotropic receptor AMPA type subunit 4	XP_006509905.1	2.008	Up	Plasma Membrane	ion channel

Sup_Table 3: Significantly enriched Diseases and Biofunctions identified in the *fem*CSDS model, reflecting neurological, cellular and metabolic dysregulation.

Diseases or Functions Annotation	p-value	z-score	Representative Molecules
Long-term potentiation of hippocampal cells	8.65E-05	-2.433	Camk4, Cdh2, Fmr1, Gria2, Ptpn5
Depolymerization of actin filaments	6.75E-05	-2.183	Cap1, Cfl2, Dstn, Gsn, Pfn1
Shape change of fibroblast cell lines	6.74E-05	-2.556	Flna, Rhog, Dgkz, Dock3, Vim
Concentration of ATP	4.26E-05	-2.131	Atp5f1b, Aifm1, Sirt3, Dnm1l, Maoa
Behavioral deficit	3.26E-06	-2.309	Cntnap2, Fmr1, Gria2, Mecp2, Shank3
Biosynthesis of ribonucleotide	9.82E-07	-2.041	Mfn2, Opa1, Sdha, Akt1, Atp5f1b
Biosynthesis of macromolecule	2.83E-07	-2.175	Mtor, Grm2, Fmr1, Eif4e, Tp53bp1
Anxiety	1.96E-08	-2.545	Gria4, Grm2, Mecp2, Shank2, Shank3
Long-term potentiation of hippocampus	5.71E-09	-2.006	Camk4, Gria2, Itpr1, Ptk2b, Ptpn5
Progressive neurological disorder	3.85E-26	-2.157	Mapt, Mfn2, Mtor, Gria2, Gria4, Shank2, Slc1a3, Snap25, Sv2a, Mbp
Migration of neurons	2.07E-05	2.294	Epha4, Chl1, Mapt, Ptprz1, Trio
Environmentally induced seizure	1.40E-05	2.449	Cntnap2, Gng7, Syn1, Syn2, Coq8a
Cell movement of neurons	7.18E-06	2.194	Mapt, L1cam, Nrcam, Ptprz1, Trio
Initiation of expression of RNA	2.11E-06	2.000	Eif2s1, Eif3c, Eif4e, Fmr1, Mecp2
Receptor-mediated endocytosis	4.40E-10	2.313	Ap2m1, Cltc, Dnajc6, Lrp1, Synj1
Organization of cells	1.50E-12	2.236	Ank3, Mapt, Mbp, Nefl, Syn1
Associative learning	1.21E-04	—	Mecp2, Shank2, Shank3, Snap25, Slc6a1
Myelination	1.14E-04	0.058	Mbp, Mag, Cntnap1, Cntnap2, Ptprz1

Sup_Table 4. Representative upstream regulators associated with synaptic, developmental, mitochondrial and stress-related pathways in the NAc following femCSDS

Functional Category	Regulator	z-score	p-value
Synaptic plasticity / translational control	Fmr1	-1.315	1.52E-18
RNA metabolism / neurodegeneration	Fus	0.308	1.48E-10
Cytoskeletal stability / tau signaling	Mapt	-2.169	7.43E-44
mTOR signaling / cellular metabolism	Mtor	-0.408	2.78E-08
Autophagy regulation	Atg7	-0.943	1.87E-04
Neurodevelopment / synaptic maturation	Mecp2	-0.827	3.06E-04
Growth signaling	Akt1	-1.345	2.04E-02
Hippo pathway / cellular remodeling	Yap1	-0.801	8.20E-05
Mitochondrial biogenesis / lysosomal function	Tfeb	-3.235	2.98E-03
Hypoxia & metabolic adaptation	Arnt	-2.789	2.07E-04
Neurodegenerative disease-associated regulator	App	1.239	6.83E-31
Neurodegenerative disease-associated regulator	Psen1	-0.306	5.61E-29
Neurodegenerative disease-associated regulator	Htt	-0.261	1.28E-26
Stress-response hub	Tp53	-1.465	4.04E-24

Sup_Table 5: List of Regulator effects

Consistency Score	Node Total	Regulator Total	Regulators	Target Total	Target Molecules in Dataset	Diseases & Functions
10	33	5	ARNT,IPMK,MRTFA,MRTFB,SKIC2	25	Actc1,Actg1,Alcam,Aldoa,Capns1,Cd81,Cdkn1b,Ddah1,Dnajb4,Eif6,Flna,Gapdh,Hsp90ab1,Itgav,Ldha,Lmna,Myh10,Myl9,Myo1c,Neo1,Pfn1,Sorbs1,Tfrc,Tpi1,Vim	Cell movement,Concentration of ATP,Death of embryo
5.692	16	5	ESRRA,FOXA1,IL15,MYC,SKIC2	10	Acly,Akt1,Aldoa,Atp5f1b,Atp5if1,Gapdh,Ldha,Sirt2,Sirt3,Yap1	Concentration of ATP
3.5	7	1	FHIT	4	Ipo7,Rab7a,Ranbp1,Vim	Invasion of cells,Migration of tumor cell lines
3.227	73	8	ESRRA,MKNK1,MNT,OMA1,RORC,TFEB,TRIB1,TSC1	60	Acadvl,Acsl1,Acsl6,Actc1,Alcam,Aldoa,Aldoc,Ap1b1,Asah1,Atp1b1,Atp5f1b,Atp6v0a1,Atp6v0c,Atp6v0d1,Atp6v1a,Cacna2d2,Cacnb1,Cad,Clta,Cltc,Clu,Crmp1,Dnm1l,Flna,Gaa,Gapdh,Gls,Got1,Got2,Hadha,Itpr1,Klc1,Ldha,Ldhb,Mapt,Mdh1,Mdh2,Mfn2,Ndufa4,Nrcam,Nrxn1,Opa1,Pgam1,Pgk1,Ppt1,Rab14,Rab3a,Rab7a,Sdhb,Sirt3,Slc1a3,Snap25,Sparcl1,Syn2,Tagln,Tpi1,Tsc2,Ube2o,Vim,Ywhaz	Biosynthesis of ribonucleotide,Gluconeogenesis,Infection by RNA virus,Macrovesicular hepatic steatosis,Progressive neurological disorder
3.015	16	3	CLUH,LARP1,SRC	11	Cdkn1b,Eef1a1,Eif3h,Fh,Gfm1,Opa1,Rpl30,Rps20,Rps23,Sirpa,Vim	Death of embryo,Synthesis of protein
1.768	11	2	NR6A1,UBA1	8	Camk1,Cdkn1b,Gls,Hspb1,Lmna,Nefl,Tagln,Vim	Apoptosis
1.155	5	1	SP1	3	Flna,Prkce,Vim	Cell spreading of kidney cell lines
0.603	18	6	ADORA2A,CD40,INSR,PALMD,RORC,STK11	11	Aldoc,Gapdh,Got1,Hadha,Mdh1,Mdh2,Mfn2,Pgam1,Pgk1,Pgm1,Tpi1	Gluconeogenesis
0.13	63	1	MAPT	59	Aak1,Acly,Actg1,Asah1,Atp1a2,Atp1b1,Atp4a,Atp5f1b,Atp5pb,Atp6v0d1,Atp6v1e1,Bsg,Bsn,Cap1,Cfl2,Cltc,Dnajc5,Dnajc6,Dnm1l,Dpysl2,Dync1h1,Eno2,Ephx1,Eprs1,Gad1,Gapdh,Gfap,Got2,Hba1/Hba2,Hspb1,Klc1,Ldhb,Lrp1,Mbp,Mdh1,Mog,Mtor,Nme1,Pak1,Pak3,Pclo,Pde2a,Ppt1,Prdx6,Slc1a3,Snap25,Stip1,Suclg1,Syn1,Synj1,Thy1,Tuba1c,Tuba4a,Tubb,Tubb2a,Txn,Uchl1,Vim,Ywhaz	Organization of cells,Progressive neurological disorder,Receptor-mediated endocytosis
-1.923	55	1	MAPT	53	Aak1,Acat1,Acly,Aldoa,Asah1,Atp1a2,Atp5f1b,Bsg,Camk2d,Cdkn1b,Cltc,Cyfip2,Dnajc5,Dnm1l,Dync1h1,Ephx1,Eprs1,Fus,Gapdh,Gfap,Got2,Gpx4,Hba1/Hba2,Hk1,Hspb1,Hsph1,Inpp4a,Lrp1,Mbp,Mdh1,Mog,Mtor,Nme1,Pak1,Pak3,Ppp2ca,Ppt1,Prdx3,Prdx6,	Necrosis

					Ptk2b,Slc1a3,Snap25,Stip1,Stx1b,Thy1,Tln1,Tubb,Txn,Uchl1,Uqcrq,Vim,Ywhae,Ywhaz	
-1.938	47	1	MAPT	45	Acly,Actg1,Add1,Add2,Aldoa,Anxa6,Atp5f1b,Bsg,Camk2d,Cap1,Cdkn1b,Cltc,Dnm1l,Dpysl2,Dync1h1,Eprs1,Fbxo41,Gad1,Gapdh,Gfap,Got2,Gpx4,Gucy1b1,Hk1,Hspb1,Inpp4a,Lrp1,Mapre2,Mog,Mtor,Nme1,Pak1,Pak3,Pde2a,Ptk2b,Septin7,Slc1a3,Snap25,Thy1,Tln1,Tpm1,Txn,Vim,Ywhae,Ywhaz	Migration of cells
-2.846	12	1	RORC	10	Aldoa,Gls,Glud1,Got1,Got2,Ldha,Mdh1,Nsdhl,Pgk1,Ube2o	Cell proliferation of tumor cell lines
-4	11	1	TFEB	9	Ap1b1,Atp6v0a1,Atp6v0c,Atp6v0d1,Atp6v1a,Clta,Cltc,Rab7a,Vim	Infection of cells
-4.491	8	1	STX18	6	Cadm1,Ilf3,Sfpq,Spock1,Ssrp1,Tagln	Apoptosis
-6	6	1	CDKN2B	4	Gls,Glud1,Got1,Got2	Cell proliferation of tumor cell lines
-6.708	7	1	SP1	5	Cdh2,Gnai2,Gria2,Maoa,Mecp2	Behavioral deficit
-10.205	9	1	MKNK1	7	Clu,Crmp1,Mapt,Ncdn,Nrcam,Nrxn1,Vim	Outgrowth of cells
-12.333	12	1	CD40	10	Aimp2,Cdkn1b,Cul1,Gapdh,Gls,Hk1,Ipo4,Ldha,Npm1,Uqcrfs1	Cell death of tumor cell lines
-12.969	7	1	CHI3L1	5	Akt3,Ldha,Nampt,Tppp,Vim	Cell movement of tumor cell lines
-13.5	18	1	SP1	16	Bsg,Cdkn1b,Cplx1,Dlat,Dnm1,Gja1,Got1,Gria2,Itgav,Itpr1,Maoa,Mfn2,Prkce,Ranbp1,Serpine2,Slc25a5	Apoptosis of tumor cell lines
-31.197	31	1	NFE2L2	29	Atg7,Cbr1,Cct3,Cul1,Dctn3,Eif2s1,Eif3c,Eif4g2,Gbe1,Gls,Glud1,Gna11,Got1,Gpx4,Hsp90ab1,Ide,L1cam,Ldha,Lmna,Mapt,Pcdh7,Psmd3,Psmd7,Ranbp1,Sirt3,Stip1,Tp53bp1,Txn,Uchl1	Cell death of tumor cell lines

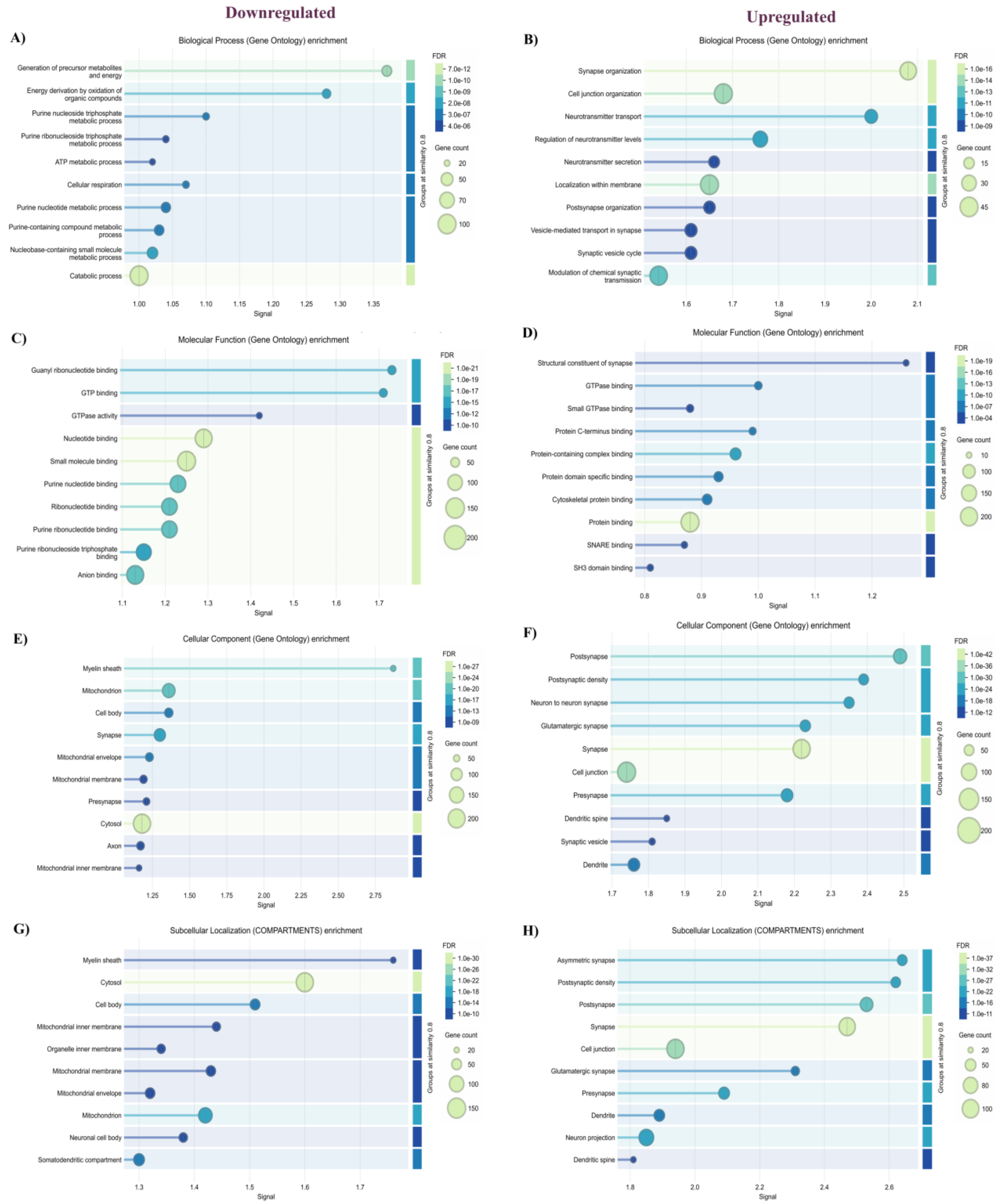

Supplementary Figure 1. STRING functional enrichment analysis of dysregulated protein interactomes following *fem*CSDS: STRING enrichment analysis was performed separately on downregulated and upregulated proteins identified in the nucleus accumbens following *fem*CSDS. Downregulated proteins were predominantly enriched for mitochondrial respiration, oxidative phosphorylation, ATP synthesis, protein translation, ubiquitin-mediated proteostasis and metabolic pathways. In contrast, upregulated proteins showed enrichment for synaptic

organization, clathrin-mediated endocytosis, vesicle trafficking, neurotransmitter release and ribosome-associated processes. Enrichment significance is represented as −log10 adjusted p-values, highlighting the distinct biological programs associated with suppressed and activated proteomic networks. (A) Top enriched Gene Ontology Biological Process (GO-BP) terms among downregulated proteins. (B) Top enriched Gene Ontology Biological Process (GO-BP) terms among upregulated proteins. (C) Top enriched KEGG pathways among downregulated proteins. (D) Top enriched KEGG pathways among upregulated proteins. (E) Top enriched Reactome pathways among downregulated proteins. (F) Top enriched Reactome pathways among upregulated proteins. (G) Top enriched Gene Ontology Molecular Function (GO-MF) terms among downregulated proteins. (H) Top enriched Gene Ontology Molecular Function (GO-MF) terms among upregulated proteins.

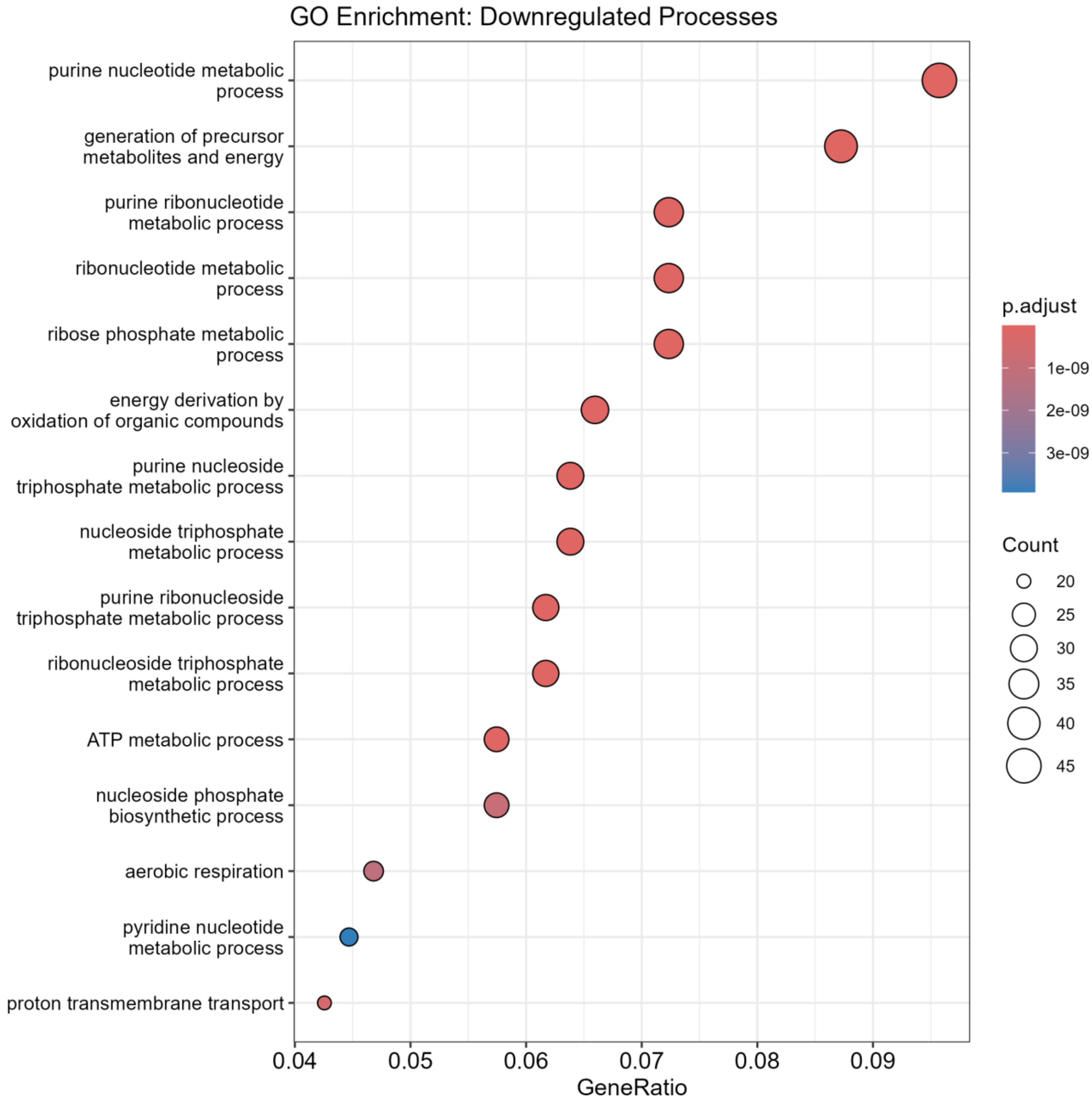

Supplementary Figure 2. Gene Ontology enrichment analysis identifies coordinated suppression of mitochondrial and bioenergetic pathways following *fem*CSDS. Dot plot showing the top significantly enriched Gene Ontology Biological Process terms among proteins downregulated in the nucleus accumbens following female chronic social defeat stress (*fem*CSDS). Enriched processes were predominantly associated with cellular energy metabolism, including purine nucleotide metabolism, ATP metabolic processes, generation of precursor metabolites and energy, aerobic respiration, proton transmembrane transport, oxidation of organic compounds and ribonucleotide biosynthetic pathways. The x-axis represents the GeneRatio, bubble size indicates the number of proteins associated with each term and color denotes the adjusted p-value significance.

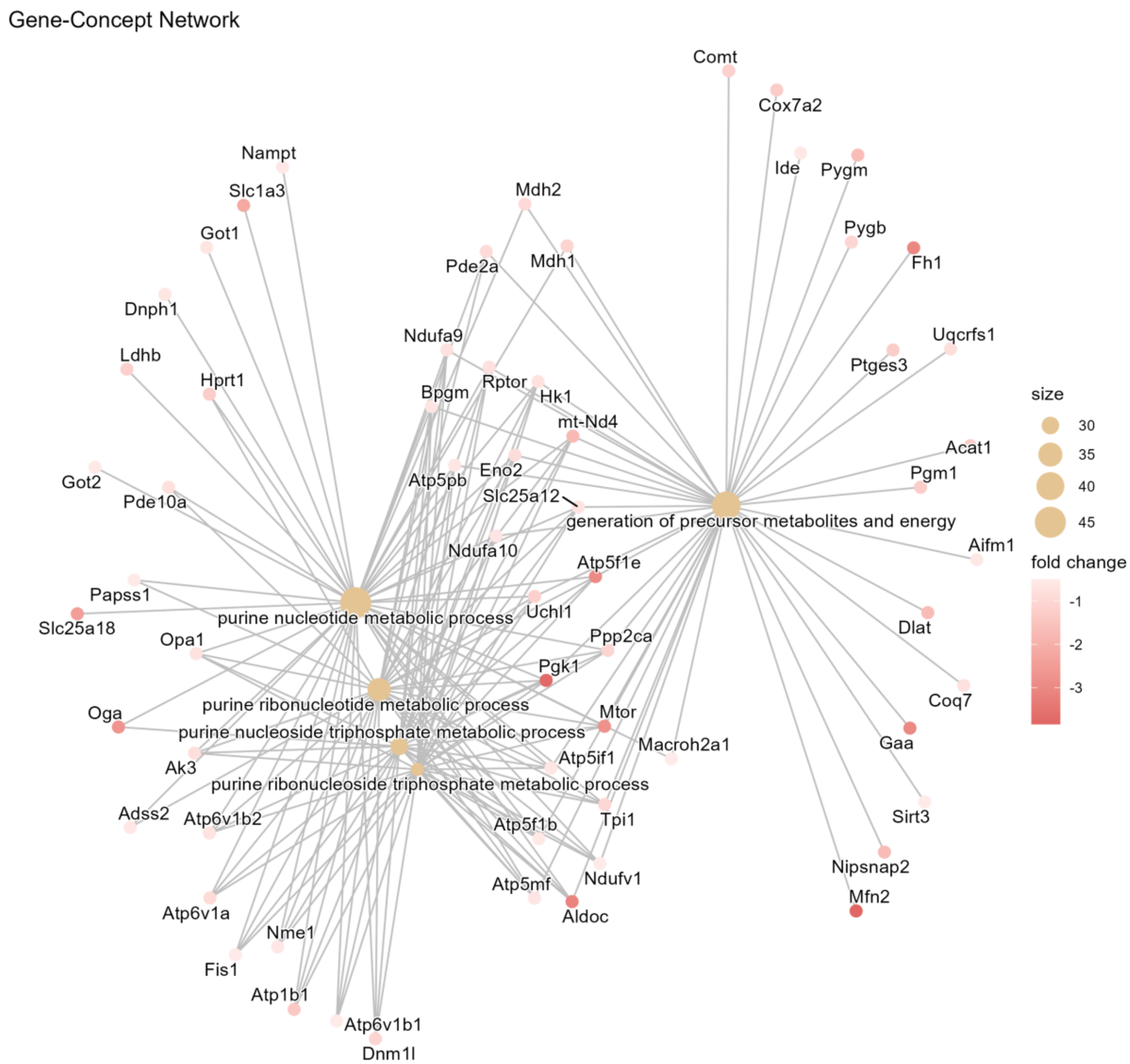

Supplementary Figure 3. Gene–concept network reveals extensive overlap among downregulated bioenergetic and mitochondrial pathways following *fem*CSDS. Gene-concept network illustrating the relationships between significantly enriched Gene Ontology Biological Process terms and their associated downregulated proteins in the nucleus accumbens *fem*CSDS. Larger gold nodes represent enriched biological processes, whereas smaller nodes represent individual proteins. Node size reflects the number of genes associated with each biological process and node color represents protein Log2 fold change. Multiple highly enriched processes, including generation of precursor metabolites and energy, purine nucleotide metabolic process, purine ribonucleotide metabolic process, purine nucleoside triphosphate metabolic process and purine ribonucleoside triphosphate metabolic process, converged on a common set of mitochondrial and metabolic proteins. Shared nodes included ATP synthase subunits (ATP5F1B, ATP5F1F, ATP5MF), vacuolar H^{+}-ATPase subunits (ATP6V1A, ATP6V1B1, ATP6V1B2), electron transport chain components (NDUFA9, NDUFA10, NDUFV1, UQCRFS1, mt-ND4), mitochondrial dynamics regulators (MFN2, OPA1, DNM1L) and metabolic enzymes involved in glycolysis, the tricarboxylic acid cycle and oxidative metabolism.

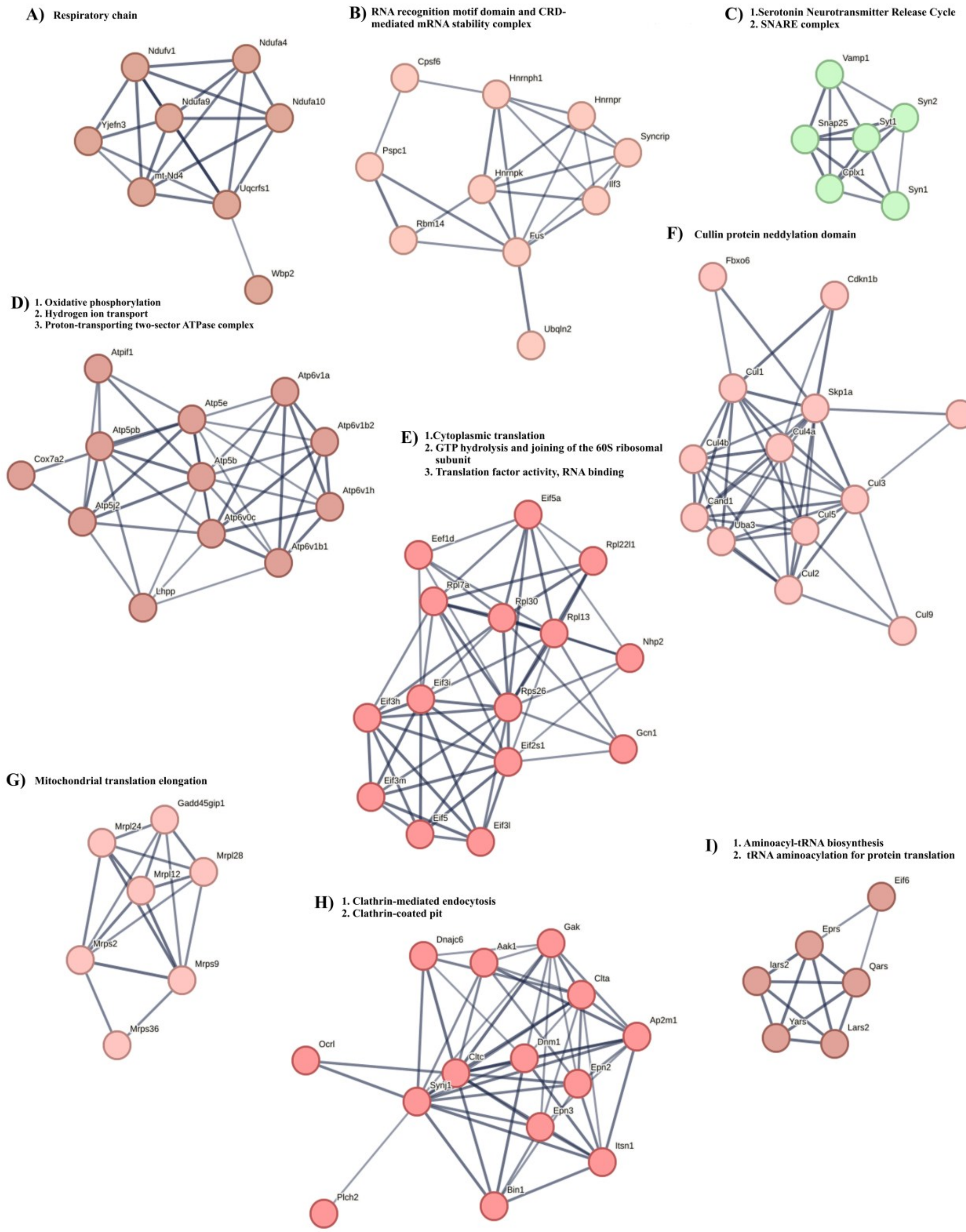

Supplementary Figure 4. STRING cluster analysis identifies discrete functional clusters within the dysregulated proteome following *fem*CSDS. (A–F) Major clusters derived from the downregulated proteome, including **(A)** respiratory chain proteins, **(B)** RNA recognition motif domain and CRD-mediated mRNA stability complex, **(C)** serotonin neurotransmitter release cycle and SNARE complex proteins, **(D)** oxidative phosphorylation and ATP synthase machinery, **(E)** cytoplasmic translation and ribosomal initiation factors and **(F)** Cullin-dependent ubiquitin ligase/neddylation machinery. **(G–I)** Major clusters derived from the upregulated proteome, including **(G)**

mitochondrial translation elongation proteins, **(H)** clathrin-mediated endocytosis and vesicle trafficking proteins, and **(I)** aminoacyl-tRNA biosynthesis and tRNA aminoacylation proteins. Nodes represent individual proteins and edges represent experimentally validated or predicted protein-protein interactions identified by STRING.

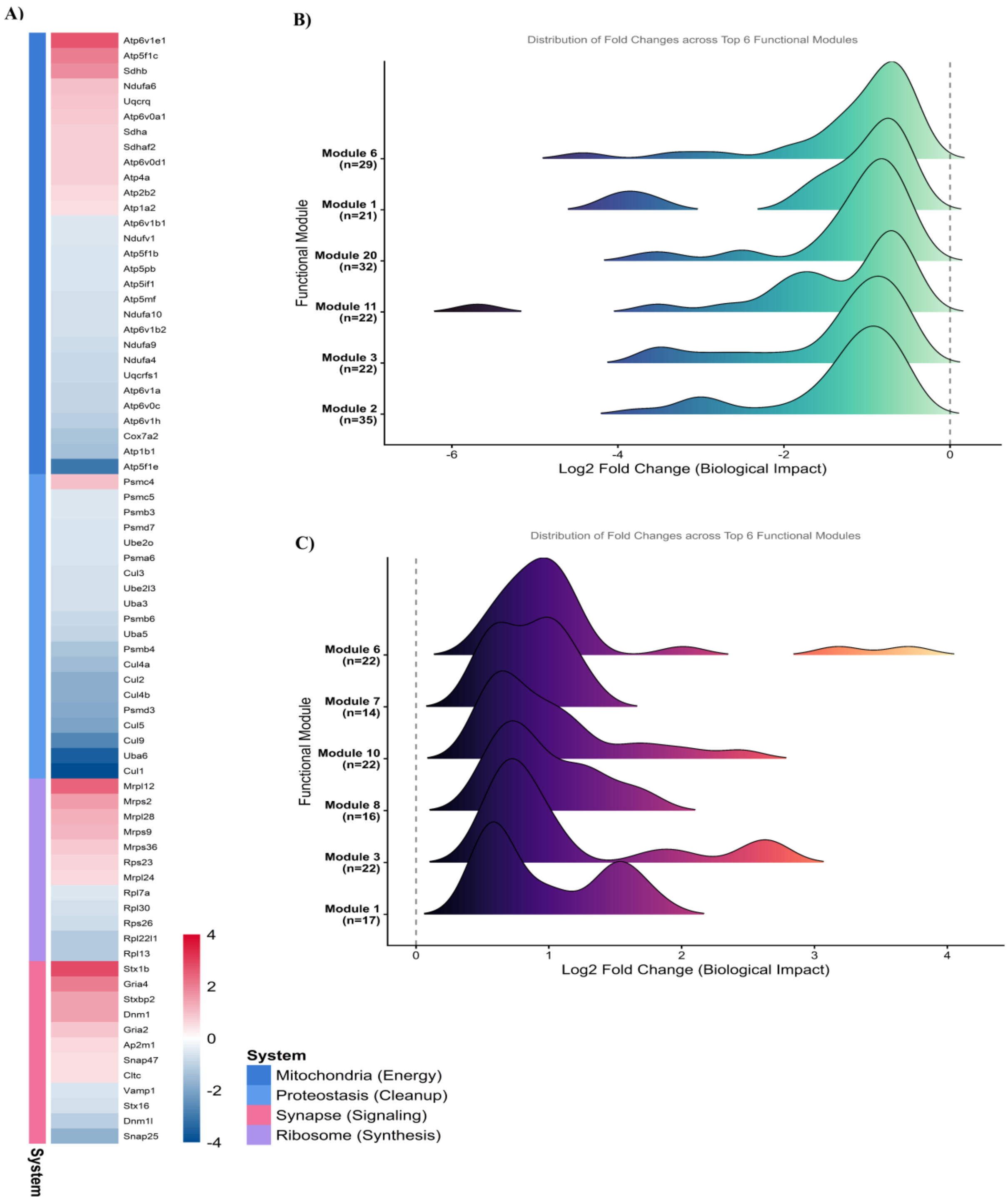

Supplementary Figure 5. Integrative module-impact analysis highlights suppression of mitochondrial and proteostasis systems with concurrent activation of synaptic and translational programs following *fem*CSDS. (A) Heatmap showing representative proteins from major dysregulated biological systems identified in the female nucleus accumbens following chronic social defeat stress (*fem*CSDS). Proteins are grouped according to four principal functional systems: mitochondrial energy metabolism, proteostasis/ubiquitin-proteasome pathways, ribosomal/translation machinery and synaptic signaling networks. Color intensity represents Log2 fold change. **(B)** Distribution of protein fold changes across the six most prominent downregulated functional modules identified by STRING-Louvain clustering. Density plots demonstrate a strong leftward shift in module activity, indicating

coordinated suppression of mitochondrial respiration, oxidative phosphorylation, ATP synthesis and proteostasis-related pathways. **(C)** Distribution of protein fold changes across the six most prominent upregulated functional modules. Density plots show a rightward shift in module activity, reflecting coordinated upregulation of synaptic signaling, vesicle trafficking, endocytosis, neurotransmission and translational adaptation pathways.

A)

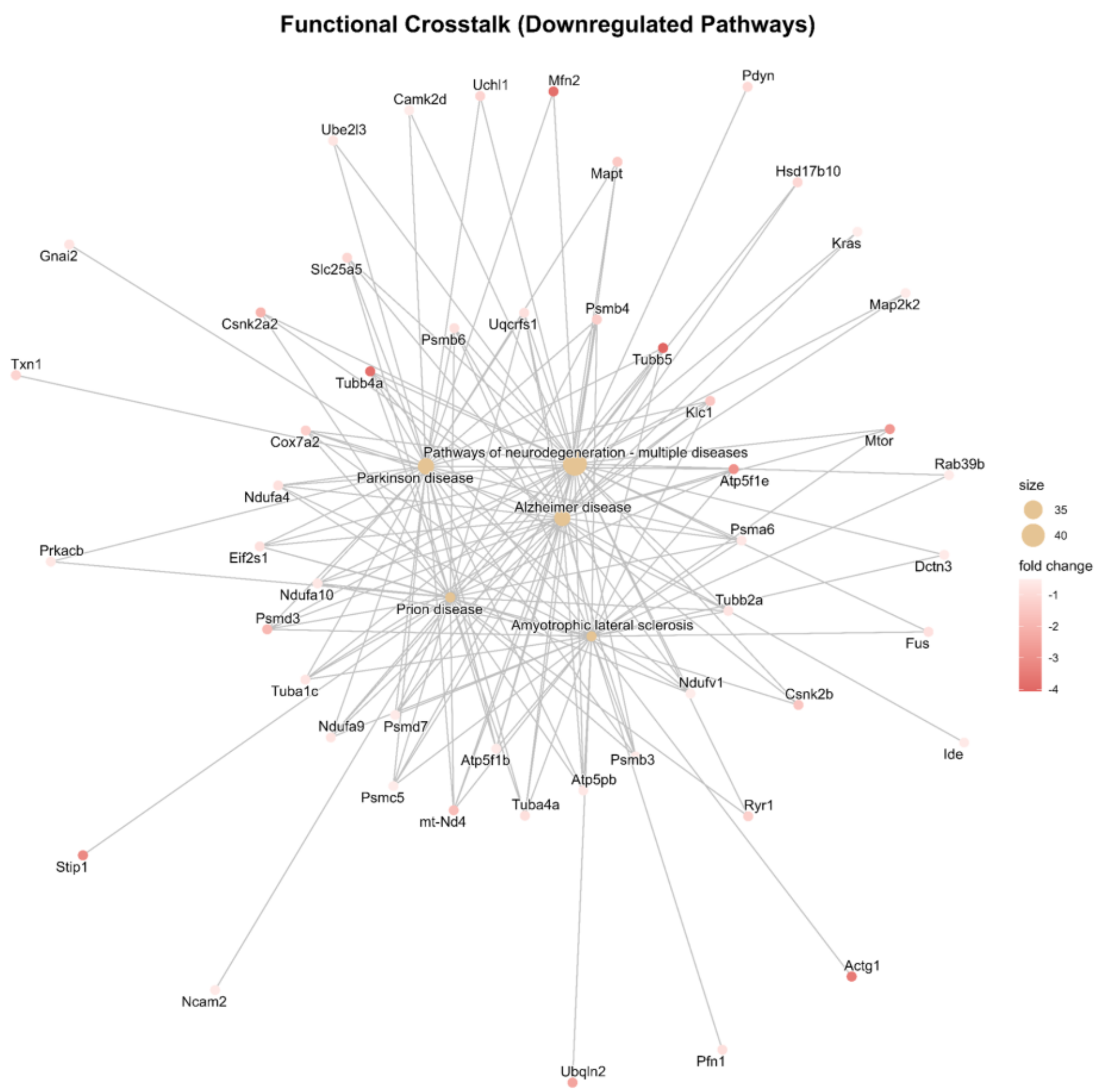

Functional Crosstalk (Downregulated Pathways)
Pathways of neurodegeneration - multiple diseases
Parkinson disease
Alzheimer disease
Prion disease
Amyotrophic lateral sclerosis
Uchl1
Mfn2
Pdyn
Camk2d
Ube2l3
Mapt
Hsd17b10
Kras
Gnai2
Slc25a5
Map2k2
Csnk2a2
Uqcrfs1
Psmb4
Psmb6
Txn1
Tubb5
Tubb4a
Klc1
Cox7a2
Mtor
Rab39b
Atp5f1e
Ndufa4
Psma6
Prkacb
Eif2s1
Dctn3
Ndufa10
Tubb2a
Psmd3
Fus
Tuba1c
Ndufv1
Csnk2b
Ndufa9
Psmd7
Ide
Atp5f1b
Psmb3
Atp5pb
Psmc5
Tuba4a
Ryr1
mt-Nd4
Stip1
Actg1
Ncam2
Pfn1
Ubqln2
size
35
40
fold change
-1
-2
-3
-4

B)

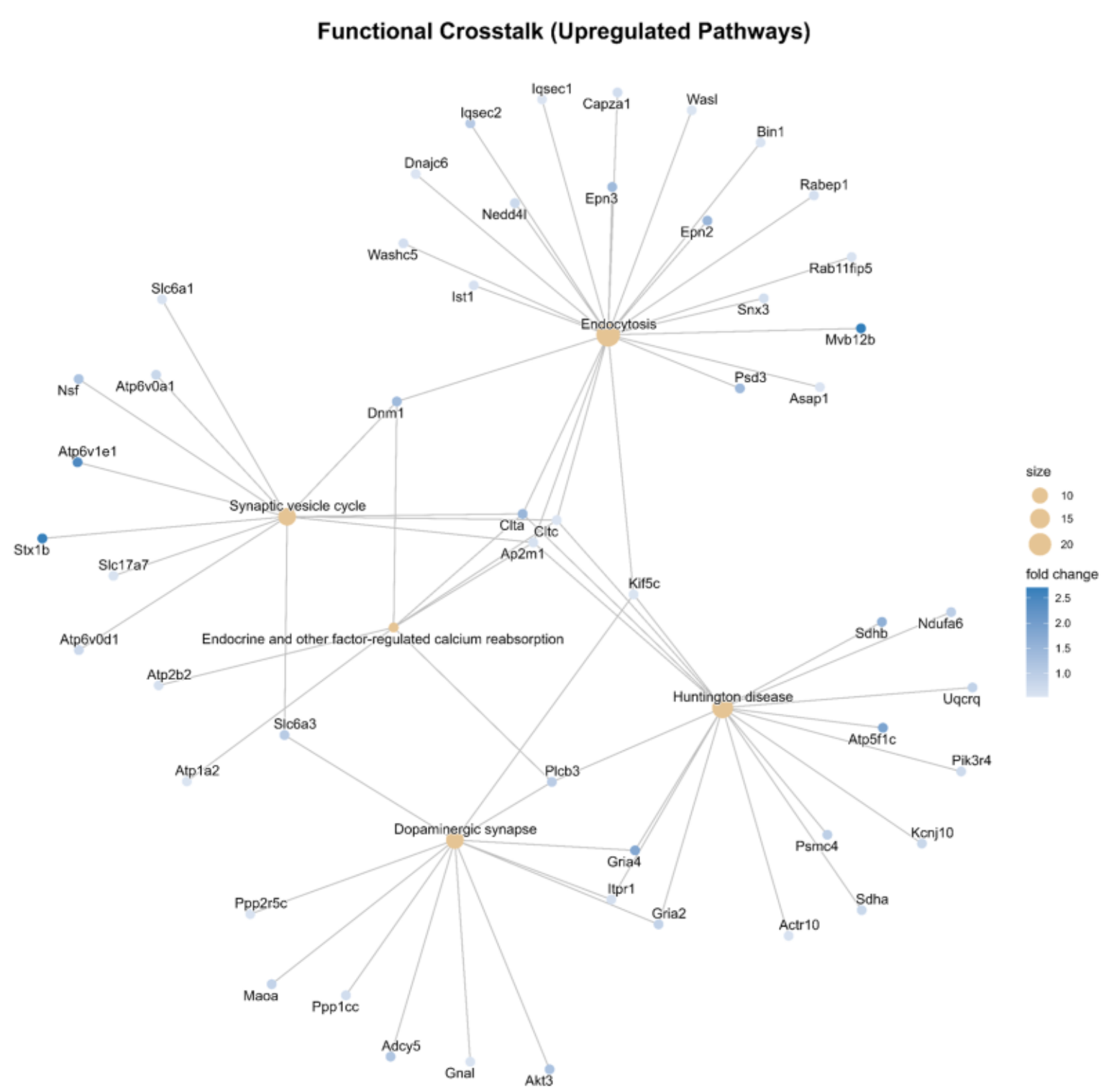

Functional Crosstalk (Upregulated Pathways)
Iqsec1
Capza1
Wasl
Iqsec2
Bin1
Dnajc6
Epn3
Rabep1
Nedd4l
Epn2
Washc5
Rab11fip5
Ist1
Snx3
Endocytosis
Mvb12b
Slc6a1
Psd3
Asap1
Nsf
Atp6v0a1
Dnm1
Atp6v1e1
Synaptic vesicle cycle
Clta
Cltc
Stx1b
Ap2m1
Slc17a7
Kif5c
Sdhb
Ndufa6
Atp6v0d1
Endocrine and other factor-regulated calcium reabsorption
Atp2b2
Huntington disease
Uqcrq
Slc6a3
Atp5f1c
Pik3r4
Atp1a2
Plcb3
Dopaminergic synapse
Kcnj10
Psmc4
Gria4
Itpr1
Sdha
Ppp2r5c
Gria2
Actr10
Maoa
Ppp1cc
Adcy5
Gnal
Akt3
size
10
15
20
fold change
2.5
2.0
1.5
1.0

Supplementary Figure 6. Functional crosstalk networks. (A) Gene-concept network illustrating relationships between significantly downregulated proteins and enriched KEGG pathways. Large central nodes represent enriched biological pathways, whereas peripheral nodes represent individual proteins contributing to pathway enrichment. Node size reflects the number of proteins associated with each pathway and protein node color intensity corresponds to the magnitude of downregulation (Log2 fold change). Downregulated proteins converged predominantly on pathways associated with Parkinson disease, Alzheimer disease amyotrophic lateral sclerosis, prion disease and pathways of neurodegeneration–multiple diseases, highlighting extensive suppression of mitochondrial respiration, proteostasis, cytoskeletal organization and neuronal maintenance programs. (B) Gene–concept network illustrating relationships between significantly upregulated proteins and enriched KEGG pathways. Large nodes indicate enriched pathways and smaller nodes represent contributing proteins. Upregulated proteins were primarily associated with endocytosis, synaptic vesicle cycle, dopaminergic synapse, endocrine and other factor-regulated calcium reabsorption and Huntington disease pathways, indicating activation of vesicle trafficking, membrane recycling, neurotransmission and calcium-dependent synaptic remodeling processes.

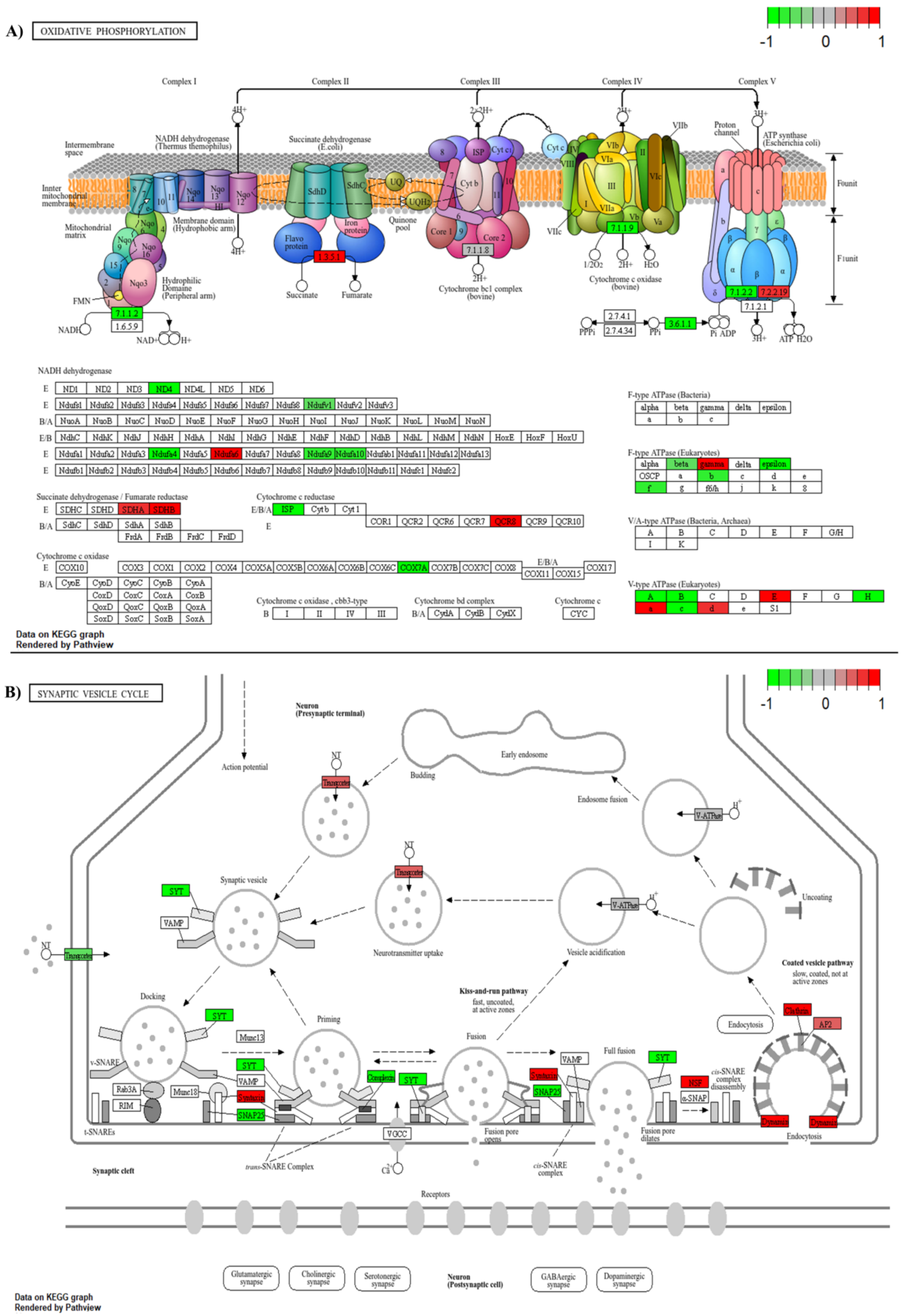

A)
OXIDATIVE PHOSPHORYLATION
-1 0 1
Complex I
Complex II
Complex III
Complex IV
Complex V
Intermembrane space
Inner mitochondrial membrane
Mitochondrial matrix
NADH dehydrogenase (Thermus themophilus)
Succinate dehydrogenase (E.coli)
Membrane domain (Hydrophobic arm)
Hydrophilic Domain (Peripheral arm)
Quinone pool
Cytochrome bc1 complex (bovine)
Cytochrome c oxidase (bovine)
Proton channel
ATP synthase (Escherichia coli)
Founit
F1unit
NADH
NAD+
H+
FMN
7.1.1.2
1.6.5.9
1.3.5.1
7.1.1.8
7.1.1.9
7.1.2.2
7.2.2.19
7.1.2.1
2.7.4.1
2.7.4.34
3.6.1.1
Succinate
Fumarate
Pi ADP
ATP H2O
NADH dehydrogenase
Succinate dehydrogenase / Fumarate reductase
Cytochrome c reductase
Cytochrome c oxidase
Cytochrome c oxidase , cbb3-type
Cytochrome bd complex
Cytochrome c
F-type ATPase (Bacteria)
F-type ATPase (Eukaryotes)
V/A-type ATPase (Bacteria, Archaea)
V-type ATPase (Eukaryotes)
Data on KEGG graph
Rendered by Pathview
B)
SYNAPTIC VESICLE CYCLE
-1 0 1
Neuron (Presynaptic terminal)
Action potential
Budding
Early endosome
Endosome fusion
Synaptic vesicle
Neurotransmitter uptake
Vesicle acidification
Uncoating
Coated vesicle pathway
slow, coated, not at active zones
Docking
Priming
Kiss-and-run pathway
fast, uncoated, at active zones
Fusion
Full fusion
Endocytosis
SYT
VAMP
Munc13
Munc18
Rab3A
RIM
SNAP25
Syntaxin
Complexin
VGCC
NSF
α-SNAP
Clathrin
AP2
Dynamin
V-ATPase
Transporter
v-SNARE
t-SNAREs
trans-SNARE Complex
cis-SNARE complex
cis-SNARE complex disassembly
Fusion pore opens
Fusion pore dilates
Synaptic cleft
Receptors
Glutamatergic synapse
Cholinergic synapse
Serotonergic synapse
Neuron (Postsynaptic cell)
GABAergic synapse
Dopaminergic synapse
Data on KEGG graph
Rendered by Pathview

Supplementary Figure 7. KEGG pathway mapping of oxidative phosphorylation and synaptic vesicle cycle proteins altered by femCSDS. (A) KEGG oxidative phosphorylation pathway overlaid with proteomic expression data from the female nucleus accumbens following *fem*CSDS. Colored nodes represent proteins identified in the dataset and mapped onto the electron transport chain and ATP synthase complexes. Green indicates upregulation and red indicates downregulation according to the scale shown. Multiple subunits of Complex I (NDUF family), Complex II (SDH family), Complex III (UQCR family), Complex IV (COX family) and Complex V (ATP synthase subunits) exhibited reduced expression, indicating widespread suppression of mitochondrial respiratory chain activity and ATP-generating machinery. These findings support the bioenergetic impairment identified by Gene Ontology, STRING, SynGO and IPA analyses. **(B)** KEGG synaptic vesicle cycle pathway overlaid with proteomic expression data from the female nucleus accumbens following femCSDS. Several proteins involved in vesicle docking, fusion, endocytosis, and vesicle recycling, including SYT1, SNAP25, VAMP1, SYNJ1, DNM1 and clathrin-associated adaptor proteins, displayed altered expression. The predominance of upregulated trafficking and vesicle-cycle components suggests activation of compensatory synaptic remodeling and neurotransmitter release pathways in response to chronic stress-induced neuronal dysfunction.

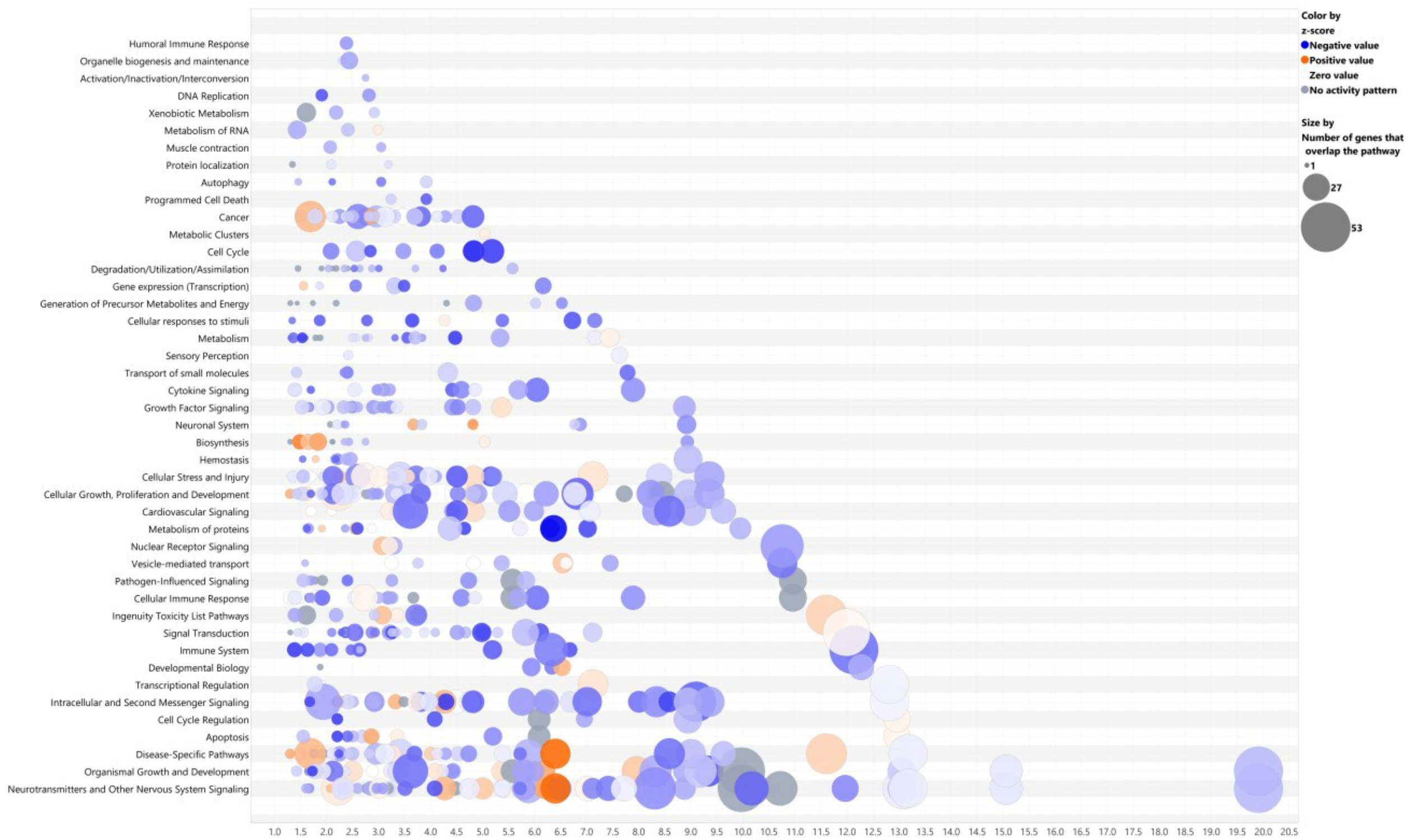

Supplementary Figure 8. Canonical pathway landscape generated from Ingenuity Pathway Analysis (IPA) of proteins altered in the female nucleus accumbens following *fem*CSDS. Bubble plot summarizing the major biological functions, signaling pathways and disease-associated categories identified by IPA from the differentially expressed proteome. Each bubble represents an enriched pathway or functional annotation. The x-axis indicates pathway significance expressed as −log10(p-value), while pathway categories are grouped along the y-axis. Bubble size corresponds to the number of dataset proteins mapped to each pathway, and bubble color represents the predicted activation state based on the IPA z-score algorithm, where orange indicates predicted activation, blue indicates predicted inhibition, white indicates no directional prediction and gray indicates insufficient information to determine activation status. Notably, pathways associated with neuronal function, neurotransmission, cellular metabolism, stress responses and energy production were among the most significantly enriched categories, supporting widespread molecular remodeling within the female nucleus accumbens following chronic social defeat stress.

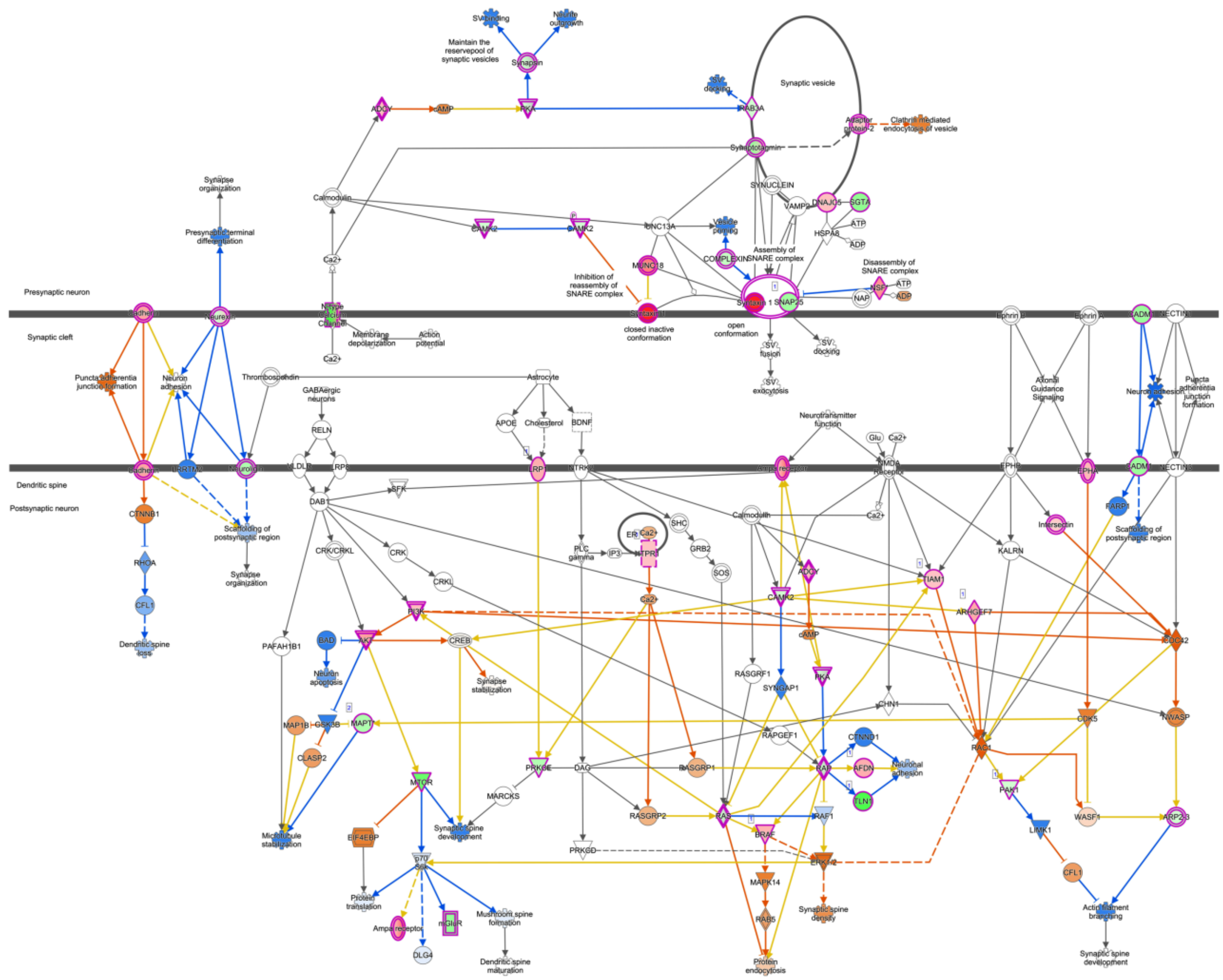

Supplementary Figure 9. Dysregulation of the Synaptogenesis Signaling Pathway in *fem*CSDS. The graphical representation visualizes expression changes of molecules: red nodes indicate increased expression, and green nodes denote decreased expression, with color intensity reflecting the magnitude of the change. Within this pathway, core presynaptic components crucial for neurotransmitter release were downregulated, including SNAP25 (log ratio = -1.709), SYN1 (log ratio = -0.849) and SYN2 (log ratio = -0.897), suggesting impaired vesicle fusion. Counteracting this, STX1B (Syntaxin 1B) also involved in synaptic vesicle fusion, showed a substantial upregulation (log ratio = 2.838), potentially indicating a compensatory response or altered synaptic organization. Further, MTOR, a central regulator of synaptic plasticity, was significantly downregulated (log ratio = -2.785), while AMPA receptor subunits GRIA2 (log ratio = 0.90) and GRIA4 (log ratio = 2.008) were upregulated, suggesting altered postsynaptic sensitivity. Other critical changes included decreased CACNB1 (log ratio = -3.658) and TLN1 (log ratio = -3.462), impacting neuronal excitability and cytoskeletal integrity. Predicted relationships between molecules are shown by lines, with solid lines indicating direct and dashed lines indirect interactions and colors denoting predicted activation (orange) or inhibition (blue).

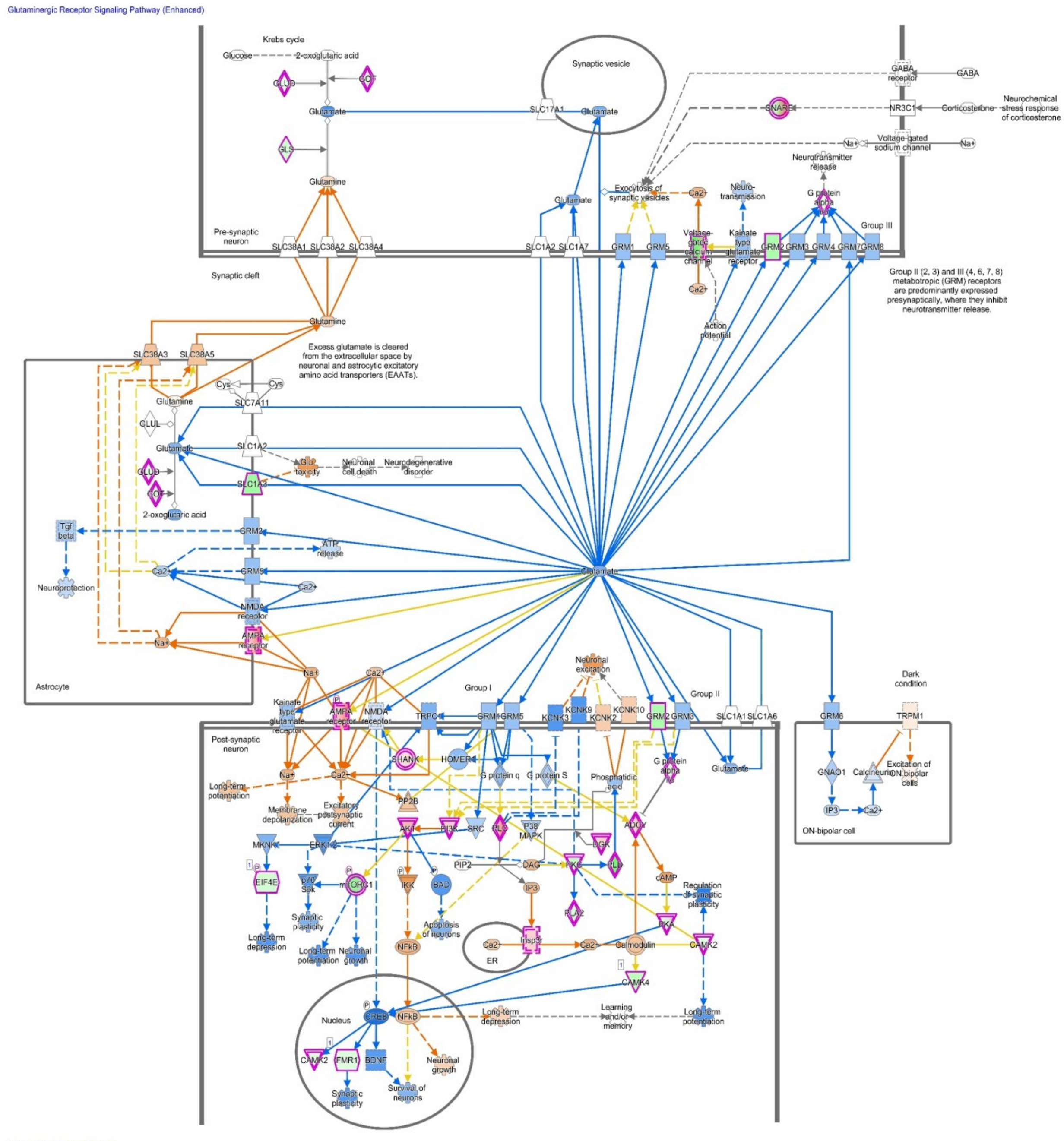

© 2000-2024 QIAGEN. All rights reserved.

Supplementary Figure 10. IPA glutaminergic receptor signaling pathway analysis. Canonical pathway map generated by Ingenuity Pathway Analysis (IPA) showing proteins from the *fem*CSDS dataset mapped onto the Glutaminergic Receptor Signaling Pathway.

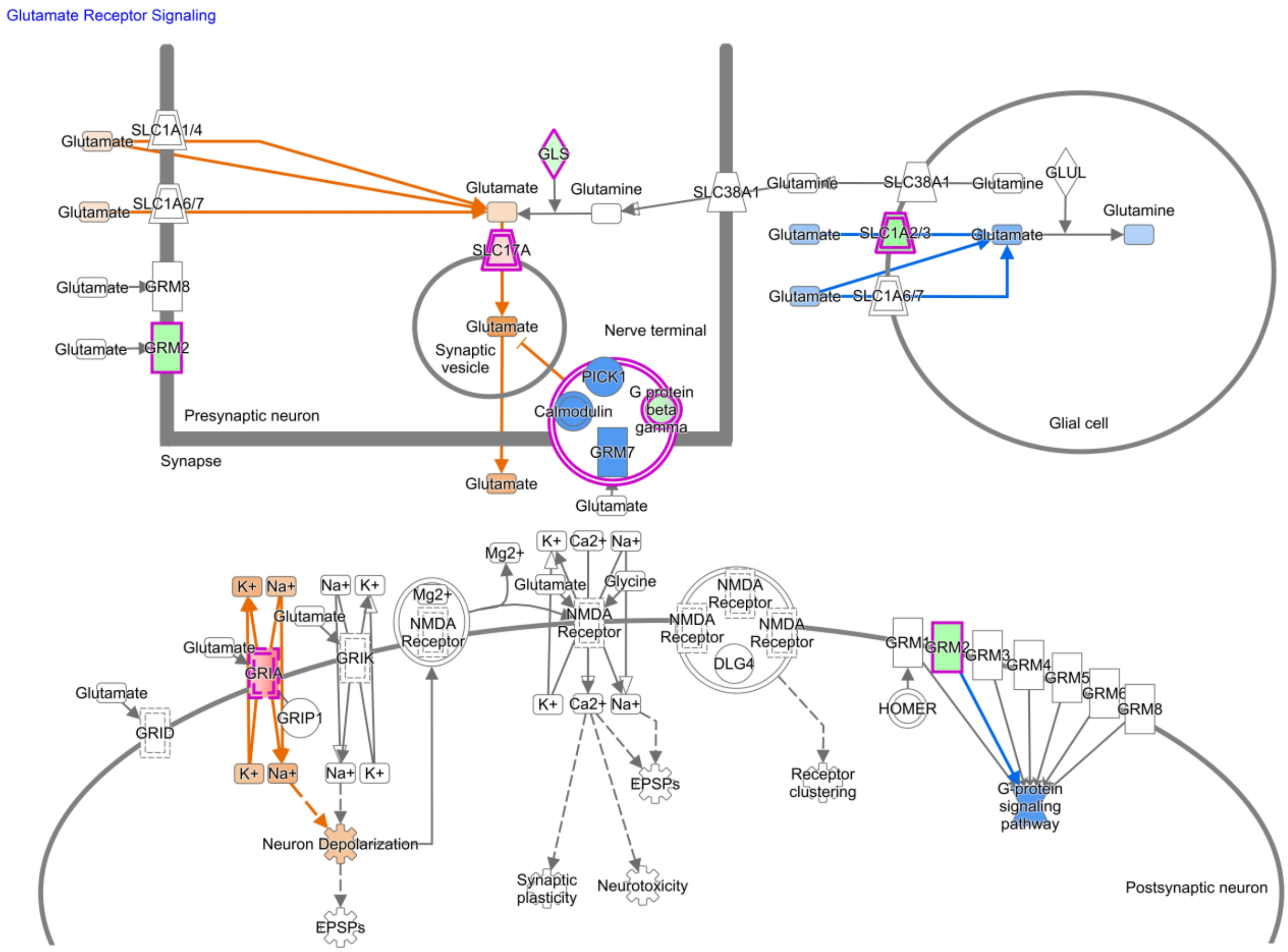

Supplementary Figure 11. IPA enhanced glutamatergic receptor signaling pathway analysis. Expanded IPA pathway representation of Glutamatergic Receptor Signaling (Enhanced) showing the integration of altered proteins with downstream signaling networks regulating neuronal excitability, synaptic transmission, calcium signaling, long-term potentiation and synaptic plasticity. Differentially expressed pathway components support activation of adaptive glutamatergic remodeling programs in the female nucleus accumbens following *fem*CSDS.

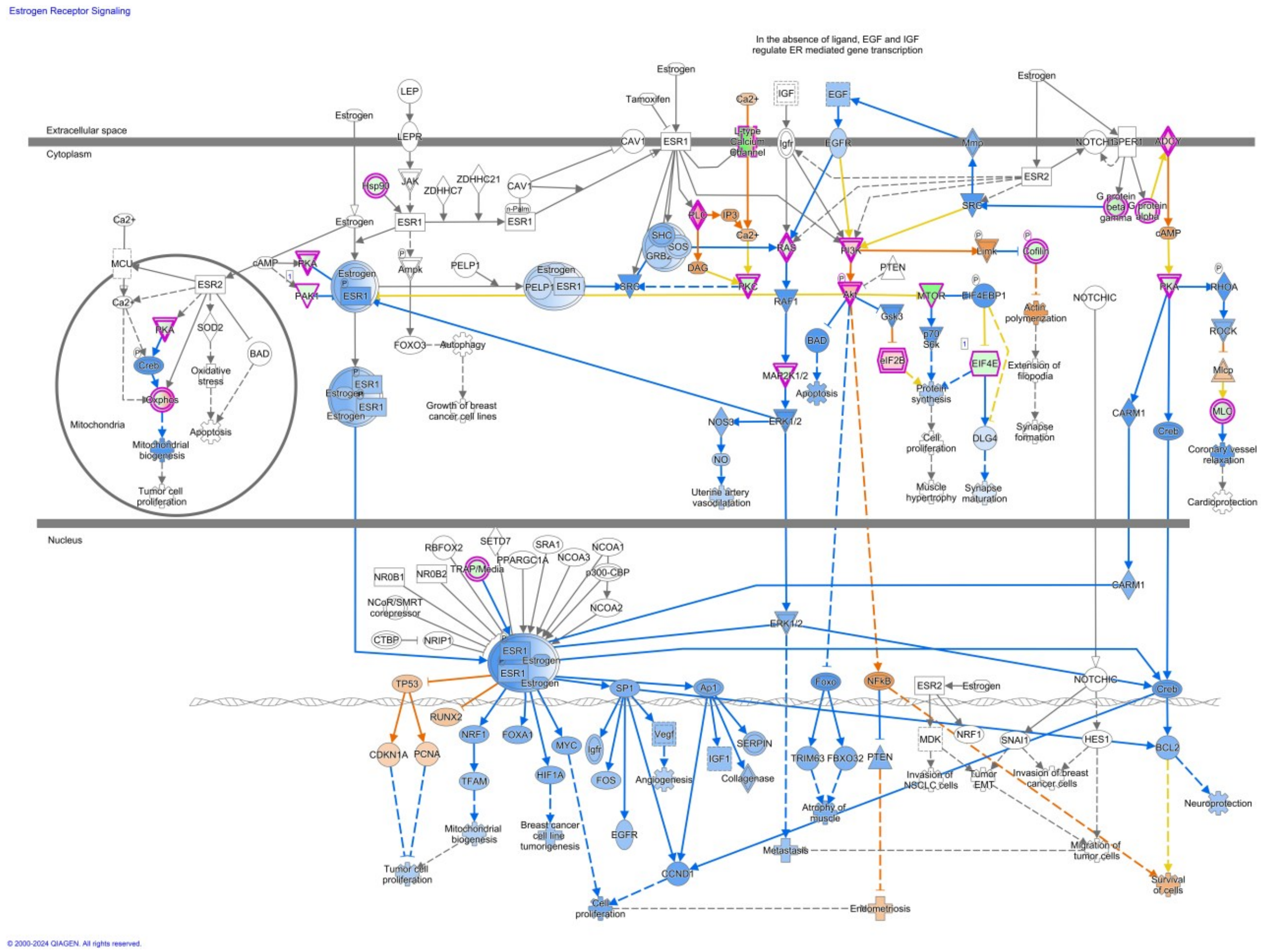

Supplementary Figure 12. IPA estrogen receptor signaling pathway analysis. Canonical pathway map generated by IPA illustrating proteins associated with Estrogen Receptor Signaling identified in the *fem*CSDS proteomic dataset. Differentially expressed proteins were mapped to pathways involved in estrogen-mediated transcriptional regulation, cellular signaling, mitochondrial function, neuroprotection and synaptic plasticity.

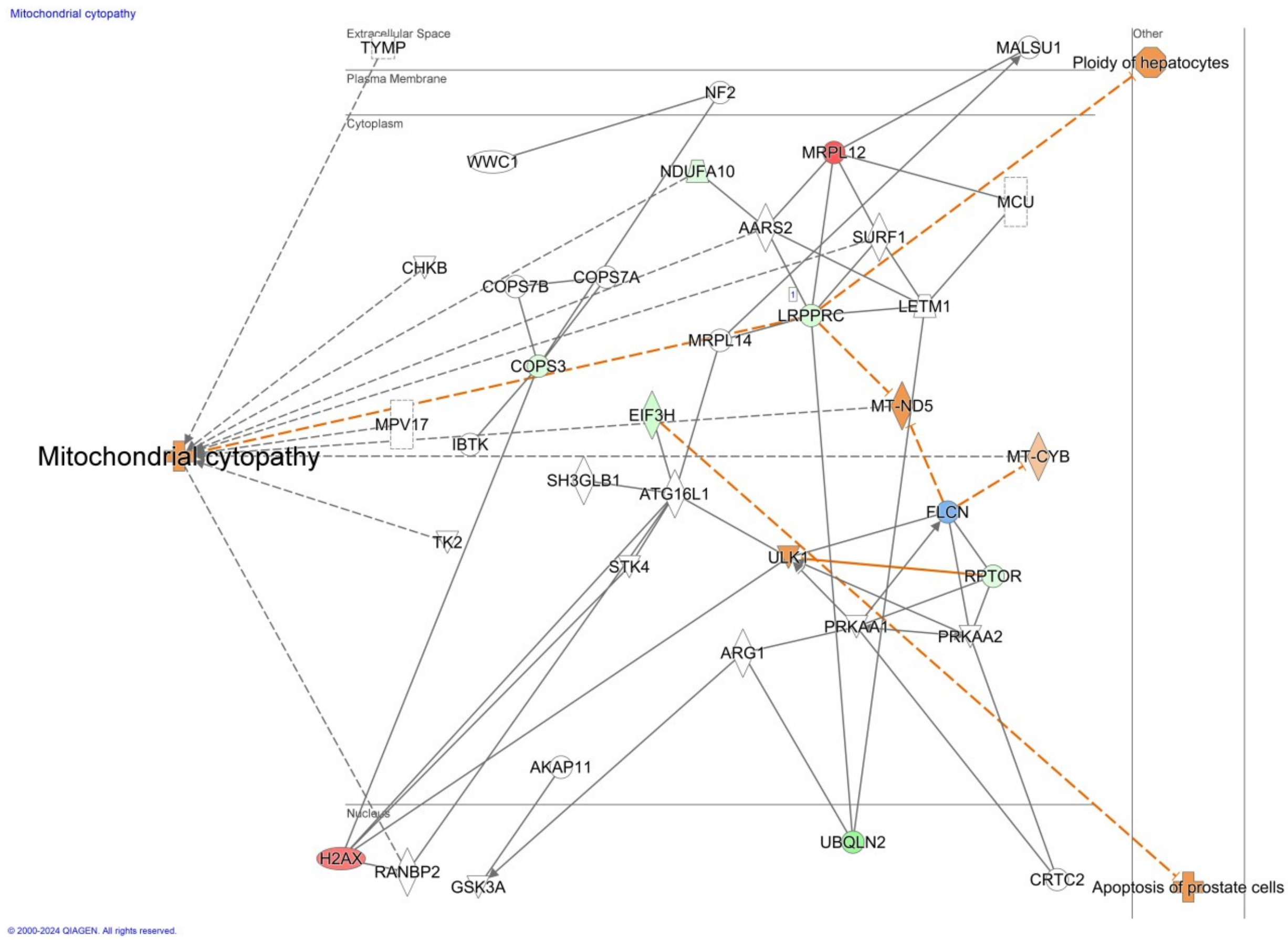

Supplementary Figure 13. IPA disease and function network associated with mitochondrial cytopathy. Disease and function network generated by Ingenuity Pathway Analysis (IPA) highlighting proteins from the *fem*CSDS dataset associated with the annotation "Mitochondrial Cytopathy." Differentially expressed proteins were mapped onto interconnected pathways involved in mitochondrial translation, oxidative phosphorylation, mitochondrial quality control, autophagy–mitophagy signaling, energy metabolism and cellular stress responses. Colored molecules represent proteins identified in the dataset, with red indicating increased abundance and green indicating decreased abundance. Network analysis identified altered expression of mitochondrial ribosomal proteins (MRPL12, MRPL14), electron transport chain components (NDUFA10), mitochondrial regulatory proteins (LRPPRC, LETM1, SURF1, MCU) and proteostasis-associated factors (UBQLN2), supporting widespread disruption of mitochondrial homeostasis. The network further links these molecular alterations to mitochondrial dysfunction, impaired bioenergetic capacity and cellular stress pathways, suggesting the role of mitochondrial pathology in the female nucleus accumbens following chronic social defeat stress.

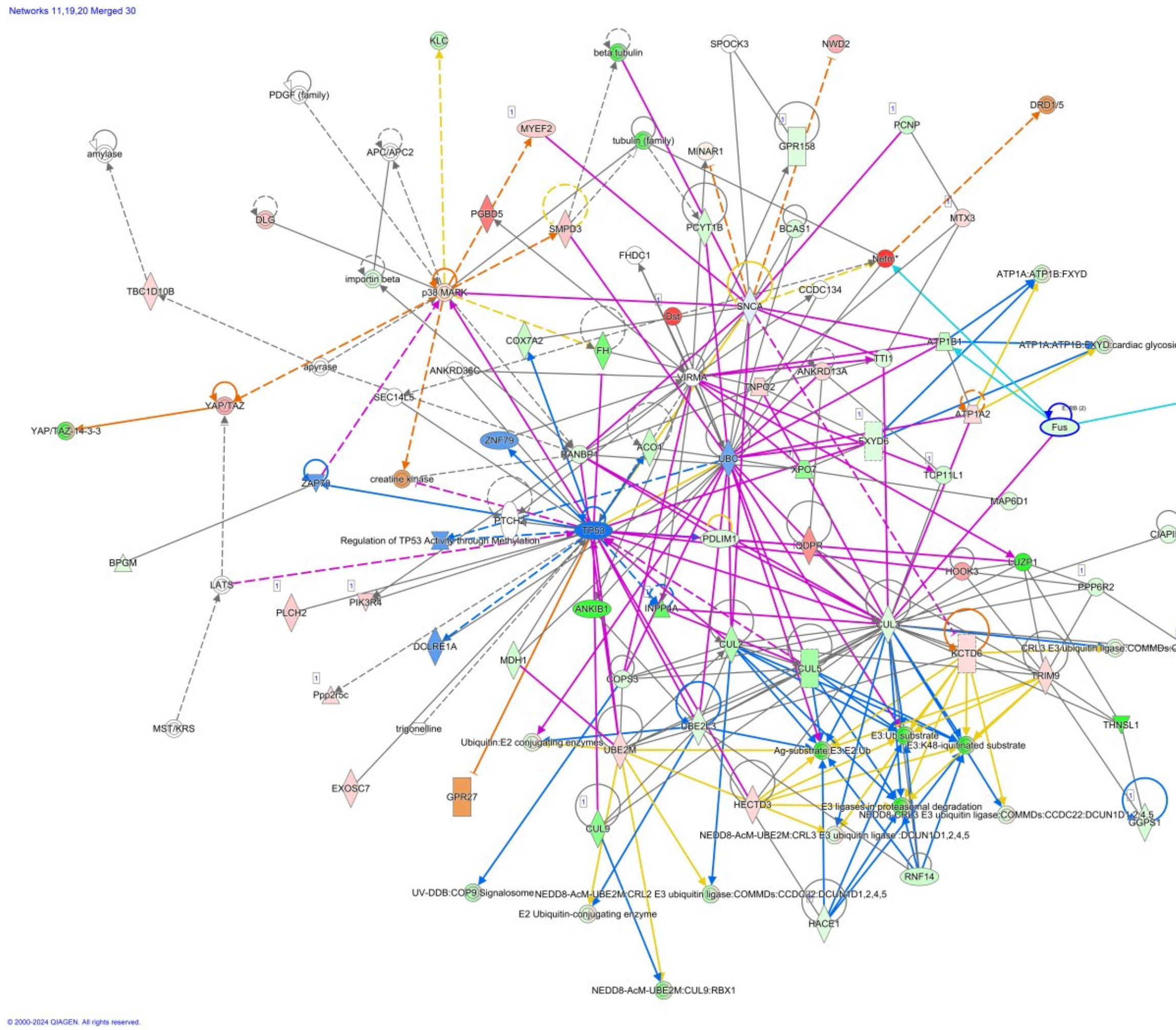

Supplementary Figure 14. Integrated IPA molecular interaction network highlighting stress-responsive, proteostatic and signaling pathways altered following *fem*CSDS. Integrated network generated by Ingenuity Pathway Analysis (IPA) from merged high-scoring interaction networks identified in the female nucleus accumbens following chronic social defeat stress. The network reveals extensive connectivity among proteins involved in ubiquitin-mediated proteostasis, cellular stress signaling, cytoskeletal organization, mitochondrial regulation and neuronal homeostasis. Central hubs include TP53, SNCA, CUL5, UBC, ATP1A2, ATP1B4 and NEDD8-associated ubiquitin ligase complexes. Multiple interactions converge on Cullin-RING ubiquitin ligase machinery, ubiquitin-conjugating enzymes and TP53-associated signaling pathways. Colored molecules represent proteins identified in the dataset and are displayed according to their direction of regulation within the proteomic analysis.

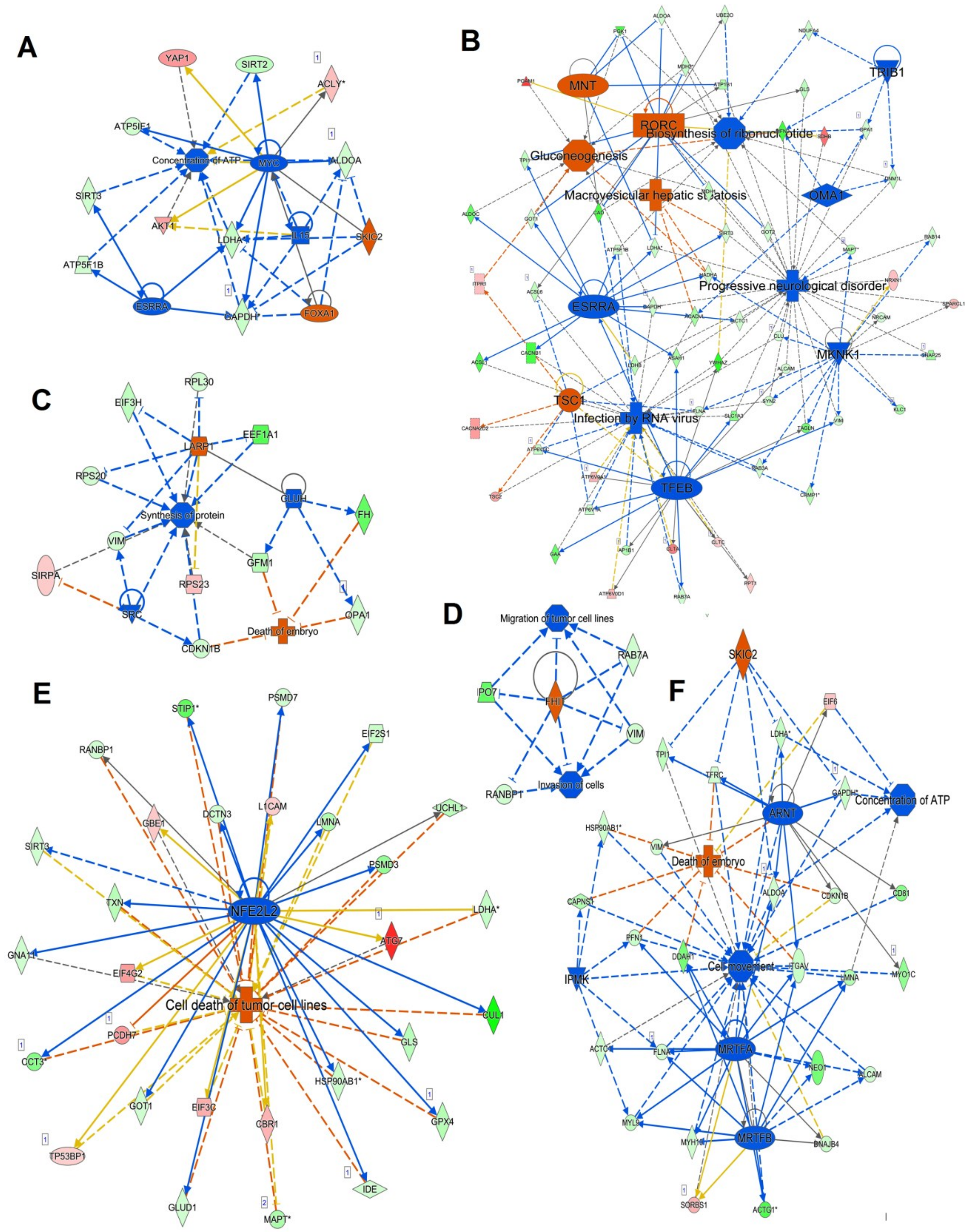

Supplementary Figure 15. IPA regulatory networks associated with major upstream regulators identified in the female nucleus accumbens following *fem*CSDS. Ingenuity Pathway Analysis (IPA) generated mechanistic interaction networks centered on key predicted upstream regulators identified from the differentially expressed

proteome. Networks illustrate direct and indirect relationships between regulators and downstream protein targets associated with mitochondrial function, cellular metabolism, protein synthesis, oxidative stress responses, cytoskeletal remodeling and neuronal plasticity. (A) MYC-centered network, highlighting interactions linked to ATP homeostasis, glycolysis and metabolic regulation. (B) ESRRA-centered network, showing associations with mitochondrial bioenergetics, metabolic pathways and stress-responsive signaling. (C) larp1-centered network, connected to translational control and protein synthesis pathways. (D) TFEB-centered network, associated with cellular homeostasis, lysosomal-autophagic processes and stress adaptation. (E) NFE2L2 (NRF2)-centered network, illustrating regulation of antioxidant defense and cytoprotective pathways. (F) ARNT2/MRTF signaling network, linking stress-responsive transcriptional regulation with cytoskeletal organization, cellular movement and neuronal adaptation. Molecules are colored according to expression changes observed in the proteomic dataset, with network architecture inferred from the IPA Knowledge Base.

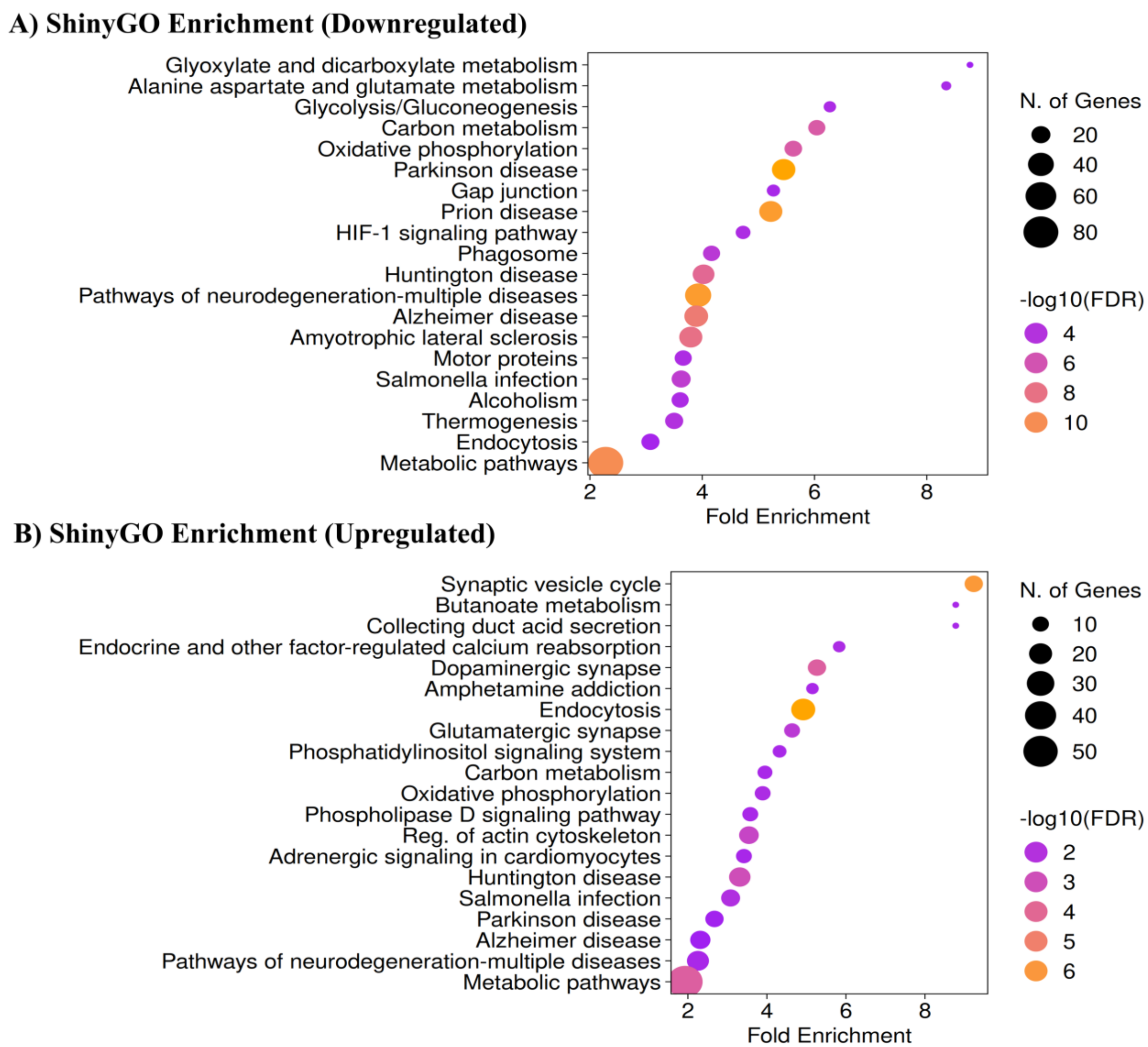

Supplementary Figure 16. ShinyGO pathway enrichment analysis of differentially expressed proteins following *fem*CSDS in the female nucleus accumbens. (A) ShinyGO pathway enrichment analysis of downregulated proteins. Enriched pathways were predominantly associated with cellular metabolism and mitochondrial function, including glyoxylate and dicarboxylate metabolism, alanine, aspartate and glutamate metabolism, glycolysis/gluconeogenesis, carbon metabolism, oxidative phosphorylation, HIF-1 signaling,

thermogenesis and pathways related to neurodegenerative disorders. The x-axis represents fold enrichment. Bubble size indicates the number of proteins contributing to each pathway, while color intensity represents statistical significance (-log10 FDR). (B) ShinyGO pathway enrichment analysis of upregulated proteins. Enriched pathways were primarily associated with synaptic signaling and neuronal plasticity, including synaptic vesicle cycle, dopaminergic synapse, glutamatergic synapse, endocytosis, phosphatidylinositol signaling, regulation of actin cytoskeleton and calcium-dependent signaling pathways. Additional enrichment was observed in vesicle trafficking and membrane remodeling processes.
